# Optimizing health facility location for universal health care: A case study from the Philippines

**DOI:** 10.1371/journal.pone.0256821

**Published:** 2021-09-09

**Authors:** Lorenzo Jaime Yu Flores, Ramon Rafael Tonato, Gabrielle Ann dela Paz, Valerie Gilbert Ulep

**Affiliations:** 1 Department of Statistics and Data Science, Yale University, New Haven, Connecticut, United States of America; 2 Health Facility Development Bureau, Department of Health, Manila, Philippines; 3 Philippine Institute for Development Studies, Quezon City, Philippines; Shenzhen University, CHINA

## Abstract

Site selection of health facilities is critical in ensuring universal access to basic healthcare services. However, in many low and middle-income countries (LMICs) like the Philippines, site selection is traditionally based on political and pragmatic considerations. Moreover, literature that demonstrates the application of facility location models in the Philippine healthcare setting remains scarce, and their usage in actual facility planning is even more limited. In this study, we proposed a variation of cooperative covering maximal models to identify the optimal location of primary care facilities. We demonstrated the feasibility of implementing such a model by using open source data on an actual city in the Philippines. Our results generated multiple candidate locations of primary care facilities depending on the equity and efficiency parameters. This approach could be used as one of the critical considerations in evidence-based, multi-criterion health facility location decisions of governments, and can also be adapted in other industries, given the model’s use of readily available open source datasets.

## Introduction

Health facility location is a critical factor in strategic planning of healthcare programs [1, 2]. A well-placed health facility increases uptake of essential healthcare services and improves health outcomes especially among vulnerable populations [3, 4]. In many low and middle-income countries (LMICs), the decision to build health facilities is traditionally based on political and pragmatic considerations [5]. Consequently, the location of most health facilities is typically far from optimal [6]. In recent years, governments are now increasingly interested in studying where to build health facilities to facilitate the achievement of health system goals.

In the Philippines, access to basic healthcare services remains a major challenge. This is largely attributed to scarcity and maldistribution of health facilities in many parts of the country. About 50% of the population do not have access to primary care facilities (PCFs) within 30 minutes [S1 File]. To address this, the Philippine government passed a landmark legislation called the *Universal Healthcare (UHC) Act* in 2019, which outlined strategies for multiple demand and supply-side challenges that continued to impede universal access to essential healthcare services. One of the critical provisions of the law is to increase capital infrastructure investments in the medium to long-term. Relevant to the reform includes identifying optimal locations for new healthcare facilities, specifically primary care facilities (PCF) or *rural health units (RHUs)*, which are government-owned health facilities that provide basic and comprehensive healthcare services to individuals, families, and local communities. In this study, we focused mainly on primary care facilities to align with the goals of the *UHC law*, which involve augmenting the country’s primary healthcare system by the year 2025. Ultimately, the goal is to select and identify locations that serve the most people while still accounting for distance, hazards, and existence of other healthcare facilities.

In computer science, this task is known as the facility location problem (FLP), which has been adopted for many applications in healthcare, education, retail, etc. [1, 7–11]. Typically, models solve this problem by using algorithms that determine the best placement of a facility that optimizes for metrics such as least average travel time to a facility or most coverage within some radius, with examples shown in Table 1. The choice of model is based on the metrics that policy makers wish to optimize for. Therefore, there is no gold standard amongst facility location models, but rather a set of optimal locations chosen based on the priorities and goals of decision makers.

**Table 1:** Common models employed toward the facility location problem.

Model	Objective	Authors
P-Median Model	Minimize total travelling distance/time of each patient to their assigned facility	Hakimi et al [12]
Location Set Covering Model	Minimize number of facilities needed such that all patients are within an acceptable distance from a facility	Toregas and Revelle [13]
Maximal Location Covering Model	Maximize number of patients within acceptable distance given a fixed number of facilities to be built	Church and Revelle [14]
Hierarchical Covering Location Model	Variation of Location Set Covering Model employing multiple types of facilities with different acceptable distance values	Moore and Revelle [15]
Cooperative Covering Location Model	Variation of Location Set Covering Model assuming that demand at a location can be met using multiple facilities which attract patients based on distance	Berman et al [8]

Outside the Philippines, multiple studies have demonstrated the application of these models towards optimizing the location of healthcare facilities. The team of [16] demonstrated the use of the Capacitated Maximal Covering Problem in Kuala Langat, Malaysia, which maximized the number of people living within 3km and 5km of rural clinics while accounting for limitations in capacity of each facility. A similar study was performed by [17] for sexual health clinics in Hampshire, United Kingdom, where they showed that a greedy solution achieved optimal or near optimal solutions when compared to other complex solving algorithms. The team of [18] proposed a multiple-objective model for a case study in Hong Kong, which maximized the number of people living within 10km of a health facility and an accessibility metric, while minimizing building cost and inequity. The team of [19] further optimized for metrics such as service quality and environmental concerns in facility location on their case study on Mao County, Sichuan, China.

In such studies, the ability to develop models that accounted for the mentioned variables relied on the availability of data. Some studies employed assumptions in the modeling process, while others required city-specific data collected for the study. This may pose challenges in practical application in countries where this data is not yet readily available, like in the Philippines.

As of this paper’s writing, similar literature that demonstrated the feasibility of implementing such models in the Philippine healthcare context remains scarce. Previous work applied a hierarchical location model to determine optimal placements of barangay (i.e. village) level clinics in Davao City, Philippines [5]. However, the work operated under the assumptions that (1) there were no existing health facilities, (2) candidate facilities would be placed at the centroid of each barangay assuming population was concentrated there, (3) travel distance between points was modeled using Euclidean distance, and (4) demand was the same all throughout the region. While the lack of data at the time explains why such assumptions had to be made previously, the advent of remote sensing based population modeling and advances in geospatial software have made granular data readily accessible, thereby allowing researchers to address these assumptions. For example, travel times calculated using road networks and driving speeds are available through APIs such as Mapbox; population at a granularity of 100m x 100m is available through datasets such as the WorldPop dataset developed by the Center for International Earth Science Information Network [20]; and a list of Philippine health facilities can be accessed at the National Health Facility Registry by the Philippine department of health, and their coordinates can be obtained using the Google Geotagging API [21].

The mentioned open source datasets can be publicly audited, and are thus relatively secure. Moreover, such data has little to no overhead or long-term costs compared to proprietary software, which makes it more preferable and advantageous in LMIC settings. Since the Philippine health system is devolved and many data collection systems are fragmented, using open source data can make it easier for different local government units to access, evaluate, modify and employ this method at their perusal. However, literature that demonstrates the feasibility of combining and using such data towards the facility location problem in the Philippine healthcare system context remains scarce, and the practical application of facility location modeling in the context of health facility development remains limited.

To this end, we explored a solution to the facility location problem that incorporates these open source datasets into the cooperative maximal covering model, which maximizes the number of people covered by the facilities, given a fixed number of sites to be built [22]. In this model, multiple health facilities could be used to cover each site, and the number of people which a facility attracts depends on the attractiveness of a site. This attractiveness can be modeled by *distance decay*, that is, the relationship between the travel time to a site, and people’s willingness to visit it. We employed the results from a recent work which modeled distance decay using hospital patient visit records in Florida, USA [23].

In this paper, we made the following contributions. First, we proposed metrics for evaluating the location of a new primary care facility that incorporated results from recent healthcare literature. Second, we demonstrated the feasibility of using open source data to calculate and optimize such metrics on an actual city in the Philippines. Third, we compared the locations chosen by each method and identified its implications on issues of healthcare equity. Ultimately, we aimed to further the literature on facility location modeling in the Philippine healthcare system context by outlining an end-to-end framework for primary care facility site selection to assist in government policy making. Through the use of open source, granular datasets, we aim to develop a model that can address limitations in previous work, and one that can be replicated across multiple cities through the use of readily available open source data. Moreover, this model can be further modified to perform similar analyses for other health facilities.

## Methods

We used the open source datasets listed in Table 2 to conduct the analysis, and obtained the coordinates of PCFs in the National Health Facility Registry of the Philippine Department of Health (DOH) using the Google GeoTagging API. The Roads API provided the coordinates of the closest road segment to a given coordinate, based on existing road data in Google Maps. The Optimization API generated the driving time between two coordinates, based on the road segment’s length and given speed limits. The source code and datasets can be found at https://github.com/ljyflores/facility-location-philippines.

**Table 2:** Sources of datasets.

Variable	Dataset & Provider
Population	WorldPop Dataset [20]
Healthcare facilities	National Health Facility Registry [21]
Shapefiles	Philippine Subnational Administrative Boundaries [24]
Road networks	Roads API, Google Maps
Travel times	Optimization API, MapBox

**Fig 1:**
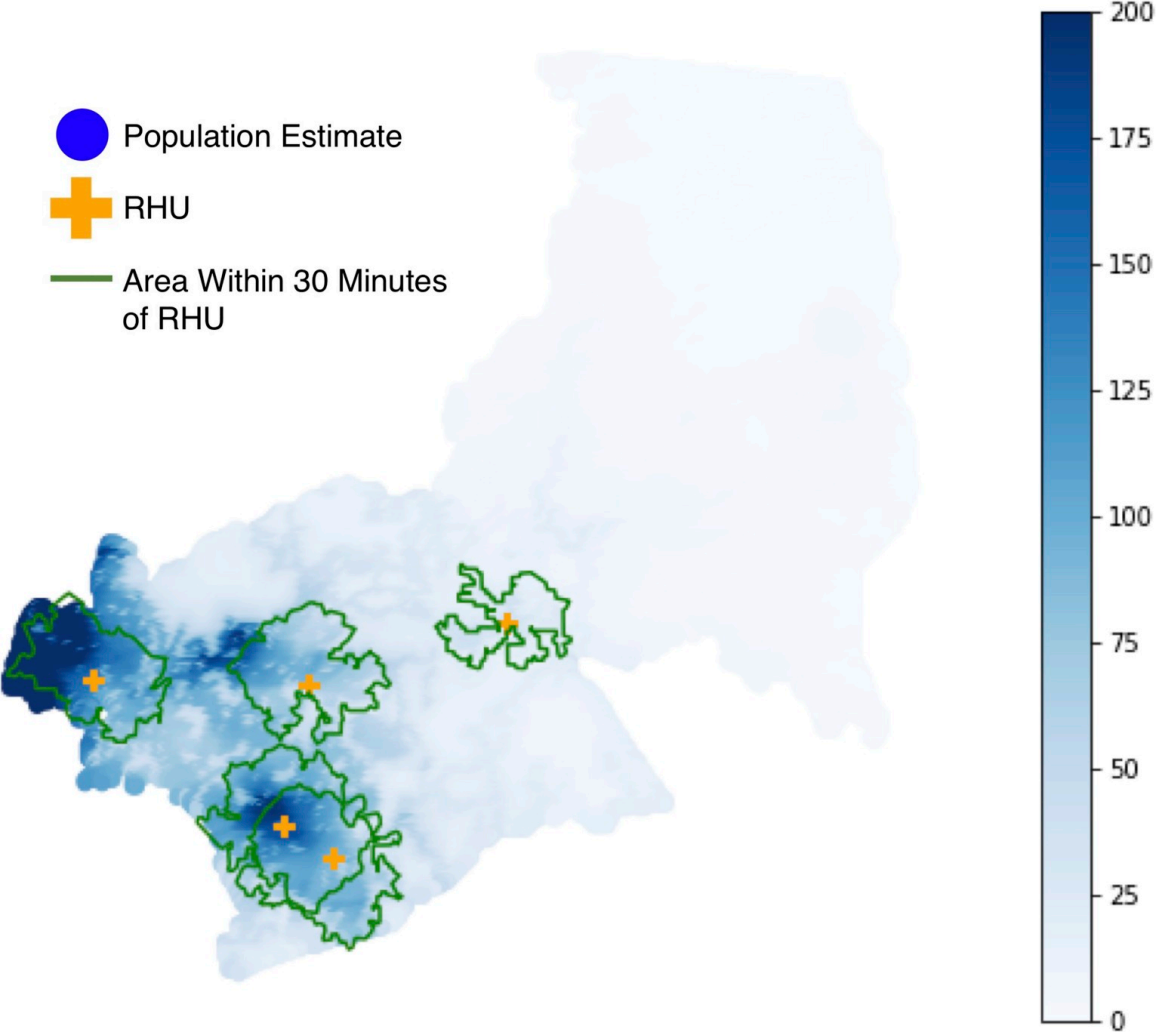
Overview of Antipolo City.

We chose Antipolo City, Philippines (2015 population: ~780,000 [25]) as a case study, as it comprises both urban and rural areas that highlight nuances between the proposed methods. Antipolo City is described as hilly and mountainous, with the hilly area in the west, and the mountainous areas in the east. Valleys are located in the urban area towards the southwest, and also in the south and north. Currently, there are 5 RHUs in Antipolo (Fig 1).

We placed a grid with cells of size 1km x 1km over the map of Antipolo, and designated the center of each cell as a candidate site. We chose this granularity because of limitations in computational resources. Then, we used the Google Roads API to identify sites near existing roads. Only sites for which road segments were found by the API were kept.

We proposed two optimization metrics for policy makers to consider when selecting a goal to optimize for, and two demand adjustment methods which allow policy makers to adjust the weight given to populations that already have access to existing health facilities. The first metric considers the number of people living within a 30-min drive of a facility, which is the goal stipulated in the Philippine Health Facility Development Plan (PHFDP) [S1 File]. The second metric accounts for the number of expected visitors as suggested by [23] (Eq 2).

$$f(t_{i,j})=\frac{1}{1+{\left(\frac{t_{i,j}}{\theta }\right)}^{\beta }}$$ (1)

$${Visitors}_{i,j}=\mu P^{\alpha }B^{\sigma }f(t_{i,j})$$ (2)

where *P* is the population at site *i*, *B* is the number of beds at facility *j*, *t*_*i*,*j*_ is the travel time in minutes from site *i* to facility *j*, and μ, α, σ, θ, β are constants.

In their paper, [23] proposed that the attractiveness of a facility is a product of (1) its capacity and (2) people’s willingness to travel to it. We set the capacity of each RHU at 20,000 people as specified in the PHFDP [S1 File]. Eq 1 modeled willingness to travel as a score between 0 and 1, based on experimental results performed in Florida, USA [23].

In this study, we adjusted demand to account for areas which are already “covered” by existing RHUs. In Method A (Zeroed Demand), we located areas within a 30-minute drive of an RHU, then set demand in those areas to 0. In effect, this excluded populations within 30 minutes of existing RHUs from the calculation, giving full priority to people without RHU access. In Method B, we reduced demand around an existing RHU (within a 30-minute drive) based on its capacity (S1 Appendix). This gave priority both to people without RHU access and those in areas where the capacity of existing RHUs could not adequately meet the demand. We compared our findings with results generated by algorithms with no demand readjustment employed. By applying such methods, the algorithms are optimized for areas with existing demand, often located in remote or underserved areas, which would help policy makers address issues of healthcare equity.

We extended the problem to a multiple facility problem, and presented the results for a two-facility optimization. For Metric 1, the code was written to find the total number of people living within a 30 minute drive of either one of the two facilities. For Metric 2, which accounted for the number of visitors, the algorithm was designed to eliminate duplication of demand (S2 Appendix). Once a site was chosen, the demand attracted by that site was added to its coverage score, then subtracted from the population. This also forced the algorithm to optimize for the remaining uncovered populations.

Finally, we validate our results following the procedure performed by [18]. First, we assume that there are no health facilities present, run the facility location model, and compute the selected optimization metric. Then, we compute the optimization metric based on the locations of the current RHUs. The expectation is that the locations selected by the algorithm perform at least as well as the current RHU system in terms of the selected metrics. We note that optimization metrics are merely one part of a multi-faceted decision process, and the optimality of the selected locations depends on multiple factors identified by local governments.

## Results

**Fig 2:**
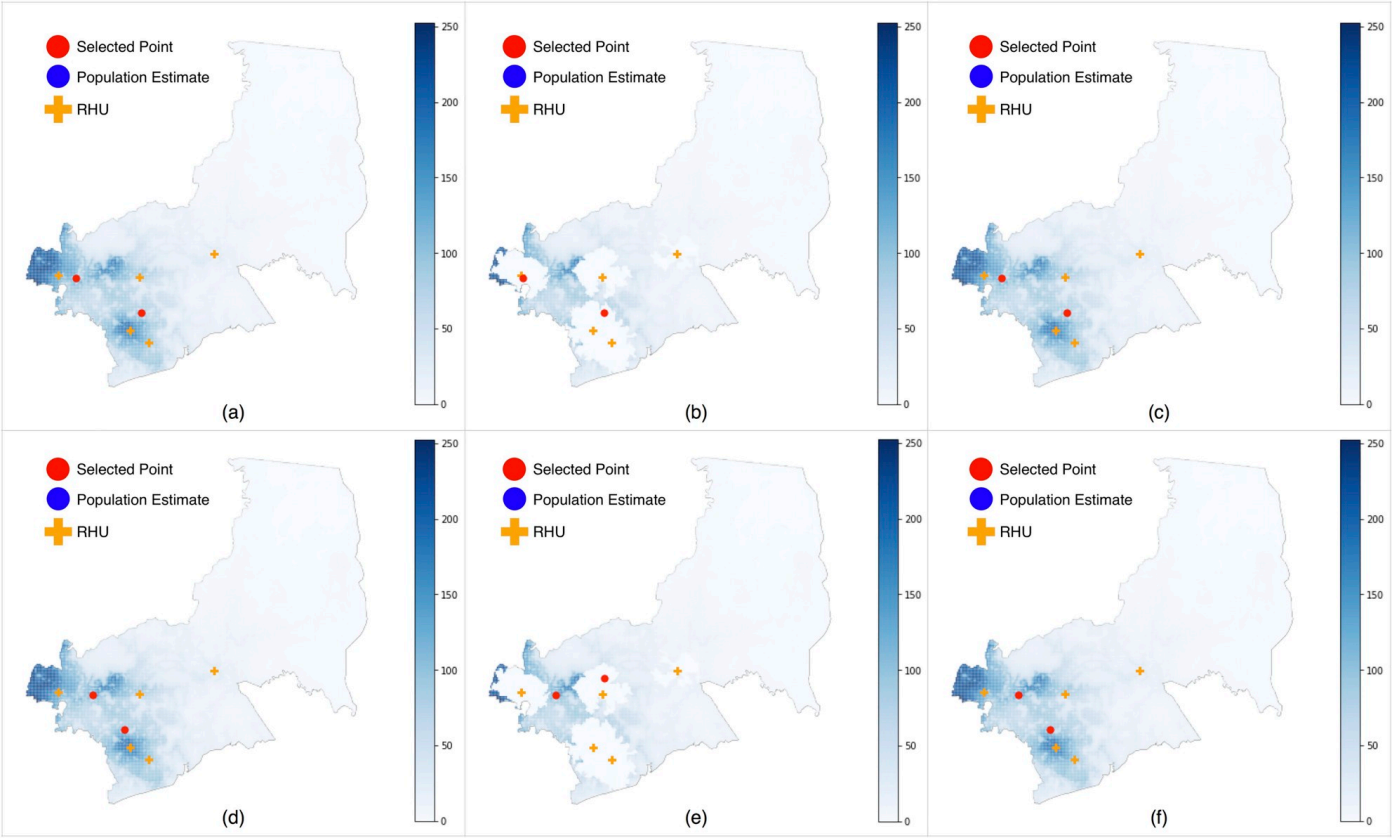
Optimized locations for 1 RHU in Antipolo City. (a) Metric 1, No demand adjustment, Buliran Rd, Brgy. San Luis (Near Philippine Intl. College), (b) Metric 1, Method A, Sumulong Hwy, Brgy. Mambugan, (Near Mambugan Brgy. Hall), (c) Metric 1, Method B, Magsaysay Ave, Brgy. Dela Paz, (Near Robinsons Place Antipolo), (d) Metric 2, No demand adjustment, Sumulong Hwy, Brgy. Santa Cruz, (Near Town and Country Estates), (e) Metric 1, Method A, Sumulong Hwy, Brgy. Santa Cruz, (Near Town and Country Estates), (f) Metric 1, Method B, Sumulong Hwy, Brgy. Santa Cruz, (Near Town and Country Estates).

The results illustrated the strengths of each method and the associated tradeoffs. We baselined the results with simulations using unadjusted demand (Fig 2A and 2D).

Results using Metric 1 selected sites located directly in or adjacent to high population centers. The variations using no demand adjustment and Method B (Fig 2A and 2C) chose sites in the southeast part of Antipolo City (Brgy. San Luis/Dela Paz), while the one using Method A (Fig 2B) chose a site in Antipolo’s western side (Brgy. Mambugan). Compared to Metric 1, Metric 2 chose sites further away from individual population centers, and closer to the geometric center of Antipolo’s urban area (Fig 2D–2F), despite these areas being less populated. These results aligned with our intuitive understanding of the algorithms. Metric 1 was concerned with the population within 30-minute travel times, and thus selected localized high population sites. Metric 2 maximized visitorship from the entire city, and thereby chose more central locations.

We expected simulations using Method A (Zeroed demand) to select sites that were farther from existing RHUs, and Method B (Excess demand) to choose locations where existing demand was greatest, regardless of whether these sites were close to existing RHUs or not. Interestingly, both Methods A and B put facilities close to existing RHUs. This indicates that in Antipolo City, (1) highly populated areas either currently have or are located close to RHUs, but (2) these RHUs are likely inadequate to meet the demand in those areas. This scenario provided a second interpretation of the results. Instead of building new RHUs at the locations which the algorithm selected, local governments may consider expanding current facilities at the chosen sites to cater to existing or unserviced demand in the identified areas.

**Fig 3:**
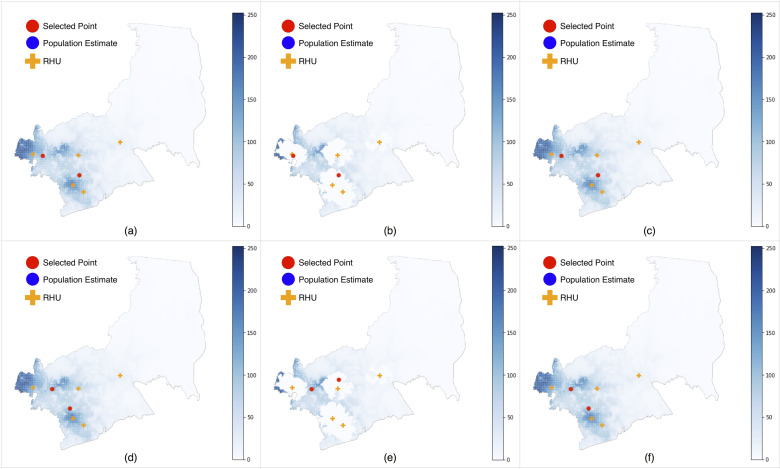
Optimized locations for 1 RHU in Antipolo City. (a) Metric 1, No demand adjustment, Sumulong Hwy, Brgy. Mambugan (Near Mambugan Brgy. Hall) & Buliran Rd, Brgy. San Luis (Near Philippine International College), (b) Metric 1, Method A, Sumulong Hwy, Brgy. Mambugan (Near Villa Cecilia Subdivision) & Buliran Rd, Brgy. San Luis, (Near Philippine International College), (c) Metric 1, Method B, Sumulong Hwy, Brgy. Mambugan (Near Mambugan Brgy. Hall) & Buliran Rd, Brgy. San Luis (Near Philippine International College), (d) Metric 2, No demand adjustment, Sumulong Hwy, Brgy. Santa Cruz (Near Town and Country Estates) & Magsaysay Ave, Brgy. Dela Paz (Near Robinsons Place Antipolo), (e) Metric 1, Method A, Sumulong Hwy, Brgy. Santa Cruz (Near Town and Country Estates) & Sun Valley W Drive, Brgy. Inarawan (Near Forest Hills Golf & Country Club), (f) Metric 1, Method B, Sumulong Hwy, Brgy. Santa Cruz (Near Town and Country Estates) & Magsaysay Ave, Brgy. Dela Paz (Near Robinsons Place Antipolo).

In the two-facility scenario, we found that the behavior of the metrics and methods were similar to that of the one-facility scenario (Fig 3). Given that we increased the number of facilities, we expected Metric 2 to place one of the facilities closer to the center of the rural areas to attract visitors in that area. Interestingly, the results still chose two urban locations– which we can attribute to the parameters used in Eq 2.

In particular, the parameter θ, which we interpret as the travel time at which people are only half as willing to travel to a healthcare facility, was estimated to be θ = 6.29 minutes [23]. This will likely differ in the Philippines, where people in rural areas are accustomed to travelling for hours, or even days, to reach the closest health facility [26]. This implied the need to tune the parameters of the model and replicate the experiments of [23] in the Philippines, in order to yield more appropriate values.

**Fig 4:**
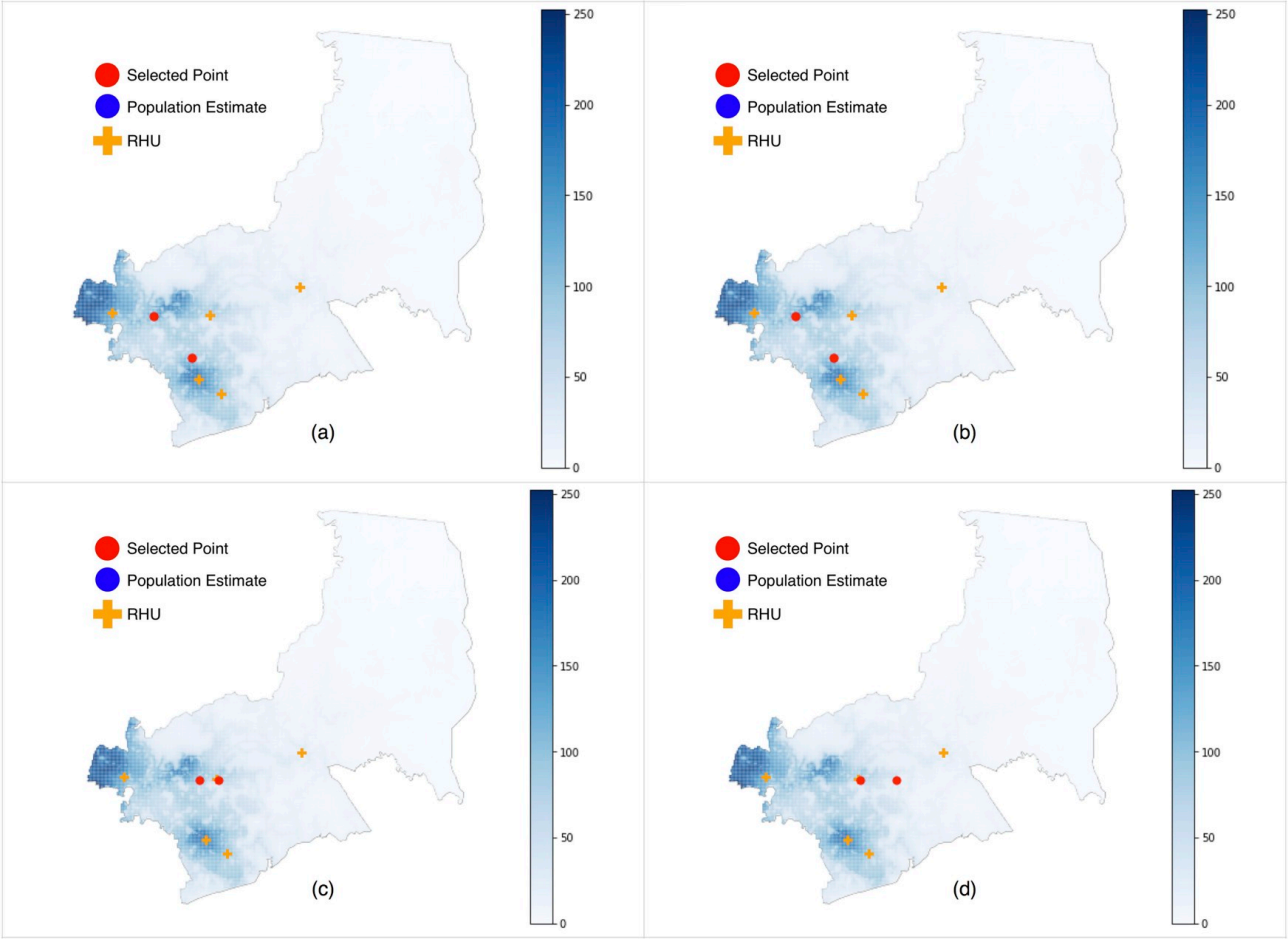
Simulations with modified distance decay formula parameters. (a) θ = 6.29 minutes, (b) θ = 45.0 minutes, (c) θ = 60.0 minutes, (d) θ = 120.0 minutes, All simulations run with no demand readjustment, *B* = 20, μ = 0.20, α = 0.66, σ = 0.40, β = 2.14, as estimated by [23].

We simulated results by adjusting θ in Eq 1 using the parameters estimated by [23] as a baseline (Fig 4A) and found that increasing θ causes the selected RHU sites to move away from individual population centers, and towards central or rural areas. This reflects the likelihood that people are more willing to travel farther to reach these central areas, and that central areas allow RHUs to expand their coverage. However, a consequence of this adjustment was that selected sites were now further away from residents living at the eastern side of the city, increasing travel costs for patients coming from geographically isolated and disadvantaged areas (GIDA). This raises issues of equity in healthcare resource allocation.

We explored the possible effects of each method on equity and presented them in Table 3.

**Table 3:** Comparison of tradeoffs by method.

Method	Trade Offs
1-A	**Pro:** Biased in favor of populated, geographically isolated and disadvantaged areas (GIDA) without RHUs.
	**Con:** Optimizes for densely populated remote sites at the expense of others; May choose "inefficient" sites since the algorithm does not account for actual burden of disease and historical utilization of primary care services.
2-A	**Pro:** Biased in favor of urbanized locations where demand is greatest; May select areas with existing RHUs, in which case the recommendation is to upgrade the existing primary care facilities.
	**Con:** Optimizes for single sites at the expense of others; Often selects urban areas, wherein available land may be more difficult to find.
1-B	**Pro:** Optimizes for multiple population centers, thus minimizing travel cost with respect to the whole city; Biased in favor of GIDA.
	**Con:** Has lesser preference for GIDA compared to method 1-A.
2-B	**Pro:** Optimizes for multiple population centers, thus minimizing travel cost with respect to the whole city; Biased in favor of where demand is greatest.
	**Con:** Least likely to select rural areas and GIDA since it prioritizes urban centers.

Finally, we validated that the algorithms that compute both metrics 1 and 2 achieve the expected performance, and present the results in Table 4.

**Table 4:** Results of algorithm validation.

Metric	Current RHU System	Algorithm
Metric 1. Population Covered within 30 Minutes	310,971	729,502
Metric 2. Patients Attracted	5,394	11,133

**Fig 5:**
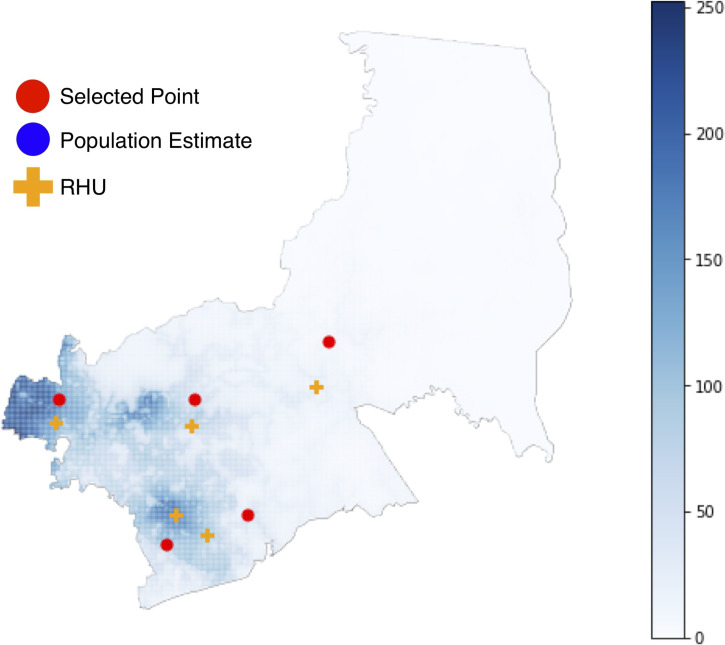
Optimal facility locations for 5 RHUs using Metric 1.

**Fig 6:**
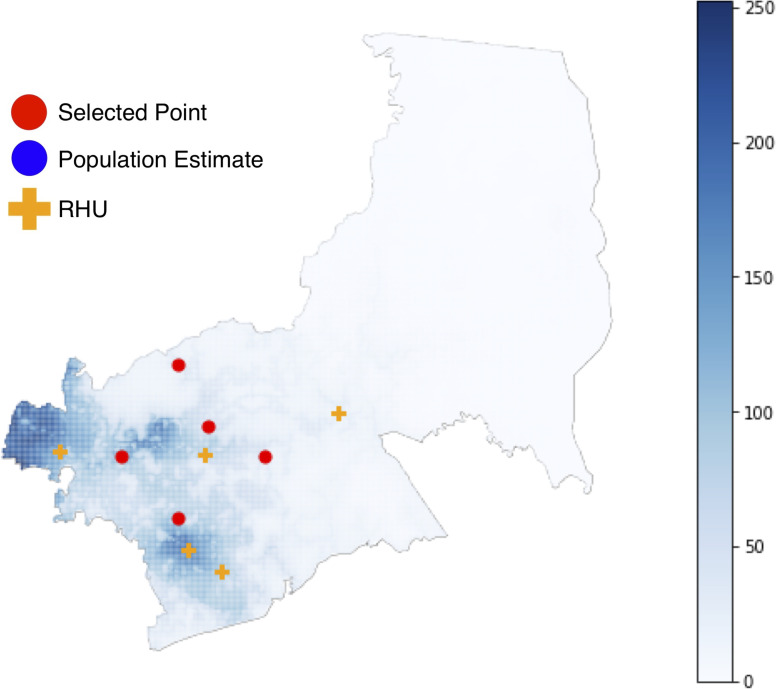
Optimal facility locations for 5 RHUs using Metric 2.

We find that the algorithm achieves better performance across both metrics, which confirms that the algorithms work as expected. The locations selected for the five RHUs for metrics 1 and 2 are shown below (Figs 5 and 6).

## Conclusion

Determining where to build a health facility is critical in ensuring equitable and efficient access to healthcare services. In this paper, we proposed a framework for selecting an optimal location for RHU site selection, by leveraging open source data and empirical work from previous healthcare studies. The choice of metric posed a tradeoff between optimizing for one localized population center (Metric 1) versus multiple population centers (Metric 2), while the choice of demand readjustment depended on which one weighs more in decision-making: prioritizing populations without RHU access (Method A), or including populations in areas where RHUs were insufficient to meet demand (Method B). Results that placed RHUs close to existing facilities also opened the possibility of expanding current RHUs instead of building new ones. These results differed based on the number of facilities to be constructed or upgraded. Ultimately, policy makers must weigh the issues of equity when deciding which outcomes to optimize for.

While our study proposed a framework to objectively identify the ‘best’ location where to build a health facility from an economic optimization perspective, it is still necessary for policy makers to develop a multi-criterion methodology or tool in health facility location decisions especially in low and middle-income countries (LMICs). Health facility decision-making is complex and may require different criteria, including cultural and socio-economic realities. Therefore, optimization models may be used to identify desirable candidate sites, but the final decision still requires multi-objective decision making tools [5, 27].

There were four main limitations of the study. First, the framework did not identify available land for RHU construction. This can be addressed by collecting ground level data, or by using satellite imagery to classify these areas [28–30]. Second, the parameters in the distance decay model were not tuned to the local setting. Moreover [23], modeled the number of visitors using patient discharges in hospitals, not primary care facilities. These support the need for future work to replicate such studies in the local setting using the desired metrics. Third, the focus of the paper on RHUs merits the incorporation of private clinics into the analysis as they provide similar services [31]. As of this paper’s writing, there was no readily available dataset for private clinics, which indicated the need for further data collection. Finally, the paper only studied healthcare resource allocation through constructing RHUs–alternatives like building roads and improving transportation networks also affect healthcare access, which merit further study.

For future studies, researchers could extend our framework by examining the optimal location of primary care facilities, hospitals and other ancillary health facilities (e.g. stand-alone laboratories, pharmacies) in a given location. A well-functioning health system should have a robust referral network system, and the optimal distance among different types of health facilities is a critical factor in the efficient and equitable delivery of healthcare services.

## Supporting information

S1 Appendix Algorithm 1: Computing expected demand. (PDF)

S2 Appendix Algorithm 2: Computing number of visitors without double counting for a single configuration. (PDF)

S1 File Philippine Health Facility Development Plan 2020–2040. (PDF)
