# Supplementary material for: Optimizing health facility location for universal health care: A case study from the Philippines
Source: PLoS One. 2021 Sep 9;16(9):e0256821. doi: 10.1371/journal.pone.0256821 (PMC8428763; doi:10.1371/journal.pone.0256821)
Supplement: S1 Appendix — (PDF) [file pone.0256821.s001.pdf]

S1 Appendix. Algorithm 1: Computing Expected Demand.

Input:

(1) *faci*: Health facility dataset,

(2) *pop*: Population dataset

Algorithm:

1. Shuffle the rows of *pop*
2. For *population_point* in *pop*:
   a. *nearby_faci* ← Subset *faci* to facilities inside *population_point.isochrone_30_min*
   b. *total_capacity* ← Sum the capacities of the facilities in *nearby_faci*
   c. If *total_capacity* ≥ *population_point.population*:
      i. For each *facility* in *nearby_faci*:
         1. *expected_visitors* ← *population_point.population* * *facility.capacity/total_capacity*
         2. *remaining_capacity* ← *facility.capacity* - *expected_visitors*
         3. Update the capacity in the *faci* dataset as *remaining_capacity*
      ii. *population_point.population* ← 0
   d. Else:
      i. *population_point.population* ← *population_point.population* - *total_capacity*
      ii. For each facility in *nearby_faci*:
         1. Set the capacity of the corresponding facility in *faci* to 0

Output: Updated *pop* dataset
