# Supplementary material for: Optimizing health facility location for universal health care: A case study from the Philippines
Source: PLoS One. 2021 Sep 9;16(9):e0256821. doi: 10.1371/journal.pone.0256821 (PMC8428763; doi:10.1371/journal.pone.0256821)
Supplement: S2 Appendix — (PDF) [file pone.0256821.s002.pdf]

S2 Appendix. Algorithm 2: Computing Number of Visitors without Double Counting for a Single Configuration*

***Note:**

A configuration defines which subset of IDs from the set of all candidate sites should be optimized (e.g. selecting 2 RHU sites for upgrading).

Input:

(1) *site_list*: List of site IDs based on the defined configuration,

(2) *pop*: List of population values at each population point for the entire city,

(3) *exp_visitors*: Matrix of expected visitors (of size: n_population_points x n_facilities, eq 2). We use this as a reference

Algorithm:

1. *total_visitors_count* ← 0
2. For *site_id* in *site_list*:
   a. *actual_demand* ← List of the number of visitors from each population point that may travel to the site (site_id)to the site corresponding to *site_id*, Calculated as the minimum value between the remaining population (*pop*) and the expected number of visitors from the population point (*exp_visitors* at *site_id*)
   b. *pop* ← Reduce the remaining population at each population point (*pop*) by the *actual_demand* at *site_id*
   c. *total_visitors_count* ← Increment the number of visitors for this configuration (*total_visitors_count*) by the number of visitors contributed by *site_id* (sum(*actual_demand*))

Output: Total expected visitors *total_visitors_count* for the defined configuration
