# Supplementary material for: Optimizing health facility location for universal health care: A case study from the Philippines
Source: PLoS One. 2021 Sep 9;16(9):e0256821. doi: 10.1371/journal.pone.0256821 (PMC8428763; doi:10.1371/journal.pone.0256821)
Supplement: S1 File — (PDF) [file pone.0256821.s003.pdf]

PHILIPPINE HEALTH FACILITY DEVELOPMENT PLAN 2020-2040

Investing in resilient and sustainable health facilities towards Universal Health Care

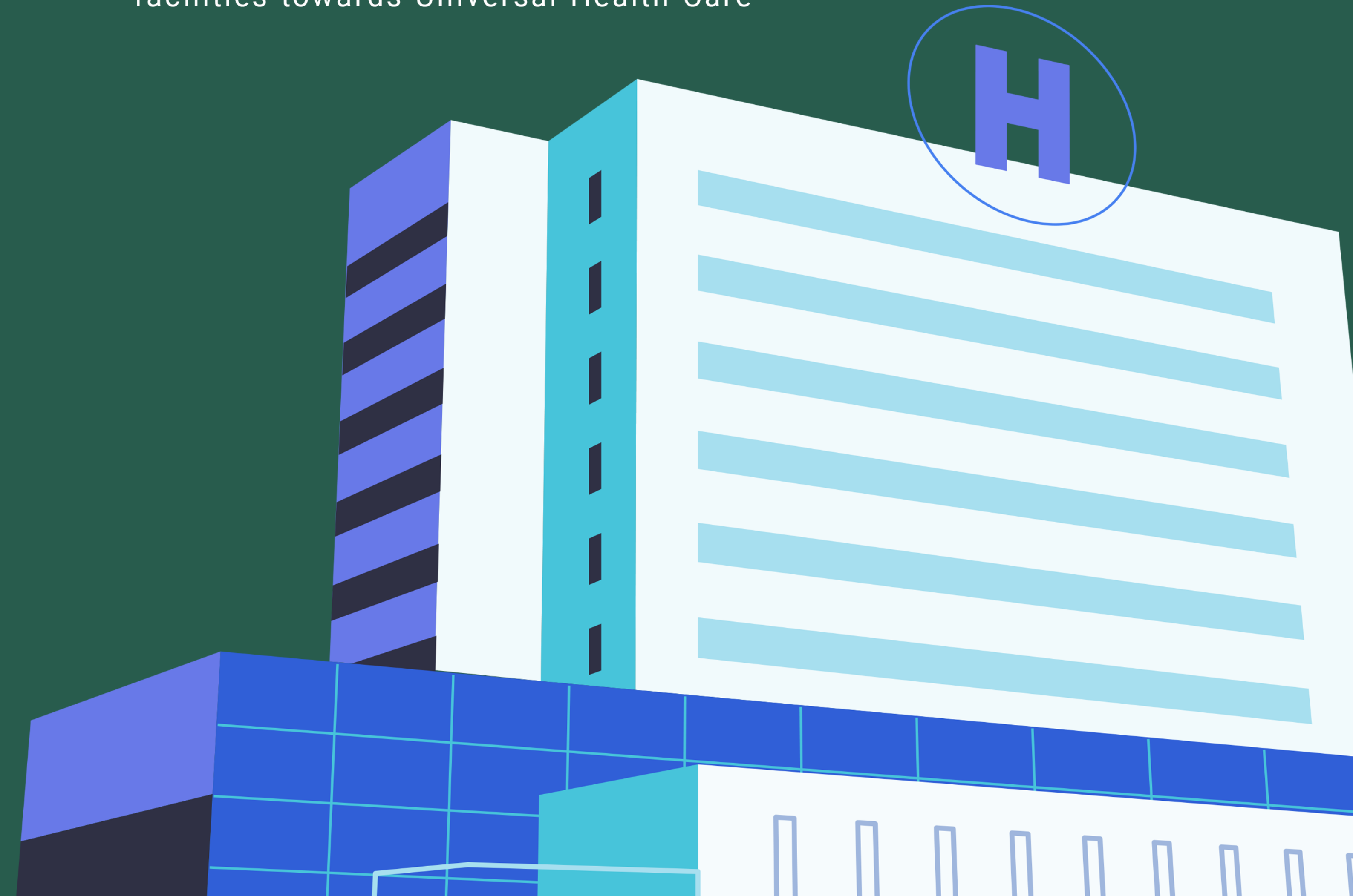

Philippine Health Facility Development Plan 2020-2040

© Department of Health 2020

Published by

Health Facility Development Bureau
Department of Health
San Lazaro Compound
Rizal Avenue, Sta. Cruz
Manila 1003, Philippines

The mention of specific companies or of certain products does not imply a preferential endorsement or recommendation by the Department. This report may be reproduced in full or in part for non-profit purposes without prior permission, provided proper attribution to the Department is made.

An electronic copy of this publication can be downloaded at:
bit.ly/PHFDP2020_2040.

Suggested Citation: Department of Health. (2020). Philippine Health Facility Development Plan 2020-2040. Manila, Philippines: Department of Health.

SUMMARY

The **Philippine Health Facility Development Plan (PHFDP) 2020-2040** articulates the required investments for health facilities.

The **PHFDP 2020-2040** uses a needs-based approach, accounting for the future burden of disease in estimating the needed health facilities in the medium to long-term. The Plan follows the service delivery model envisioned in the Universal Health Care Act (2019): a primary care-oriented and integrated health system. Also, it outlines approaches for health facilities to be climate resilient and environmentally sustainable.

The Plan is forward looking. It requires large investments and bold reforms. It aspires for a health system that every Filipino deserves and a middle-class society should have. The future need for health facilities is large. Decision-makers must take path-breaking approaches to build health facilities, especially in areas that need the most.

The realization of the Plan is anchored on two elements: health human resource and sustained financing. The Health Human Resource Master Plan must be aligned with this Plan. Health infrastructure without enough workers is a waste of public resources. Private and public financing are critical in closing the health infrastructure gaps.

Highlights of this Plan:

- By 2040, outpatient visits and inpatient bed-days are expected to increase by 60% mostly due to non-communicable diseases (NCDs).
- To meet the projected need, the Philippines needs large and sustained investments in health facilities. In 2018, the public and private sectors spent about PhP113 billion (0.6% of GDP) on health infrastructure and other capital formation. The government must increase this annual spending by more than two folds on top of private sector spending.
- The national and local governments must spend at least PhP56 billion every year in the medium-term and encourage the private sector to increase and sustain its investments to substantially reduce the large health infrastructure gap.
- With the large public spending required, the way the country finances and governs health facilities must be radically different this time around – a paradigm shift is needed. Capital formation on health should be considered as assets with enormous economic returns. Without sacrificing the goal of equity and universal access, health care provider networks of local governments should consider health infrastructure as investments that have income generating potential.
- Underinvestment in health is large. In 2018, public spending on health was only USD 50 per person compared to USD 100 in ASEAN countries that successfully implemented UHC. The country's health

system remains hospital-centric. Hospital care accounted for 50% of total health spending. Primary care only accounted for 4%.

- The Philippines has 3,900 primary care facilities (PCF), of which 2,600 are Rural Health Units/ Health Centers (RHU/ HC). Only 50% of Filipinos have access to an RHU/ HC within 30 minutes of travel time. The country needs an additional 2,400 RHU/ HCs by 2025.
- A total of 60,000 primary care physicians are needed to meet the current needs for primary care assuming staffing requirements are based on physicians. The projected need for primary care physicians (PCP) is equivalent to the current stock of available physicians, generalists, and specialists combined in the country. Universal primary care may only be realized if bold reforms are pursued such as task shifting and rapid increase in production capacity for the health workforce.
- The country has 105,000 hospital beds with a bed density similar to the poorest countries in the world (1.2 per 1,000 population). Upper middle-income and high-income countries, which the Philippines is projected to become by 2021 and aspires to be by 2040 have bed density of 4 per 1,000 population, on average. An additional 400,000 beds are needed, majority of which are Level 1 beds, to meet the projected hospital care by 2040 (around 2.7 beds per 1,000 population). The Plan includes the potential role of public and private sectors in closing the total gap.
- This Plan includes a framework for allocation of national government resources. The framework is anchored on equity. Provinces and HUCs with low capacity as measured by public spending per capita, poverty incidence and presence of GIDA are more likely to have higher national government subsidy.
- Investments for government-run special health facilities are critical to complement primary care and hospital-based general health services.
- Specialty centers for sixteen (16) specialties shall be established: cancer care, cardiovascular care, lung care, renal care and kidney transplant, brain and spine care, trauma care, burn care, orthopedic care, physical rehabilitation medicine, infectious disease and tropical medicine, toxicology, mental health, geriatric care, neonatal care, dermatology care, and eye care.
    - Selected DOH hospitals shall be designated as National Specialty Centers, Advanced Comprehensive Specialty Centers and Basic Comprehensive Specialty Centers for each of the specialties. Investment needs for infrastructure and equipment until 2025 totals PhP82 billion.
    - Upgrading and establishment of Specialized Laboratories including the National, Sub-national and Regional Reference Laboratories shall require a total of PhP1.1 billion.
    - Capital investment requirements for Blood Service Facilities and Drug Abuse Treatment and Rehabilitation Facilities in the national, subnational and regional levels total Php 3.5 billion and PhP8.4 billion, respectively.

ACKNOWLEDGEMENT

The Philippine Health Facility Development Plan 2020-2040 was developed by the Department of Health (DOH) under the technical guidance and supervision of Undersecretary Lilibeth C. David and Director Ma. Theresa G. Vera. The Asian Development Bank (ADB) provided technical and financial support through the Urban Climate Change Resilient Trust (UCCRTF).

This publication was written by the following DOH staff and ADB consultants: Valerie Gilbert Ulep (Team Leader/Economist, ADB & PIDS), Jhanna Uy (PIDS), Health Facilities and Infrastructure Development Team (HFIDT), and Health Facility Development Bureau – Health Systems Management and Support Division (HFDB-HSDMSD).

The following contributed to specific sections of the Plan: Jerson Taguibao (Ateneo School of Medicine), Melvin Marzan (ICT Specialist, ADB), Frank Rameloo (Infrastructure Specialist, ADB), Ramon San Pascual (Climate Change Specialist, ADB and HCWH), Danielle Dalisay (Geospatial Specialist, ADB), Jonathan Flavier (LGU specialist), LJ Reyes (Yale). The list of contributors and technical working group members are in Annex H.

This publication benefitted from the comments and inputs of Eduardo P. Banzon (ADB), Rikard Elfving (ADB), Sakiko Tanaka (ADB), Jose Tiusonco (ADB), Ramon Abrosca (ADB), and Joy Amor Bailey (ADB).

URBAN CLIMATE CHANGE RESILIENCE TRUST FUND
Asian Development Bank

The ROCKEFELLER FOUNDATION

Schweizerische Eidgenossenschaft
Confédération suisse
Confederazione Svizzera
Confederaziun svizra

Swiss Confederation

Federal Department of Economic Affairs, Education and Research EAER
State Secretariat for Economic Affairs SECO

UKaid
from the British people

TABLE OF CONTENTS

SUMMARY	i
ACKNOWLEDGEMENT	iii
TABLE OF CONTENTS	iv
LIST OF FIGURES	vi
LIST OF TABLES	viii
ANNEX	x
ACRONYM	xi
INTRODUCTION	xiv
CHAPTER I. The Philippine Healthcare System	3
A. Health Service Delivery	5
B. Health Financing	12
C. Human Resources	17
D. Health information Systems and Communication technology	18
E. Governance	20
F. Macro-trends that will Influence demand for healthcare	21
CHAPTER II. Vision: A Modern, Resilient, and Sustainable Healthcare System	26
CHAPTER III. Approach in Estimating the Need for Health Facilities	34
A. Development of Resource Stratified Frameworks (RSF)	35
B. Projecting the Burden of Disease	36
C. Development of Disease Models and Probability Trees	37

D. Estimation of Need for Health Facilities and Equipment 37

CHAPTER IV. The Need for Health Facilities 39

A. The Need for Health Stations and Primary Care Facilities 41

B. The Need for Hospital Care 43

C. The Need for Medical Equipment 46

CHAPTER V. Allocation Framework 50

The cost to reduce environmental risk and improve resilience in health facilities 57

CHAPTER VI. Localization and Operationalization 62

CHAPTER VII. Special Facilities 71

A. Specialty Centers 72

B. Specialized Laboratories 76

C. Blood Service Facilities 78

D. Drug Abuse Treatment and Rehabilitation Facilities 82

E. Military Health Facilities 87

F. Hospitals of State Universities and Colleges (SUC-Hospitals) 90

CHAPTER VIII. Geolocating and Mapping Health Facilities 92

A. Baselining geospatial coordinates for each health facility 94

B. Mapping health facilities 95

C. Accessibility Analysis for Hospitals and Rural Health Units 96

CHAPTER IX. Monitoring and Evaluation Plan 99

APPENDIX 104

LIST OF FIGURES

Figure 1. Infant mortality and Gross National Income, 2018	4
Figure 2. Universal Healthcare Coverage Index, 2017	5
Figure 3. BHS and barangay ratio, and poverty incidence, 2018	6
Figure 4. Distribution of populations without access to a Rural Health Unit/ Health Center within 30 minutes	7
Figure 5. Bed to population ratio in ASEAN and selected geographical locations, latest available year	8
Figure 6. Number of hospital beds, Philippines, 1990-2018	8
Figure 7. Level 1 hospital beds and poverty incidence, 2018	9
Figure 8. Level 2 hospital beds and poverty incidence, 2018	9
Figure 9. Distribution of Level 3 hospital beds by region, 2018	10
Figure 10. Number of CT scan machines per million population, by ASEAN countries, 2019	11
Figure 11. Number MRI machines per million population, by ASEAN countries, 2019	11
Figure 12. Number of X-ray machines, 2019	11
Figure 13. Number of CT scan machines per million population, by region, 2019	12
Figure 14. Number of MRI machines per million population, by region, 2019	12
Figure 15. Government spending on health in comparison to other ASEAN countries, 2018	13
Figure 16. Health spending by health provider, 2019	13
Figure 17. Estimated spending on primary care, 2018 or 2019	14
Figure 18. Spending on capital formations on health, 2016-2018	14
Figure 19. Public spending for health per capita, 2018	15
Figure 20. DOH budget (general appropriations) and sin tax allocation to health, 1990-2018 (in billions 2018 prices)	15
Figure 21. Number of HFEP projects, 2008-2019	16
Figure 22. Absorptive capacity of HFEP, 2008-2019	16
Figure 23. Median HFEP total disbursement by poverty incidence, 2008-2019	17

Figure 24. Availability of physician and poverty incidence, 2018 17
Figure 25. Availability of health workers in Rural Health Units, 2019 18
Figure 26. Governance structure of PHL healthcare system 20
Figure 27. Burden of Disease in the Philippines, 1990-2017 22
Figure 28. Real GDP growth, 2000-2021 23
Figure 29. Typhoon and flood disasters in the Philippines 23
Figure 30. World Risk Index, 2020 24
Figure 31. Availability of power generator, electricity, and sanitation facilities in RHUs 25
Figure 32. Patient flow under UHC 28
Figure 33. Climate-resilient health system and building blocks of the health system 30
Figure 34. Building climate-resilient and environmentally sustainable health care facilities 31
Figure 35. Steps in estimating need for health facilities 35
Figure 36. Resource Stratified Framework - Defining services across the Continuum of Care 35
Figure 37. Projected bed-days for all hospital levels, by disease category 40
Figure 38. Projected outpatient primary care visits, by disease category 40
Figure 39. Bed-to-population ratio (per 1000), selected comparator countries 44
Figure 40. National Allocation Framework 52
Figure 41. Steps to Localize the PHFDP 63
Figure 42. Process for Creating the Development Plans for Specialty Centers 74
Figure 43. Health Facilities and their Catchment Population 75
Figure 44. Blood Service Network 80
Figure 45. Health Facility Overview Map of Maguindanao 95
Figure 46. Sample of 60-minute isochrone around a health facility in Rizal 97
Figure 47. Kalinga Hospital Access Map 98

LIST OF TABLES

Table 1. Number of core health facilities, Philippines, 2019	6
Table 2. Roles of health facilities in UHC	29
Table 3. List of the 25 causes of morbidity and mortality, Philippines, 2017	36
Table 4. Assumptions in estimating the need for health facilities	37
Table 5. Projected number of primary care consultations, inpatient visits, and equipment use	41
Table 6.Number of barangay health stations and barangays	41
Table 7. Supply and need for primary care facilities	42
Table 8. Need for outpatient primary care physicians	43
Table 9. Supply and need for Level 1 hospital beds	44
Table 10. Supply and need for Level 2 hospital beds	45
Table 11. Supply and need for Level 3/ Apex hospital beds	46
Table 12. Supply and need for X-ray machines	47
Table 13. Supply and need for CT scan machines	47
Table 14. Supply and need for MRI machines	48
Table 15. Supply and need for LINAC	49
Table 16. Estimated cumulative gap in hospital beds until 2040, by public and private	51
Table 17. Capacity vs. Gap in Level 3 hospital beds and equipment	53
Table 18. Public investments by health facility, 2021-2025	54
Table 19. Gap and priority facility, by province and HUC	54
Table 20. Prioritization criterion among HPCN facilities	57
Table 21. Estimated cost of health facilities (in 2020 prices)	58
Table 22.Factors for environmental risk measures	59
Table 23.Factors for resilient measures	59
Table 24. Return of investment (ROI) of resilient health infrastructure	61
Table 25. Sources of financing	68

Table 26. Laws and Mandates relevant to Specialty Care Services 73
Table 27. Different types of Specialty Centers 74
Table 28.Costing for Specialty Centers (in millions Php) 76
Table 29. Summary of Service Capability for Laboratories 77
Table 30. Facilities in the Specialized Laboratory Network 77
Table 31. Cost Estimate for Upgrading the Specialized Laboratory Network (in millions) 78
Table 32.Current Classification of Blood Service Facilities 80
Table 33. Blood Centers in the Philippines in the context of Universal Health Care (UHC) Act 81
Table 34. Development Plan for DOH Blood Centers with Estimated Total Cost 82
Table 35. Summary of Service Capabilities of Drug Abuse Treatment and Rehabilitation Facilities 84
Table 36. Results Matrix for select Drug Abuse Treatment and Rehabilitation Facilities 85
Table 37. Development Plan for Drug Abuse Treatment and Rehabilitation Facilities 85
Table 38. Cost Estimate of Infrastructure and Equipment for Drug Abuse Treatment and Rehabilitation Facilities (in millions Php) 87
Table 39. Summary of different types of Military Health Service Facilities 88
Table 40. Military Treatment Facilities and Veterans Hospital 89
Table 41. Estimated Cost of Upgrading of V. Luna Medical Center for 2021 (in millions PhP) 90
Table 42. SUC-Hospitals in the count 90
Table 43. Nationwide Coordinate Certainties for Selected Facility Types 93
Table 44. Nationwide coordinate certainties for all facility types 94
Table 45. Coordinate certainties for main health facilities in Maguind 96
Table 46. Barangay and population with a BHS in Maguindanao 105 96
Table 47. Number and facility to population ratio for RHU in 96
Table 48. Monitoring and Evaluation Framework 100
Table 49. Input/Process Indicators 100
Table 50. Output Indicators 101
Table 51. Intermediate Outcome Indicators 103

ANNEX

Annex A: Local Government Unit (LGU) Profiles

Annex B: Access Maps

Annex C: Resource-Stratified Frameworks

Annex D: Designated National Specialty Centers, Advanced Comprehensive Centers, and Basic Comprehensive Centers, Target Year of Establishment, and Estimate Costs for Each Specialty

Annex E: Resource Stratified Framework for Blood Service Facilities (BSF)

Annex F: Development Plan for Blood Centers

Annex G: Resource Stratified Framework for Drug Abuse Treatment and Rehabilitation Facilities

Annex H: Contributors and Members of Technical Working Groups

ACRONYM

**ACC**	Advanced Comprehensive Center
**AFP**	Armed Forces of the Philippines
**AFPHS**	Armed Forces of the Philippines Health Service
**AO**	Administrative Order
**ASEAN**	Association of Southeast Asian Nations
**BARMM**	Bangsamoro Autonomous Region in Muslim Mindanao
**BC**	Blood Centers
**BCC**	Basic Comprehensive Center
**BEMONC**	Basic Emergency Obstetrical and Neonatal Care
**BHS**	Barangay Health Station
**BSF**	Blood Services Facilities
**CALABARZON**	Cavite Laguna Batangas Rizal Quezon
**CAR**	Cordillera Administrative Region
**CBDRP**	Community Based Drug Rehabilitation Program
**CHD**	Center for Health Development
**CT**	Computed tomography
**DALY**	Disability Adjusted Life Years
**DATRC**	Drug Abuse Treatment and Rehabilitation Center
**DOH**	Department of Health
**DRRM-H**	Disaster Risk Reduction and Management in Health
**ECG**	Electrocardiogram
**EMR**	Electronic Medical Record
**GDP**	Gross Domestic Product
**GIDA**	Geographically Isolated and Disadvantaged Areas
**HC**	Health Center (also City Health Center)
**HFEP**	Health Facilities Enhancement Program
**HCPN**	Health care provider network
**HFSRB**	Health Facilities and Services Regulatory Bureau
**HFDB**	Health Facility Development Bureau
**HRH**	Human Resources for Health
**HUC**	Highly Urbanized City
**HVAC**	Heating, ventilation and air-conditioning
**ICC**	Independent Component City
**ICT**	Information Communication Technology
**IHME**	Institute for Health Metrics for Evaluation
**IRA**	Internal Revenue Allotment

**KMITS**	Knowledge Management and Information Technology Service
**LGU**	Local Government Unit
**LINAC**	Linear Accelerator
**MAC**	Medical Administrative Corps
**MC**	Medical Corps
**MIMAROPA**	Mindoro Marinduque Romblon Palawan
**MNCHN**	Maternal, Newborn and Child Health and Nutrition
**MRI**	Magnetic Resonance Imaging
**NBBNetS**	National Blood Bank Network System
**NCD**	Non-Communicable Diseases
**NCR**	National Capital Region
**NHFR**	National Health Facility Registry
**NOH**	National Objectives for Health
**NRL**	National Reference Laboratory
**NSC**	National Specialty Center
**NVBSP**	National Voluntary Blood Services Program
**PBCC**	Philippine Blood Coordinating Council
**PCF**	Primary Care Facility
**PCP**	Primary Care Physician
**PCPN**	Primary Care Provider Network
**PHFDP**	Philippine Health Facility Development Plan
**PhilHealth**	Philippine Health Insurance Corporation
**PNP**	Philippine National Police
**PRC**	Philippine Red Cross
**PSA**	Philippine Statistics Authority
**RHU**	Rural Health Unit
**ROI**	Return of investment
**RSA**	Return Service Agreement
**RSF**	Resource-stratified Framework
**RT-PCR**	Reverse transcription polymerase chain reaction
**SCM**	Service Capability Mapping
**SDG**	Sustainable Development Goals
**SOCCSKSAR-GEN**	South Cotabato, Cotabato, Sultan Kudarat, Sarangani, General Santos
**SRL**	Sub-national Reference Laboratories
**TWG**	Technical Working Group
**UHC**	Universal Health Care
**VC**	Veterans Corps
**WHO**	World Health Organization

INTRODUCTION

The Philippines aspires to be a high-income society by 2040. Central to this vision is a modern health system that provides quality healthcare to all Filipinos. The Universal Health Care (UHC) Act (2019) provides the legal basis of health reforms necessary to realize this vision. Health reforms should be system-wide: a dramatic shift in how the Philippines organizes, finances, and delivers healthcare services.

A critical area for reform is health facilities: address perennial supply gaps in health facilities and priority technology, reduce inefficiencies, and promote resilient and sustainable health infrastructures.

The 2020-2040 Philippine Health Facility Development Plan (PHFDP) is an aspirational plan, which complements the healthcare system envisioned in the UHC Act (2019): primary and integrated care system.

The success of the reform lies in the commitment of national and local implementing agencies, and the support of health providers and the general public. The Plan serves as a guide for decision makers and implementers to ensure their local health infrastructure plans are evidence-based and are aligned with the national goal. The private sector will also benefit from this Plan.

The delivery of UHC should be complemented with broader reforms in health human resource development. While it focuses largely on health facility infrastructures, this Plan ought to complement other medium and long-term development plans for human resources for health (HRH) and information and communications (ICT) technology of the Department of Health (DOH).

The Plan is divided into 9 (nine) chapters:

- **Chapter I** gives a quick assessment of the current Philippine healthcare system;
- **Chapter II** outlines the vision of a resilient and sustainable health system under the UHC Act (2019);
- **Chapter III** describes the approach used in estimating the need for health facilities and medical equipment for the next 20 years;
- **Chapter IV** presents the available health facilities vis-à-vis estimated need for health facilities and medical equipment;
- **Chapter V** outlines the national allocation framework for health facility investments;
- **Chapter VI** presents the operationalization of the health facility development plan;
- **Chapter VII** outlines the national plan for special facilities;
- **Chapter VIII** presents the ongoing effort to geo-locate health facilities; and
- **Chapter IX** describes the monitoring and evaluation plan for the 2020-2040 PHFDP.

CHAPTER I

The Philippine Healthcare System

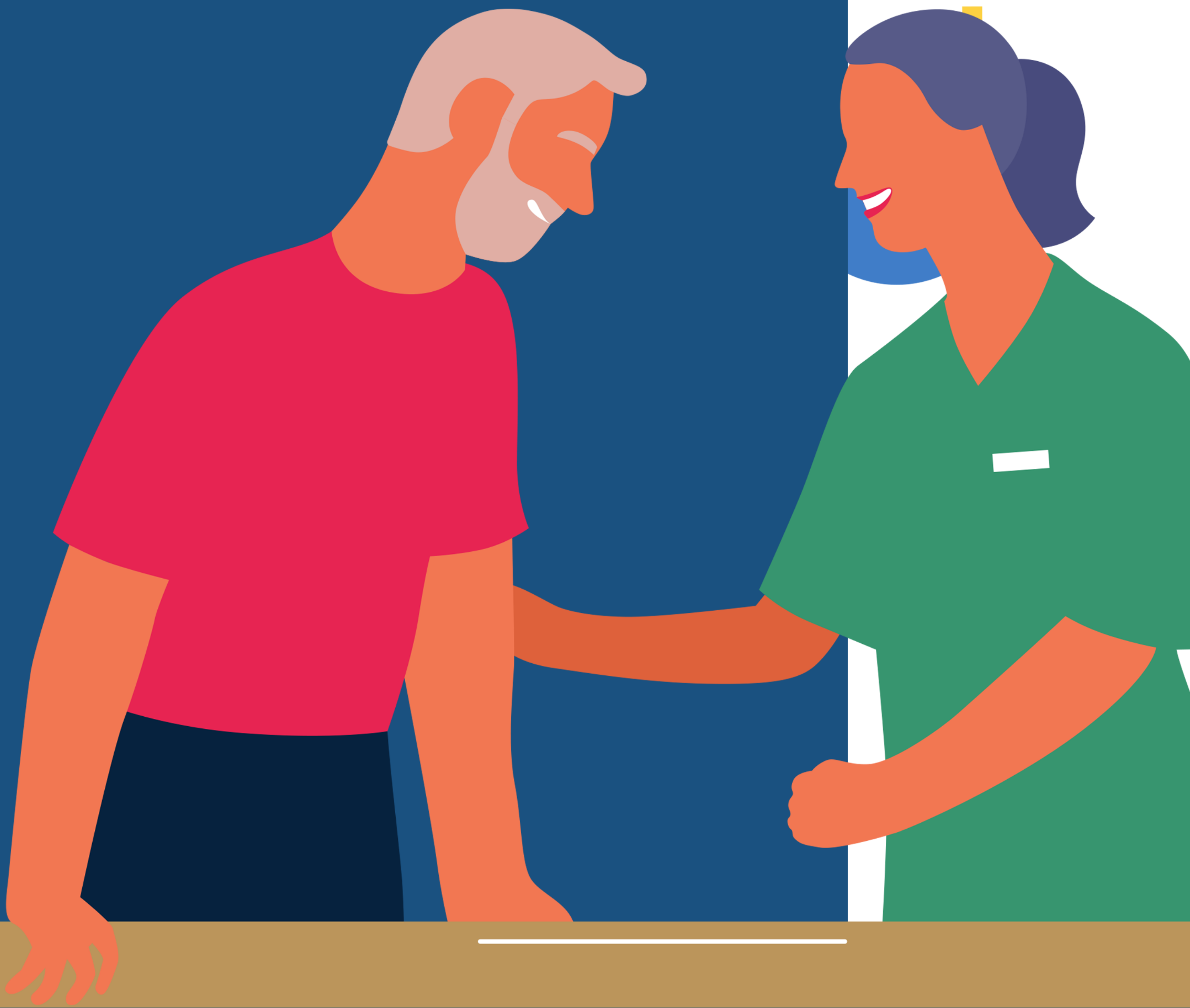

The Philippine population has become healthier in recent decades. The life expectancy at birth has now reached 70 years compared to 55 years five decades ago. More children are surviving before the age of five. In the 1970s, 84 child deaths for every 1,000 live births were recorded every year. At the turn of the century, under-five mortality decreased by almost three-fold (World Bank, 2020).

Despite this progress, the country is lagging on many health outcomes relative to other countries in its income range and large subnational disparities persist. Figure 1 shows the infant mortality rate of different regions relative to ASEAN countries. Relatively wealthier regions like NCR have health outcomes comparable to some upper middle- and high-income countries. In contrast, BARMM is akin to the poorest countries in the world (Philippine Statistics Authority, 2018).

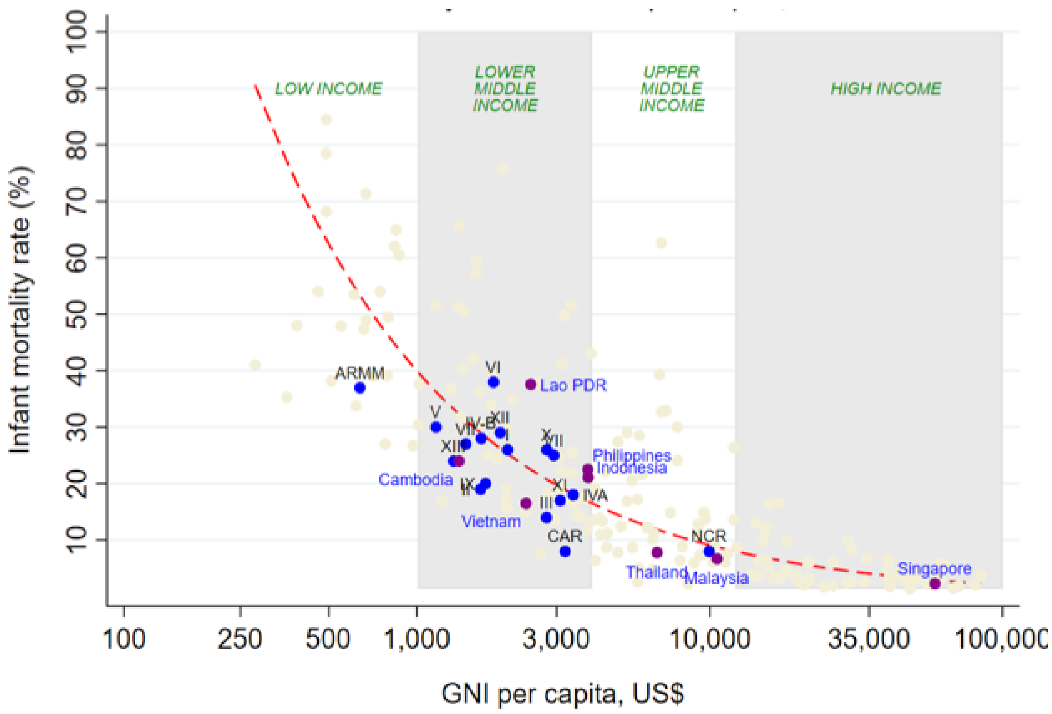

Figure 1. Infant mortality and Gross National Income, 2019

Source: Raw data from Philippine Statistics Authority and World Bank. Note: the black labels and Philippine regions

The slow improvement in health outcomes is a manifestation of longstanding challenges in healthcare access. The Philippines ranks low (a score of 60 out of 100) in the Universal Health Coverage (UHC) Service Coverage Index (Figure 2), an indicator of access to essential services on maternal and child health, infectious diseases, and non-communicable diseases (World Health Organization, 2019). The limited availability of health facilities and health workers, as well as poor financial risk protection remain the top barriers to access. Every year, a million Filipino households are thrust into poverty because of out-of-pocket expenses.

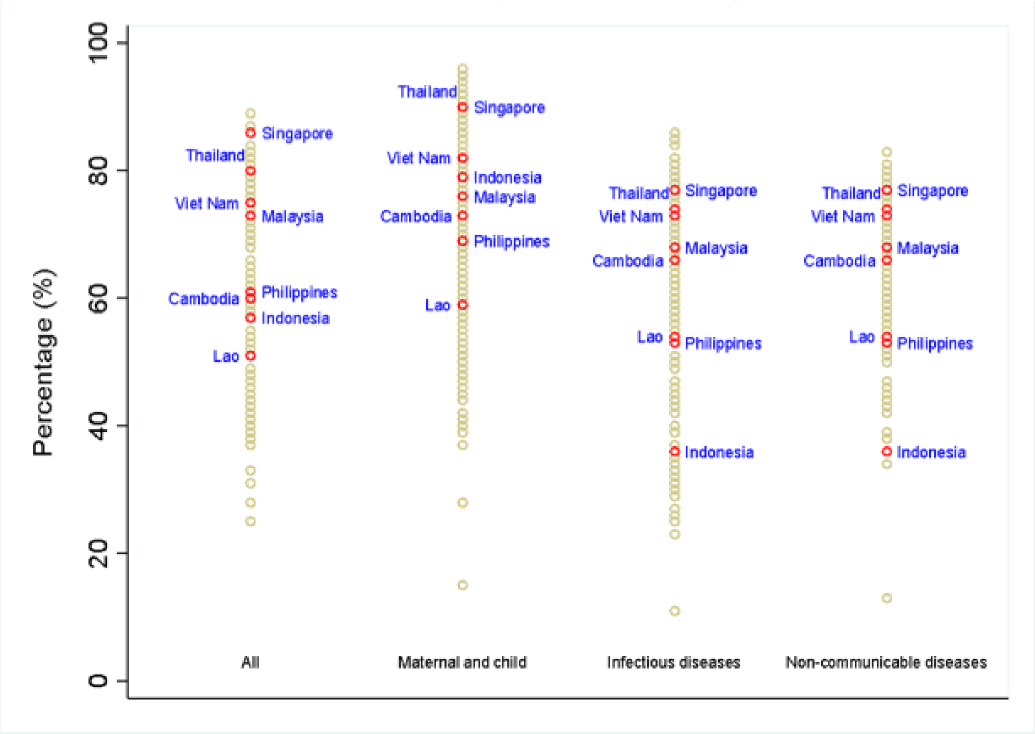

Figure 2. Universal Healthcare Coverage Index, 2017

Source: Raw data from World Health Organization

The low performance on access coverage reflects the current state of the country's health system building blocks: service delivery, health financing, human resources, governance, and information technology.

A. Health service delivery

The health service delivery system is composed of health facilities providing different levels and types of services. The DOH categorizes health facilities into either core or ancillary. Core facilities are health stations, primary care facilities, and hospitals. Ancillary facilities provide support to core facilities. They include diagnostic facilities, specialized facilities, and transition care facilities located within a core facility or as a standalone facility.

In 1991, the Philippines embarked on a major political reform through the Local Government Code. This reform decentralized a number of social services, including health. Administrative and financial control over health facilities, personnel, and governance was transferred from the DOH to the local governments. Governors and mayors finance and manage provincial, district, and municipal hospitals as well as primary care facilities under their jurisdictions. Specialty, regional, and training hospitals were retained under DOH and the national government (Romualdez Jr. AG et al. 2011). Table 1 shows the supply of different health facilities by ownership.

Table 1.Number of core health facilities, Philippines, 2019

Levels	FACILITY					BEDS (count)				
	Private	Publicly-owned			Total	Private	Publicly owned			Total
		National	LGU-owned	Military			National	LGU-owned	Military	
Health stations (e.g., BHS)[1]	No data		22,613		22,613					
Primary Care Facilities (that is Rural Health Units, Health Centers, Private medical clinics)	No data	-	2,593		2,593	-	-		-	
Birthing homes[2]	1,071		835		1,906	625		571		1,196
Infirmaries[2]	336	-	338	9	683	906	-	5,389	-	6,295
L1 hospitals[2]	418	21	297	15	751	14,344	2,750	14,400	909	32,403
L2 hospitals[2]	284	8	33	2	327	26,151	1627	4,285	276	32,339
L3 hospitals[2]	67	41	10	2	120	15,326	20,604	2,692	1,966	40,588

Source: 1 - National Health Facility Registry (NHFR) 2019, 2 - DOH HFSRB Licensed Facilities 2018

Limited frontline health facilities remain a challenge. Health stations, rural health units and health centers (RHU/ HC) are supposedly the entry points of individuals, families, and communities into the health system. In practice, however, patients can go directly to hospitals and other specialized clinics, resulting in large health system inefficiencies.

All barangays should have at least one barangay health station (BHS). While the number of BHS has doubled from 11,000 in 1990 to about 22,000 in 2019, only half have at least 1 BHS. Figure 3 shows the provinces and HUC/ ICCs with BHS equal or exceeding the number of barangays (above green line). BHS to barangay ratio is not related to poverty incidence.

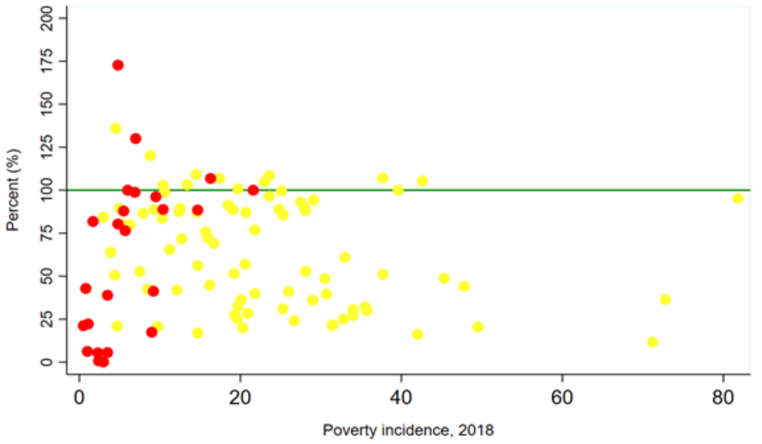

Figure 3. Barangay with BHS and poverty incidence, 2019

Note: y axis: The red and yellow dots are HUCs and provinces, respectively.

Figure 4 shows the distribution of provinces and HUC/ ICCs with access to Rural Health Units/ Health Centers (RHU/HC) within 30 minutes. On average, only half of the population has access to such facilities within this travel time. BARMM, Bicol, and MIMAROPA are the three regions with the highest share of the population without access to RHU/ HCs within 30-minutes travel time.

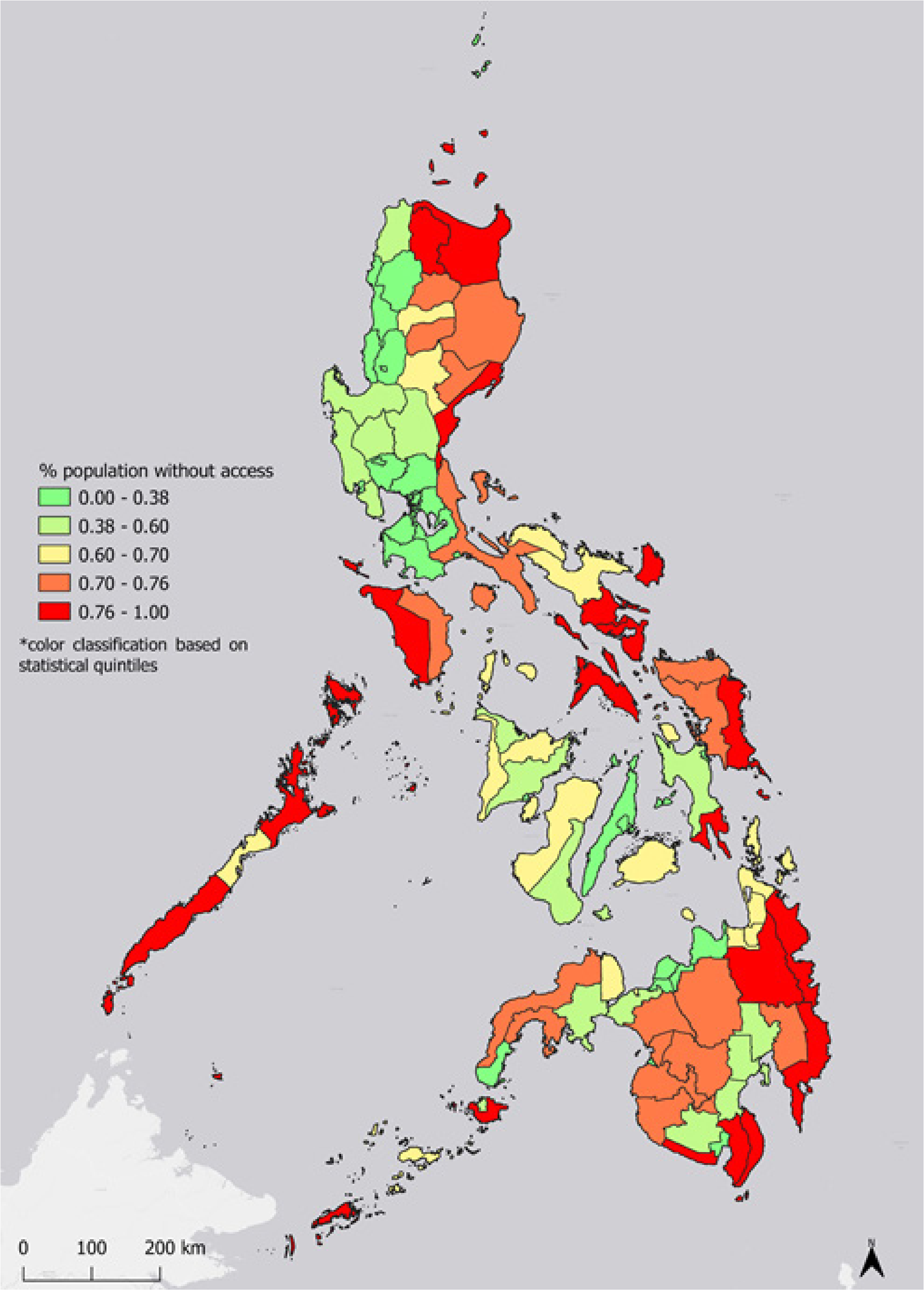

Figure 4. Distribution of populations without access to a Rural Health Unit/ Health Centerwithin 30 minutes

Hospitals are scarce. In 2018, there were 1,200 licensed hospitals in the country. While the number of hospital beds has increased over the years, it has not kept pace with the fast-growing population. The current bed to population ratio (1.2 bed per 1000 population) is comparable to those of the poorest countries in the world (World Bank, 2020) (see Figure 5).

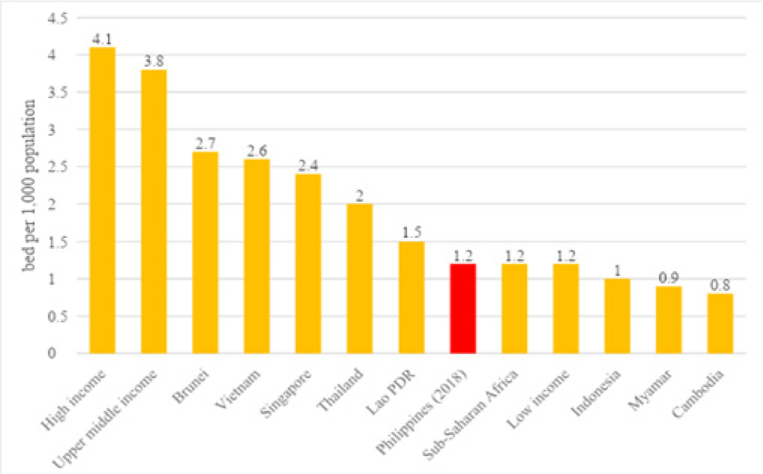

Figure 5. Bed to population ratio in ASEAN and selected geographical locations, latest available year
Source: Raw data from World Health Organization

The number of private beds has increased over the years, from 4,000 in 1990 to 6,000 in 2018 (red bar), but the bed to population ratio has declined because of the slow growth of public beds and the rapid population growth (see Figure 6).

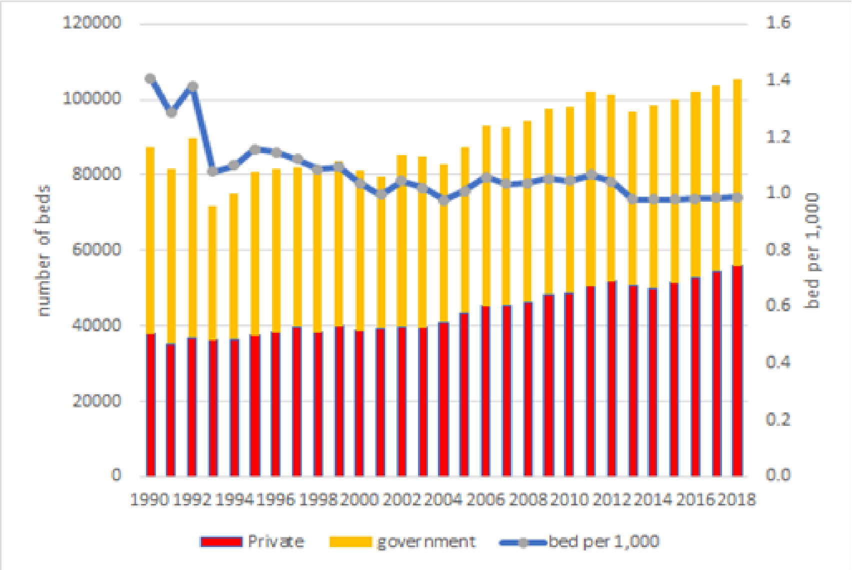

Figure 6. Number of hospital beds, Philippines, 1990-2018
Source: Raw data from Philippine Statistical Yearbook

Different levels of hospital care are limited in some provinces and regions. All provinces and HUCs/ ICCs should have at least Level 1 and 2 hospitals. However, of the 114 provinces and HUC/ ICCs, 6 (5%) and 33 (29%) lack Level 1 and 2 hospital beds, respectively.

In terms of density, only 36 have Level 1 bed to population ratio above the national average (0.36 Level 1 bed per 1,000 population) and 46 have Level 2 bed to population ratio above the national average (0.33 Level 2 bed per 1,000 population) (see Figures 7 and 8).

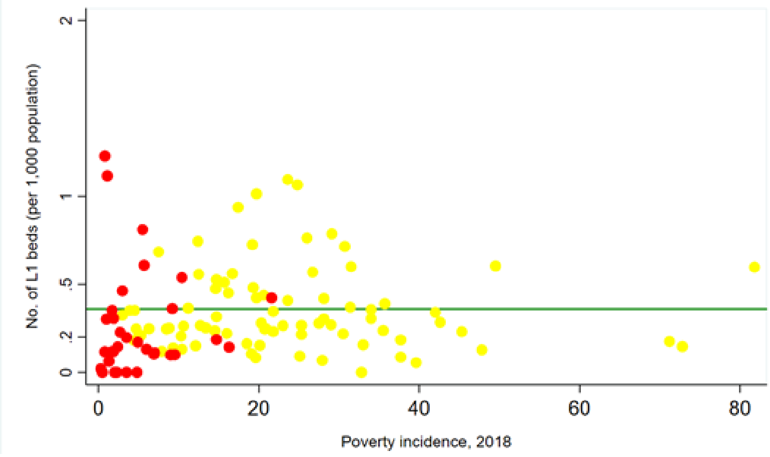

Figure 7. Level 1 hospital beds and poverty incidence, 2018

Source: Raw data from Department of Health and Philippine Statistics Authority. Note: y axis: logged. The red and yellow dots are HUCs/ICCs and provinces, respectively. Green line indicates the average.

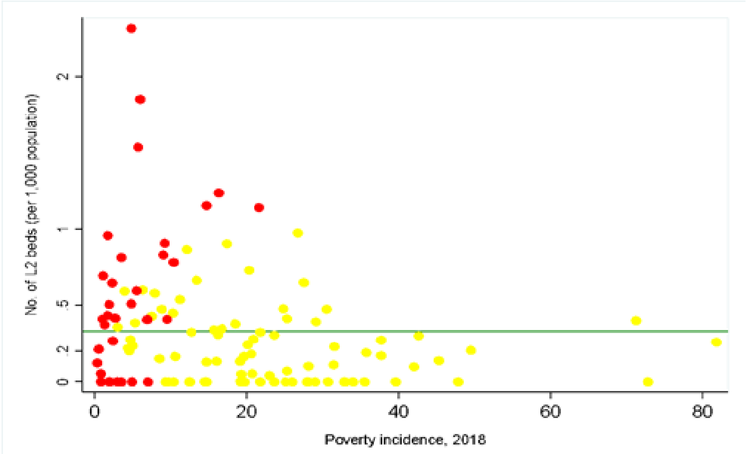

Figure 8. Level 2 hospital beds and poverty incidence, 2018

Source: Raw data from Department of Health and Philippine Statistical Authority; Note: y axis: logged. The red and yellow dots are HUCs/ICCs and provinces, respectively. Green line indicates the average.

Using region as the catchment area for Level 3 hospitals, BARMM, CARAGA, and MIMAROPA, the regions with the highest poverty incidence in the country, do not have any Level 3 hospital (see Figure 9).

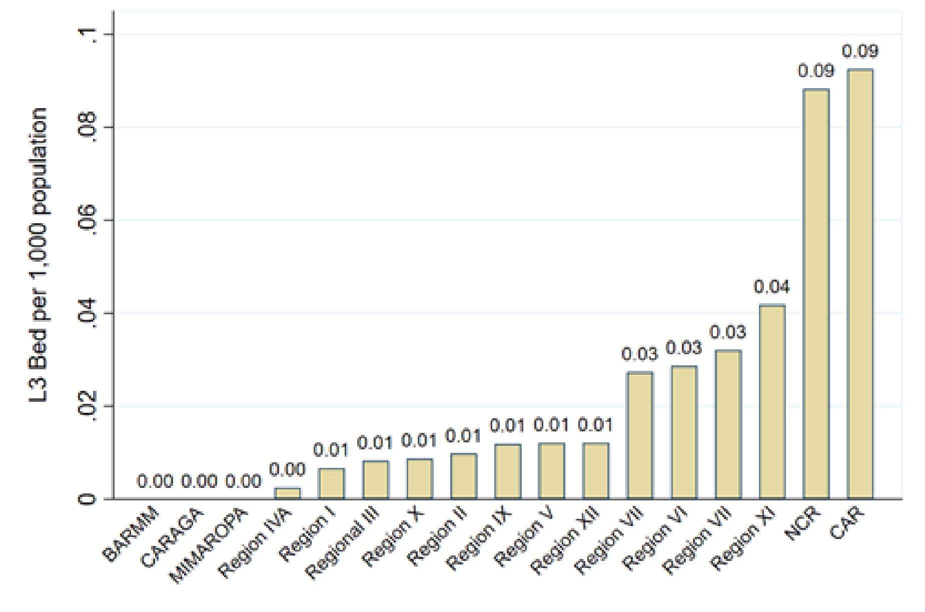

Figure 9. Distribution of Level 3 hospital beds by region, 2018

Source: Raw data from Department of Health

Medical technologies are critical in the prevention, diagnosis, and treatment of patients, but with oversupply, they may contribute to higher health spending and health system waste. The government should ensure enough supply of these technologies to meet the health needs without causing system inefficiencies.

The distribution of medical technology also varies across regions in the country - mostly concentrated in richer areas.

- The country has 1,112 X-rays or 1 X-ray for every 10,000 population.
- The country has 456 and 109 CT scan and MRI equipment, respectively. The total density of MRI is less than one per million people, which is significantly low compared to regional peers (See Figures 10 and 11).
- Majority of X-ray, CT scan, and MRI machines are concentrated in relatively affluent and highly populated regions such as NCR and Region IV-A. BARMM and MIMAROPA have no recorded MRI. About 80% of CT scans and 90% of MRIs in the country are privately-owned (See Figure 12, 13, and 14).

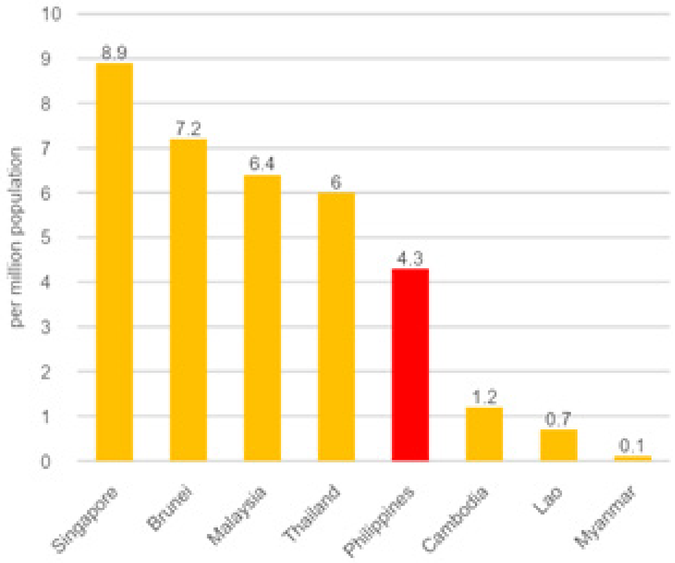

Figure 10. Number of CT scan machines per million population, by ASEAN countries, 2019

Source: Raw data from Department of Health, World Health Organization

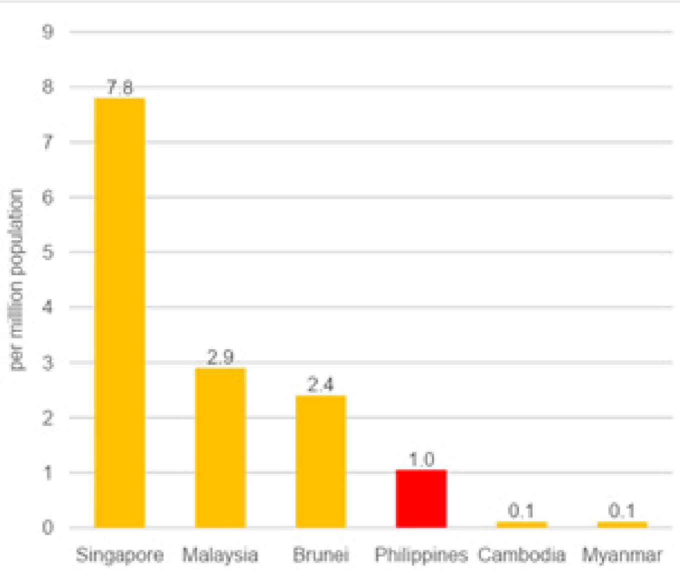

Figure 11. Number MRI machines per million population, by ASEAN countries, 2019

Source: Raw data from Department of Health, World Health Organization

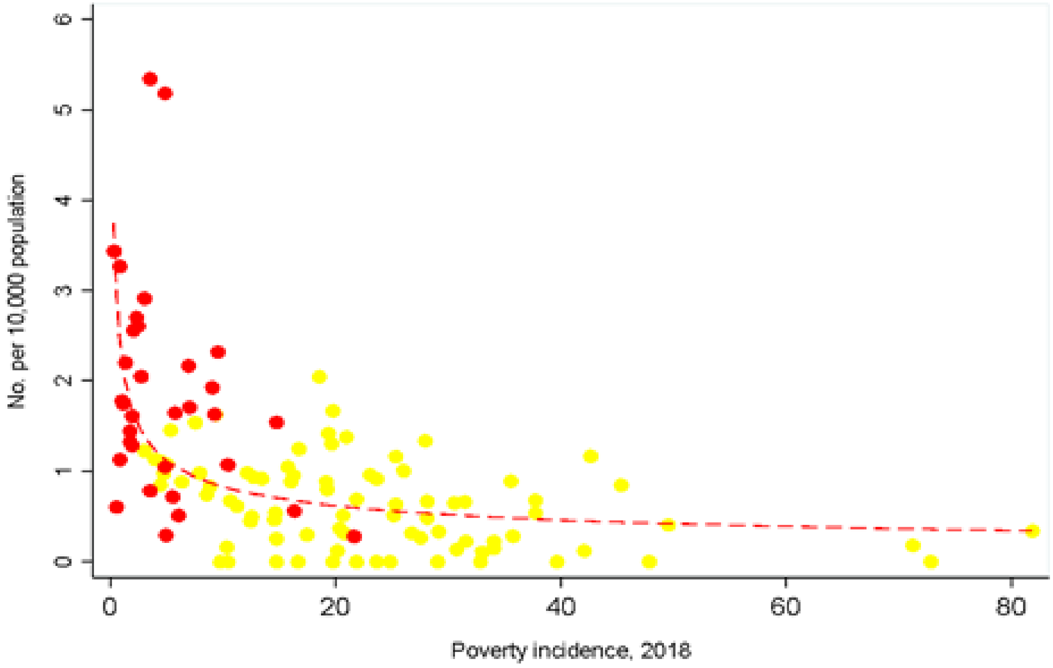

Figure 12. Number of X-ray machines, 2019

Note: y axis: logged. The red and yellow dots are HUCs and provinces, respectively

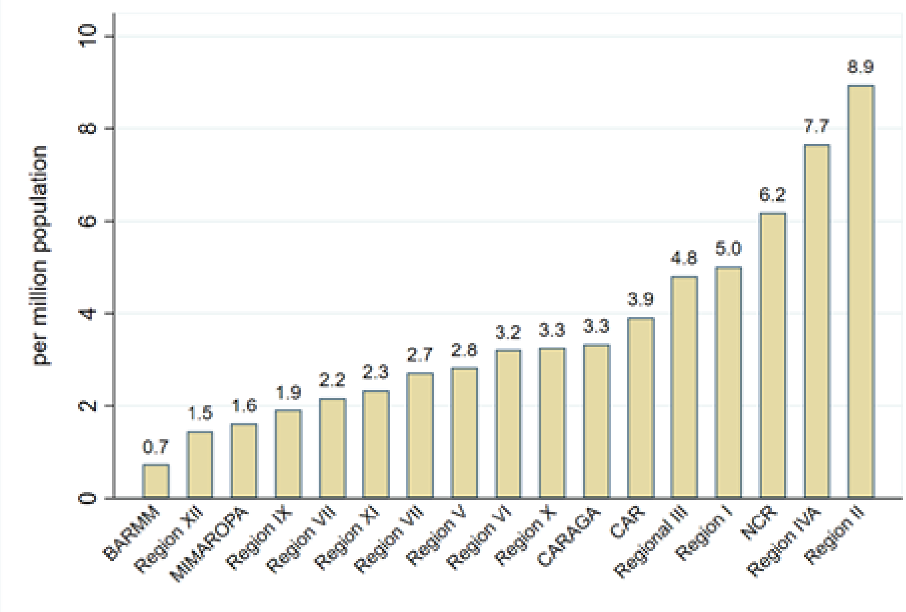

Figure 13. Number of CT scan machines per million population, by region, 2019

Source: Raw data from the Department of Health

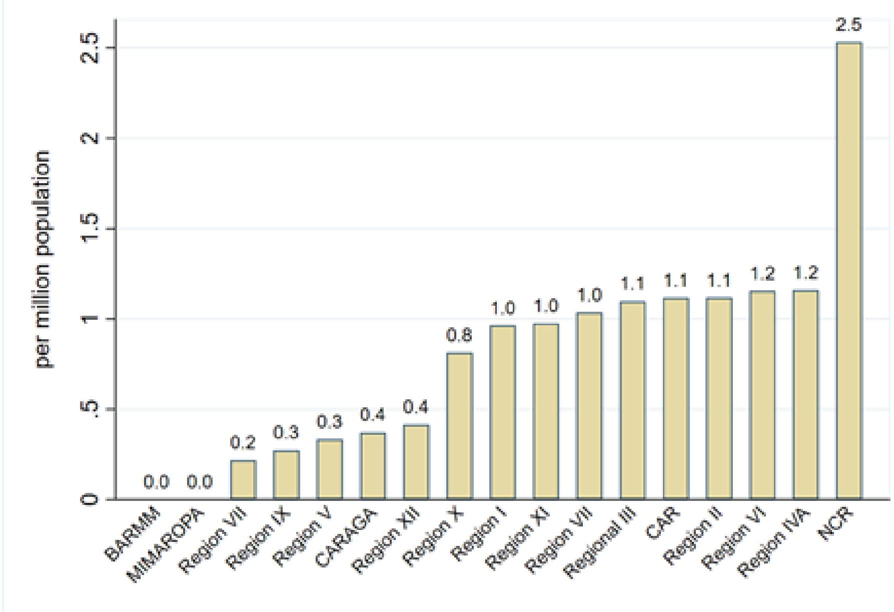

Figure 14. Number of MRI machines per million population, by region, 2019

Source: Raw data from the Department of Health

B. Health financing

In 2018, the Philippines spent PhP766 billion on health. Half (54%) were from household out-of-pocket expenses. Government expenditures (national government, local governments, and PhilHealth), only accounted for about 36% of total health expenditures (Philippine Statistics Authority, 2019); the rest

were from private health insurance and corporations (10%).

Government spending is critical to achieve UHC. In countries that have successfully implemented UHC, government spending accounts for 80% to 90% of health expenditures.

- The Philippines spends around USD50 per person for health compared to around USD 190 per person in Thailand and Malaysia, both upper middle-income countries (Figure 15).

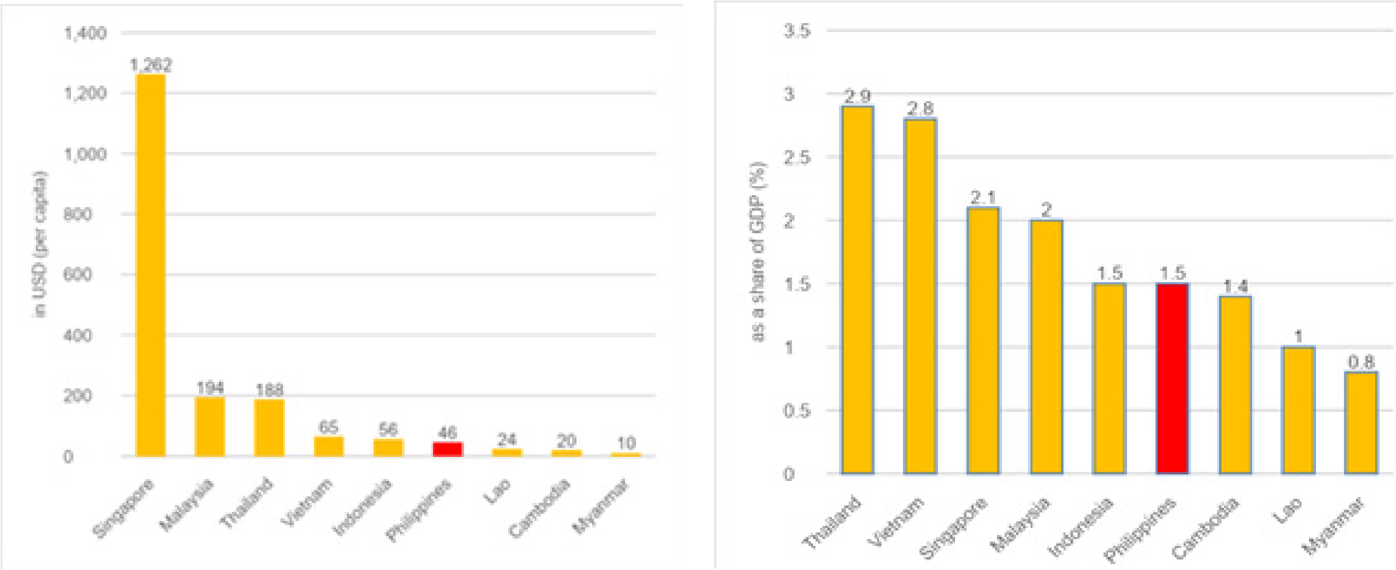

<u>Figure 15. Government spending on health in comparison to other ASEAN countries, 2018</u>

Source: Raw data from World Bank's World Development Indicators. Note: government spending includes domestic, social insurance and on-budget external sources.

- Of the PhP906 billion spent on health, PhP113 billion is accounted for capital formation, and of the current health expenditures (health expenditure minus capital formation), 30% were accounted for spending in pharmacy, 19% for general public hospital, 16% for private hospital, and only 4% for primary care facility.

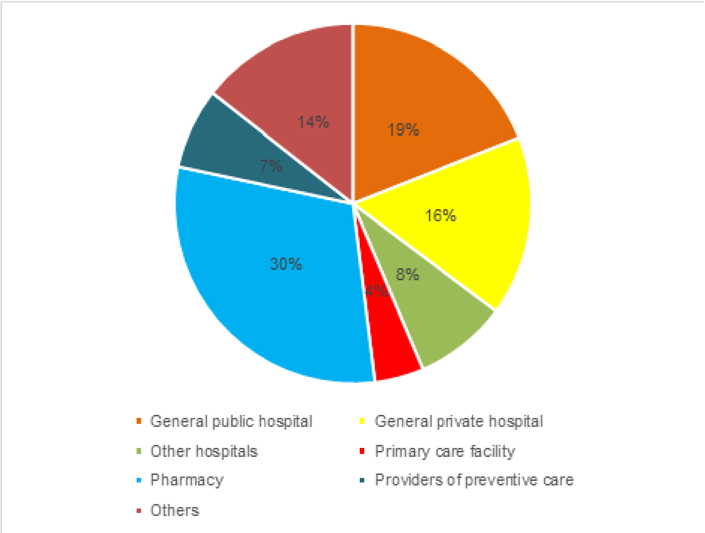

<u>Figure 16. Health spending by health provider, 2019</u>

Source: Raw data from Philippine Statistics Authority

- About 42% of the country's healthcare spending went to hospital care. Primary care only accounted for a small share (8%). The Philippines spends USD 12 per person on primary care, relatively small compared to other ASEAN countries (see Figure 17).

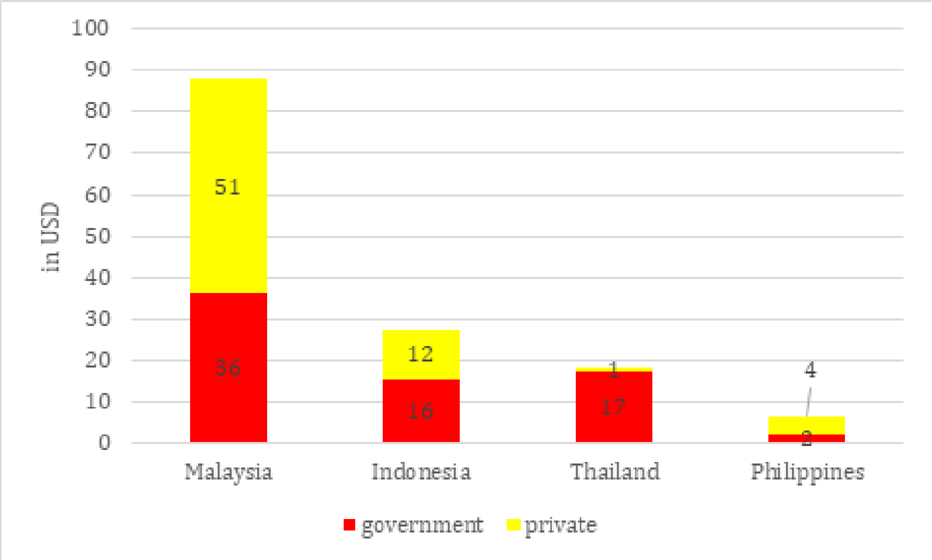

Figure 17. Estimated spending on primary care, 2018 or 2019

Source: Raw data from Philippine Statistics Authority, Ministry of Health (Thailand), Ministry of Health (Indonesia), and Ministry of Health (Malaysia).

- Of the PhP906 billion spent on health, Php113 billion was accounted for capital formation. Medical equipment and infrastructure account for about 49% (PhP55 billion) and 41% (PhP46 billion) of the total capital formation. The rest were accounted for by ICT equipment and transportation (see Figure 18).

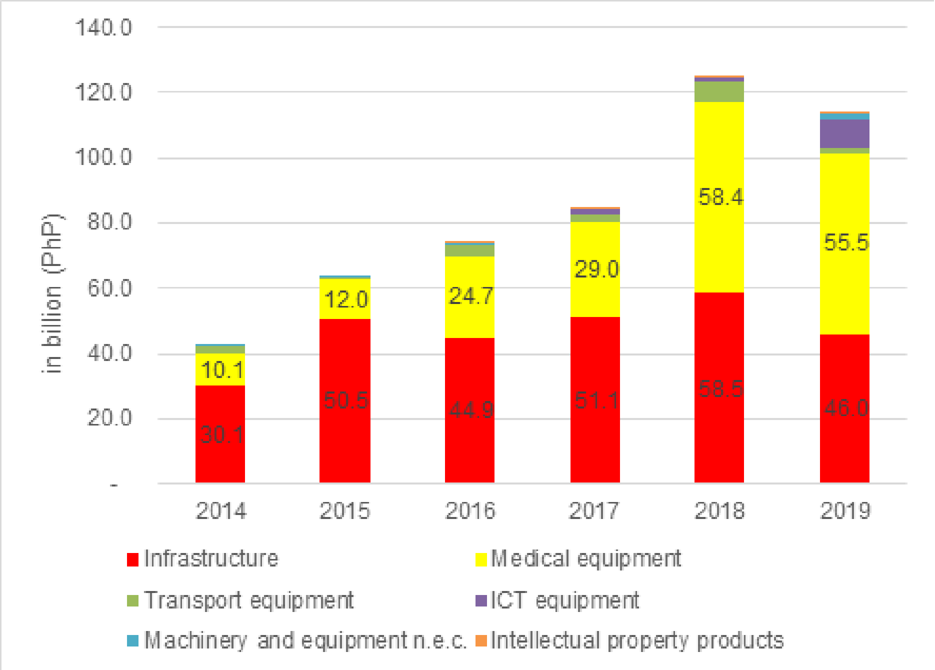

Figure 18. Spending on capital formations on health, 2016-2018

Source: Raw data from Philippine Statistics Authority

Despite decentralized service delivery and financing, the national government remains the main source of government spending. In 2018, the national government, PhilHealth, and the local government accounted for 43%, 20%, and 37% respectively.

- On average, LGUs spend PhP390 per person on health.
- Figure 19 shows the disparity in public spending per person on health by province and HUC. Rich localities spend about PhP5,500 on health; some provinces spent less than PhP20.

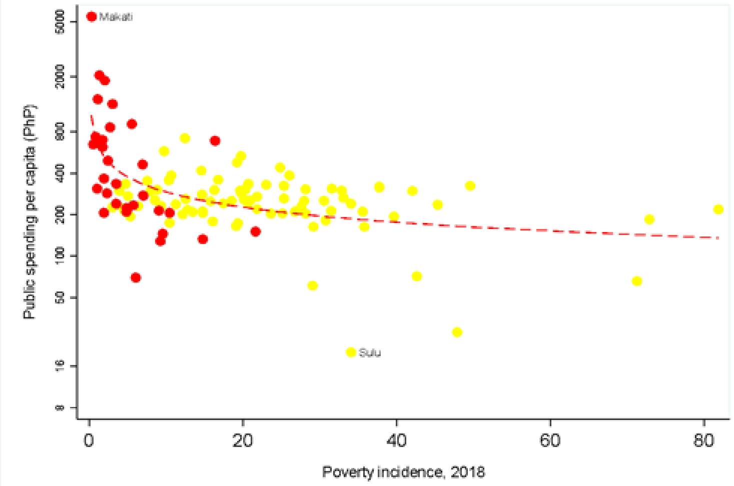

Figure 19. Public spending for health per capita, 2018

Source: Raw data from Department of Finance- Bureau of Local Government and Finance, 2018

National government spending on health increased in recent years. In 2018, the earmarked sin taxes from alcohol and tobacco were allocated to the Health Facilities Enhancement Program (HFEP), PhilHealth premium subsidies, and other DOH programs. The incremental revenue allocated for health based on Sin Tax collections in 2018 is PhP71.2 billion (43%) of the 166.7 billion total budget of DOH and PhilHealth.

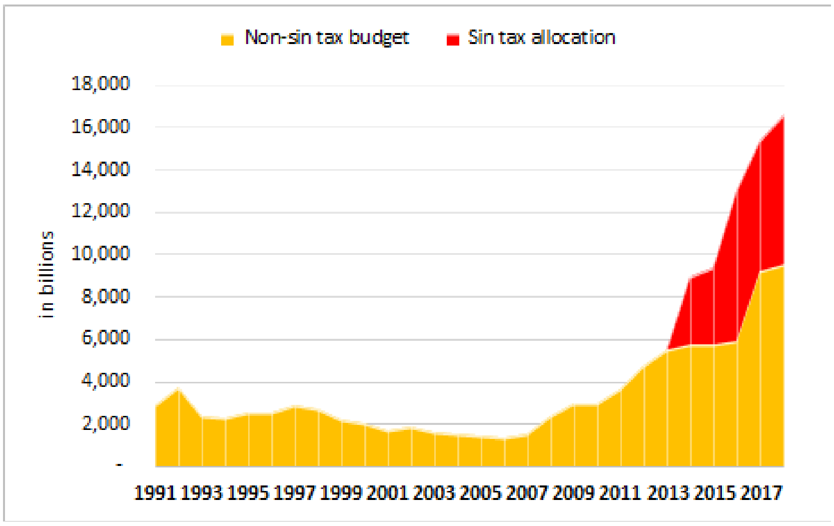

Figure 20. DOH budget (general appropriations) and sin tax allocation to health, 1990-2018 (in billions 2018 prices)

Source: Department of Health; Note: The Sin Tax Law was in effect in 2013

The HFEP, a priority DOH program, attempted to address perennial shortages in health facilities by augmenting capital investments in national and LGU health facilities.

- From 2008 to 2018, the number of projects increased significantly, but dropped sharply in 2019. Figure 21 shows the number of HFEP projects since the start of the program.
- In 2018, about 13% of the DOH budget was allocated to HFEP. HFEP obligation increased from PhP0.2 billion in 2008 to PhP22 billion in 2018.
- In 2018, while BHS and RHU accounted for the majority of projects, it only accounted for 20% of total PhP22 billion HFEP obligations.
- Majority of HFEP projects were for the construction or upgrading of BHS and RHUs.
- There were inefficiencies in the program particularly in 2018 and 2019, as evidenced by the low absorptive capacity (see Figure 22).

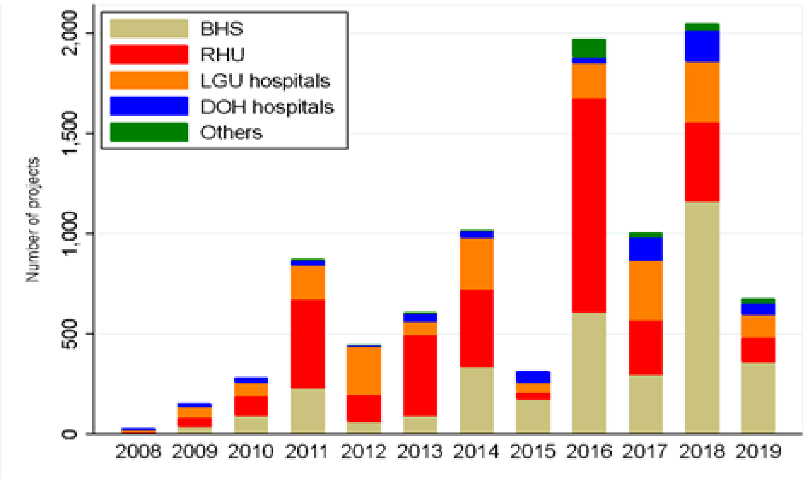

Figure 21. Number of HFEP projects, 2008-2019

Source: Raw data from Department of Health

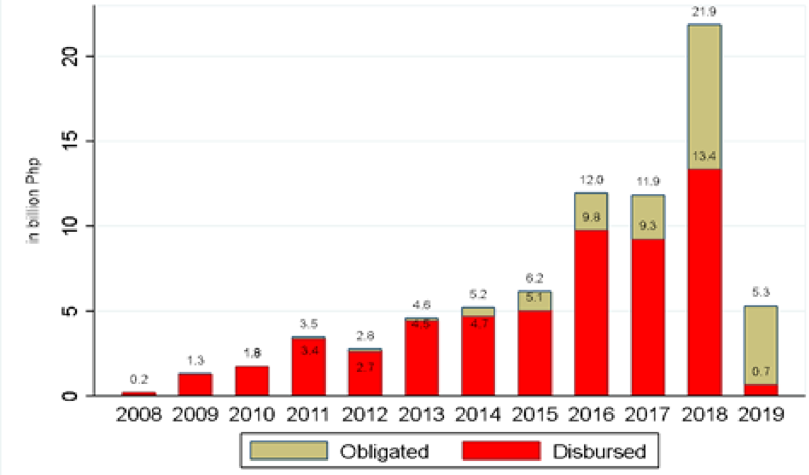

Figure 22. Absorptive capacity of HFEP, 2008-2019

Source: Raw data from Department of Health

- From an equity perspective, resources should be allocated in areas with the greatest need and

least capacity. However, HFEP expenditures were more likely to be allocated in relatively richer areas. Municipalities with higher poverty have lower HFEP expenditure. The top 10% richest municipalities received a median of Php 17 million of HFEP grants compared to Php 12 million for the 10% poorest municipalities (Figure 23).

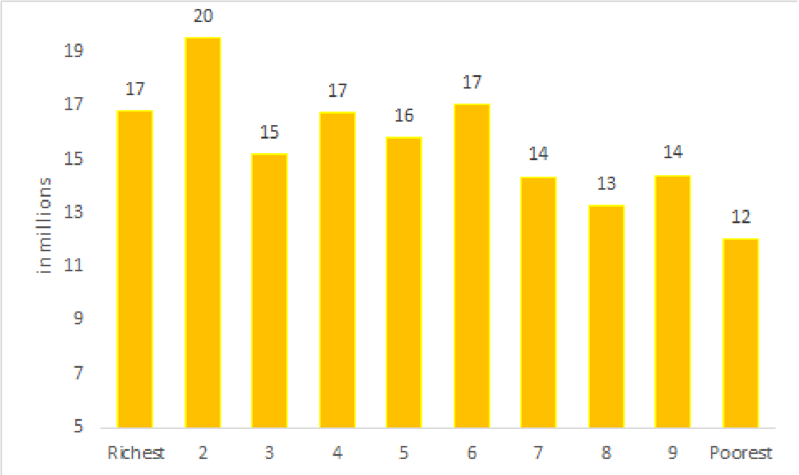

Figure 23. Median HFEP total disbursement by poverty incidence, 2008-2019

Note: The figure above only includes HFEP for LGU-owned facilities. HFEP for CHD/ DOH-retained hospitals not included; poverty incidence of municipalities and cities were grouped into deciles.

C. Human Resources

The ability of the country to achieve its health system goals depends largely on the availability of healthcare workers and the quality of their services. While the number of physicians and nurses has increased in recent years, the disparity across provinces and cities remains remarkably striking. Figure 24 shows that most physicians are concentrated in HUCs/ ICCs and relatively rich provinces.

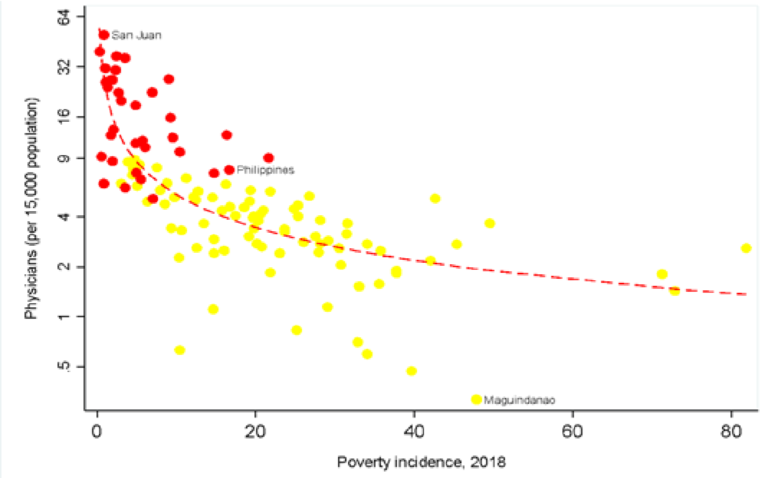

Figure 24. Availability of physician and poverty incidence, 2018

Source: Raw data from Philippine Census, Philippine Statistics Authority

In RHUs, scarcity of human resources remains a major challenge. Only 90% of RHU/CHUs in the country have at least one medical doctor; a substantial portion of RHU or CHU do not have nurse or midwife. In BHS, about 80% have at least midwife and 90% have BHW. Less than 10% have MDs and nurses. The level of scarcity of health workers in government primary care facilities varies across provinces

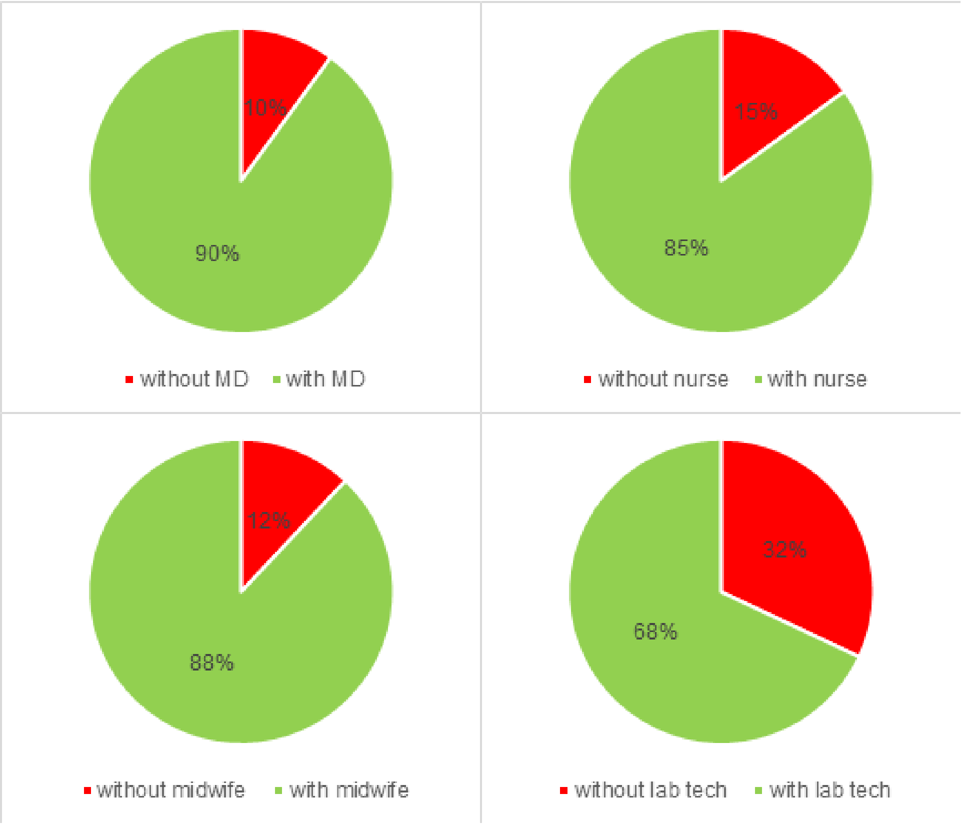

Figure 25. Availability of health workers in Rural Health Units, 2019

Health human resources are not limited to physicians, nurses, and other allied health professionals. It also includes non-medical professionals supporting the complex operations of health facilities and public health interventions, such as managers, finance officers, supply chain experts, and IT managers and technicians. Analysis of the geographical distributions of the backend workforce of IT managers and supply chain managers mirrors the inequitable distribution of frontline health human resources.

D. Health information Systems and Communication technology

The Philippines is not starting from scratch in implementing eHealth solutions such as electronic medical records (EMRs), telemedicine, and telehealth. Primary care facilities and hospitals have begun using EMRs to improve efficiency and decision-making. PhilHealth now requires health facilities to submit electronic insurance claims.

However, the adoption of eHealth solutions remains limited and it varies between public and private healthcare providers. Only one-third of rural health units are using electronic medical records (EMRs). The majority of RHUs remain non-compliant with EMR requirements. Relative to public providers, private hospitals have significant investments in digital tools with gradual integration into their workflow.

Telemedicine and telehealth projects initiated by DOH, academic institutions, donors or other private enterprises do exist. One example is the use of tele-radiology, a remote radiologist interprets radiologic results while on the other location. Although utilization of the technology is growing, it remains underutilized.

While there have been several successes in the implementation of EMRs and telemedicine, the results have been mixed. It is also challenging to leverage the success of these initiatives across sectoral and geographic boundaries because of varying degrees of investments and approaches of the national and local government units (see Box 1).

Box 1. The growth of eHealth at the time of COVID-19 pandemic

In 2020, the COVID-19 pandemic stretched the human, financial and technical resources of both the Department of Health and LGUs. While the devastation caused by the COVID-19 is immeasurable, it also showed remarkable gains on the use of digital health solutions to address the burden of COVID-19 while also working to keep the health system from breaking down. Several health ICT interventions were swiftly deployed at national and local level although the progress of its roll-out has been modestly adopted before the COVID-19

Among these are the use of big data analytics and business intelligence, telehealth/telemedicine, mHealth, and open data. Before the COVID-19 pandemic, telehealth and telemedicine services were used sparingly with low buy-ins from doctors at both private and public health facilities. These are due to the absence of standards on patient billing and reimbursement, lack of patient confidence in the online consultation, and some medico-legal issues. With the adoption of physical distancing measures to avoid further outbreaks, there was a sudden rise of physicians and hospitals that shifted to telehealth and telemedicine consultation.

Example of Teleconsulting Services during the Expanded Community Quarantine in the Philippines.

The DOH also boosted its health promotion campaigns through virtual channels and social media. Several health promotions materials and infographics were released daily to inform the public on COVID-19, virtual pressers were also used in place of a traditional press conference for daily updates about the status of COVID-19.

Although we saw the pivotal role of eHealth solutions during the COVID-19 pandemic, it also exposed some of the underdeveloped eHeath components such as the lack of interoperability of EMR and several health information systems (ability of health information systems to work together with other health information systems). Additionally, the protracted transition to automation on both the public and private health facilities resulted in taxing encoding processes to the health care workers and analysts. For example, hospitals and referral laboratories still do paper-based or manual encoding of patients' medical and health information and demographic data. Patients and health care providers are also overwhelmed by the massive number of hospital and PhilHealth forms that require them to put in redundant data and information. Data validation among COVID-19 cases and suspected contacts have also been a cumbersome task to the DOH and LGU staff. Because they must do it manually, they need more workforce to carry out the roles, and it may also be prone to encoding errors. Another issue is that HIS from various levels of bureaucracy occurred in silos without proper governance structures. Thus, the DOH was pushed to create a new ad-hoc system for supplies and logistics tracking of COVID supplies and commodities on health facilities.

E. Governance

In a decentralized health system, provinces and municipalities are expected to delivery health services. The national government sets the national policies and standards; provides technical and financial assistance to local governments; and operates 80 national hospitals, most of which are specialty and end referral hospitals.

Under the national government are provinces. In a decentralized system, the national government does not have power over provinces' health service delivery function. Provinces own and operate provincial and district hospitals; provide technical assistance to municipalities and cities; monitor the performance of municipalities and cities.

Provinces do not have power over municipalities and cities' health service delivery function. Municipalities and cities deliver primary healthcare services in rural health units (RHUs) or city health centers (HC). Relatively rich urbanized cities and municipalities own and operate hospitals. Under municipalities/cities are villages or barangays. They deliver basic healthcare services mostly health promotion and primary prevention through barangay health stations (BHSs). They function as extensions of RHUs.

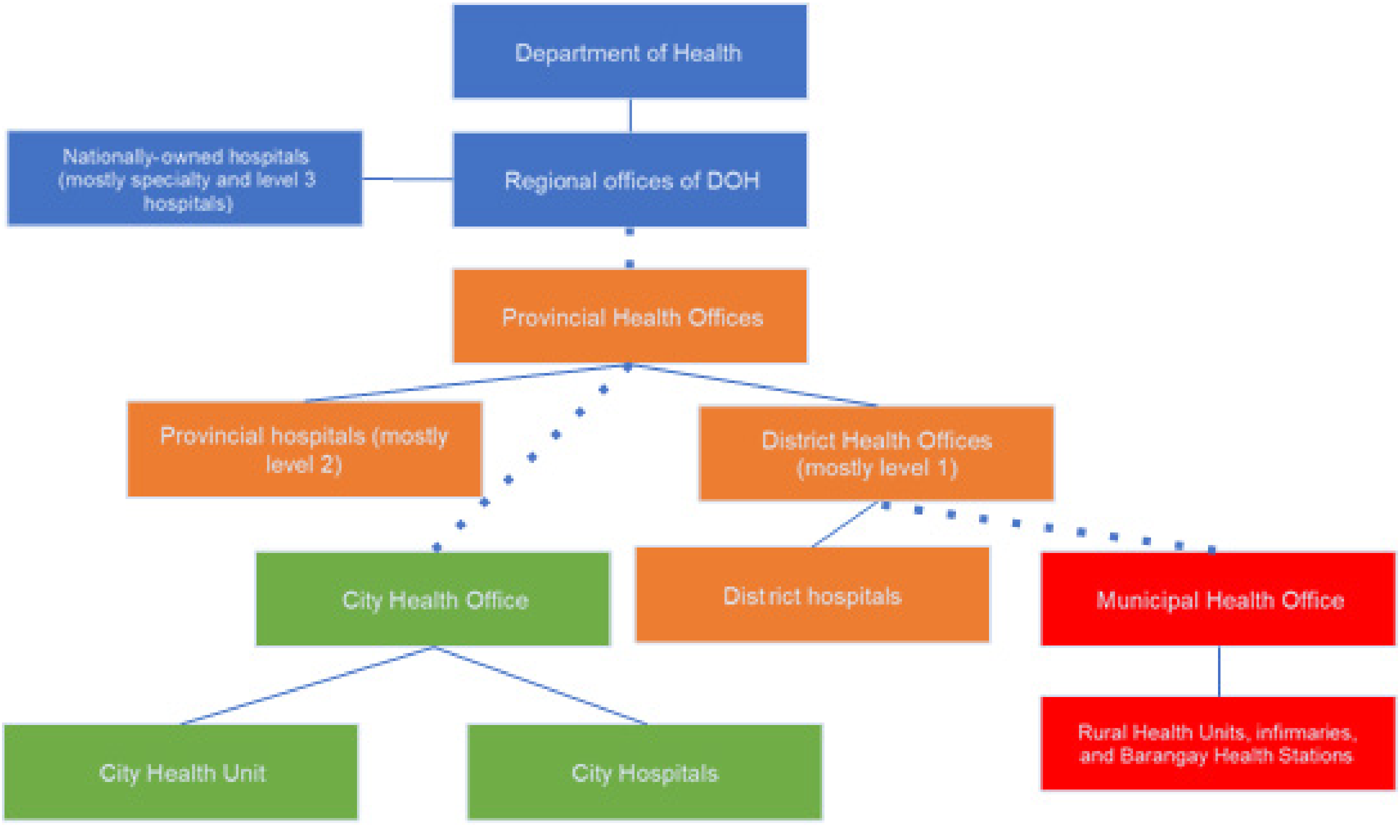

Figure 26. Governance structure of PHL healthcare system

A large private sector is working in parallel with the public health system. They provide a wide range of healthcare services similar to the public system. Private health facilities provide healthcare independently catering to the richer segment of the population. They are not formally integrated to the public system in providing comprehensive and coordinated healthcare services. Informal referral systems between private and public health facilities seem to be a common practice.

The functions of national and local governments appear to have a clear delineation and accountability. In practice, however, this is not the case. While primarily on policy development and stewardship, service delivery and financing remain as important functions of DOH. The national government/ DOH delivers and finances health services to LGUs through subsidies on capital outlay, drugs and vaccine, equipment, and human resources.

These subsidies of the national government are mostly stop gap measures, and need further assessment whether they complement local government resources, reduce inequities or improve the performance of local governments. Currently, capital outlay subsidies are based on requests from local governments.

The realities of a highly decentralized governance structure make the integration of care challenging. Municipalities and cities own and operate primary care facilities and provinces own and operate district and provincial hospitals. The different levels of care are under the auspices of different political jurisdictions and leadership makes integration of healthcare services and referral system politically challenging to implement.

F. Macro-trends that will Influence demand for healthcare

Socio-demographic, economic, technological, and environmental changes could affect the country's need for health care in the long-term.

i. Demographic and Epidemiologic Changes

The Philippine population was 105 million in 2015. This is expected to grow to 128 million by 2040, with an annual growth of 1.6%. The Philippine population is young relative to its regional peers. However, it is projected that the number of individuals 65 years and older will increase as the life expectancy improves, and the share of under-five children will decrease as the total fertility rate declines.

As the median age increases, non-communicable diseases (NCDs) will also increase. NCDs are costlier and require long-term care. A robust primary care system with good continuity and quality of care will be critical in the prevention and control of NCDs. Figure 27 shows the increasing share of NCDs to the country's total disease burden.

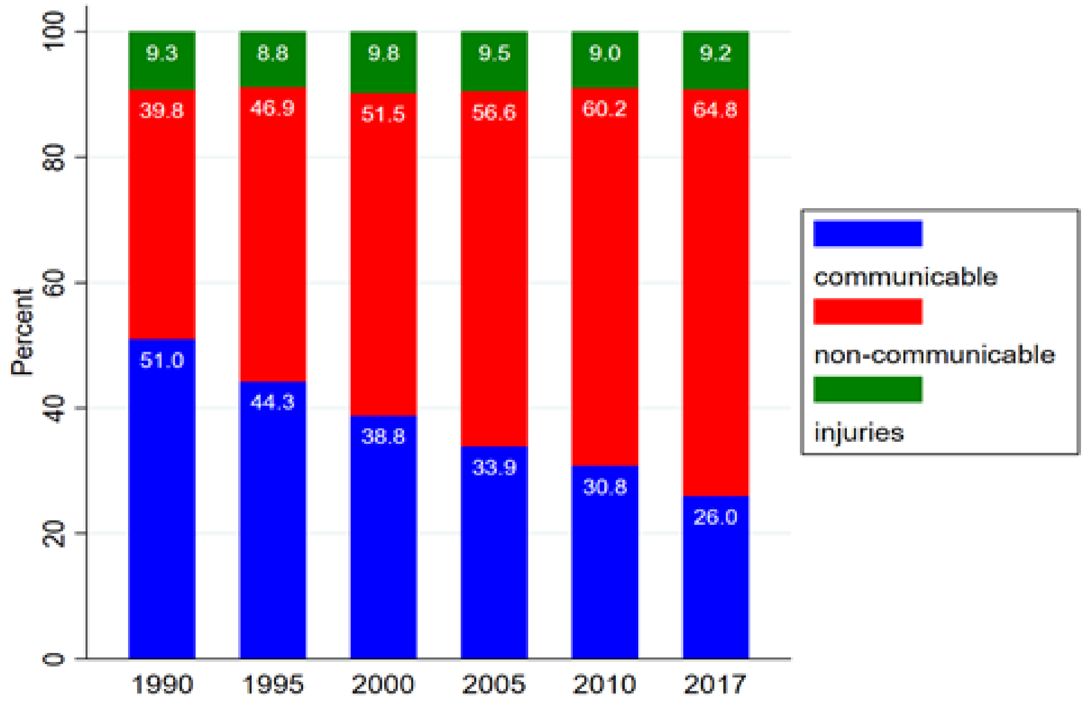

Figure 27. Burden of Disease in the Philippines, 1990-2017

Source: Raw data from Institute for Health Metrics and Evaluation

ii. Macro-economic changes

In the last decade, the Philippines experienced rapid economic growth, which resulted in vast improvement in per capita income. The conducive macroeconomic environment was expected over the medium-term, with economic growth projected at 6% every year. The country was projected to become an upper-middle-income by 2021. This rapid growth in income was expected to increase the demand for healthcare.

However, the COVID-19 pandemic has spread with unprecedented speed, infecting thousands of Filipinos. The economy was brought to a halt as the country restricted population movement to slow down the spread of the virus. As a result, the Philippines is expected to experience a historic contraction of real GDP and to bounce back by 6% in 2021 (World Bank, 2020) (see Figure 28).

Prior to the COVID-19 pandemic, the macro-fiscal position of the country was robust. Inflation rate was within the government target (2-4%), and government expenditures have generally followed increasing government revenues. Because of prudent fiscal reforms over the years, debt to GDP ratio has decreased from almost 70% in early 2000 to 40% in 2018. This indicates an opportunity for the government to increase fiscal space for health.

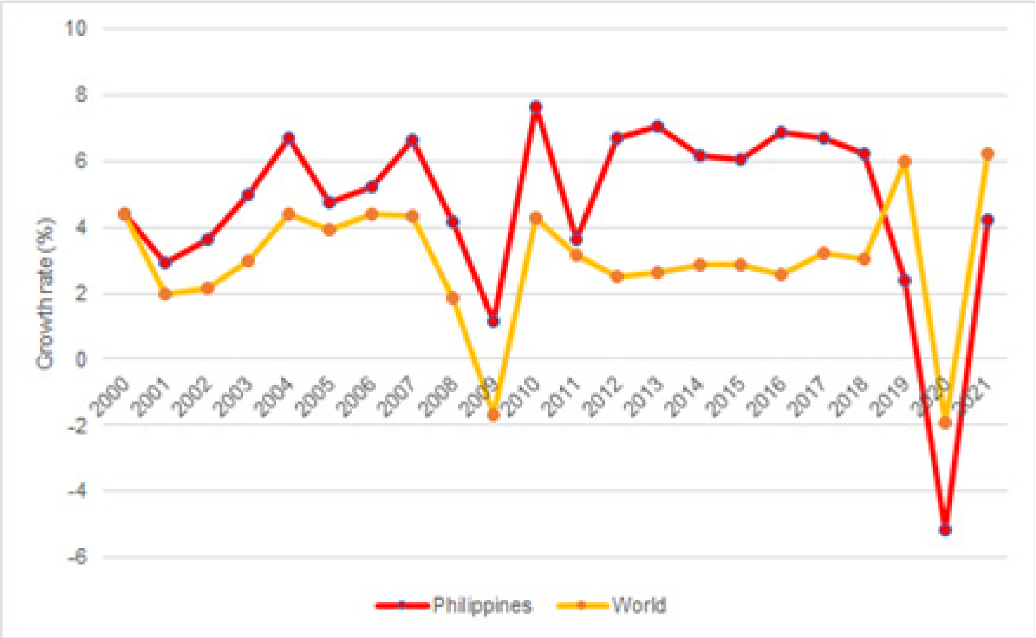

Figure 28. Real GDP growth, 2000-2021

Source: World Bank (2020)

iii. Resilience and environmental sustainability

The Philippines is one of the most disaster-prone countries in the world. Majority of the disasters are meteorological and hydrological (for example, storms and floods) and geophysical (for example, earthquake), and biological (for example, epidemic) in nature. For storms, floods, and earthquakes, the number of events and the number of affected has been increasing in the last decade (See Figure 29).

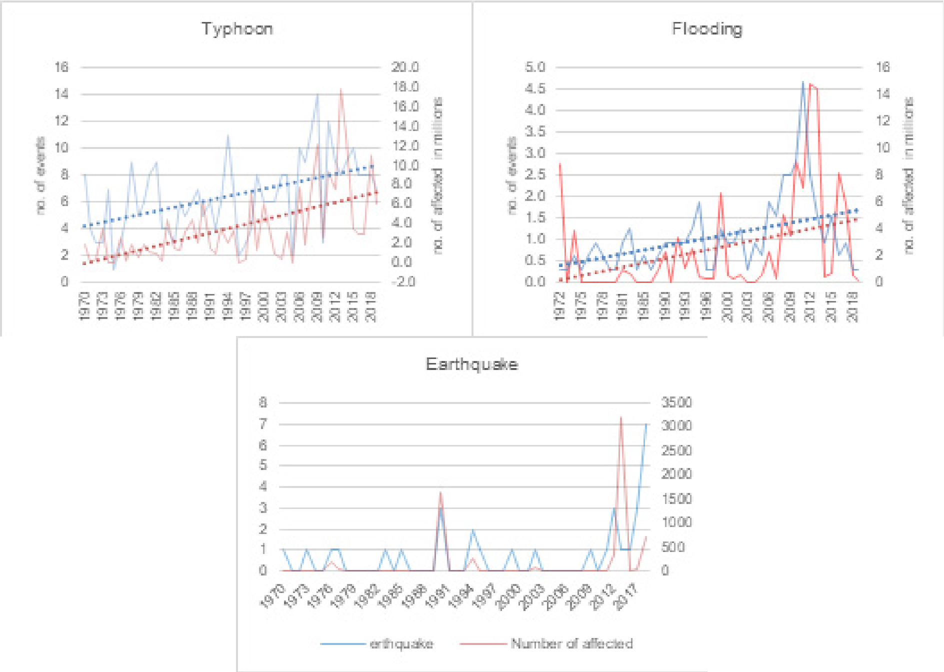

Figure 29. Typhoon and flood disasters in the Philippines

The interaction of these hazard events with social factors determines the risk. The effects of these hazards largely depend on the social vulnerability of the population (for example, poverty, gender, age – old and under-five, disability, health, and informality). The high exposure to hazards and the high level of social vulnerability make the country at risk. The Philippines is one of the most at-risk countries in the world when it comes to disasters. Figure 30 shows the country's risk index score (the higher the riskier) compared to others in ASEAN.

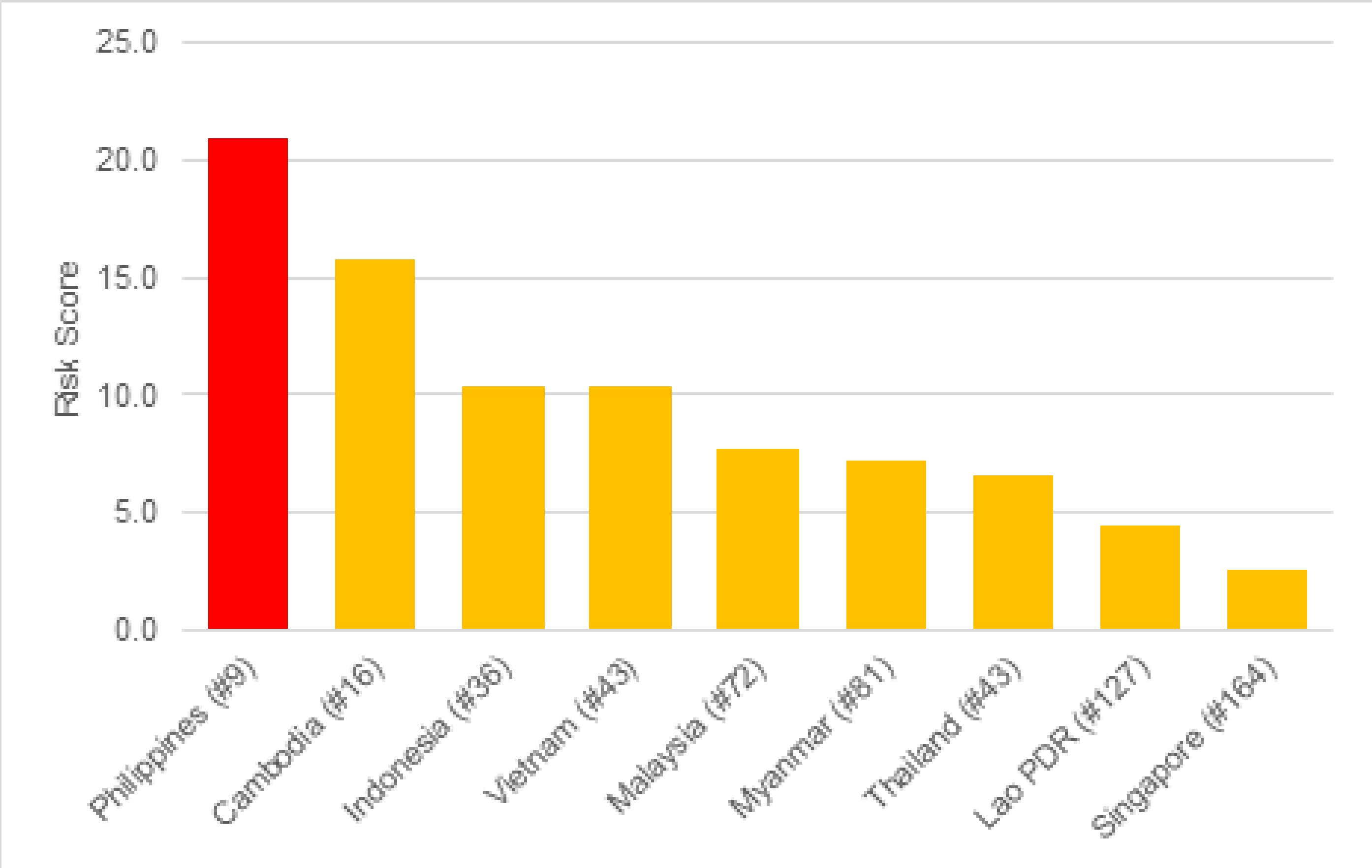

Figure 30. World Risk Index, 2020

Source: Bündnis Entwicklung Hilft and Ruhr University Bochum – Institute for International Law of Peace and Armed Conflict (IFHV). The number in the parenthesis is global ranking.

These disasters have dramatic consequences on health and the healthcare system. They do not only result in death tolls, but also lead to widespread morbidities, both infectious and chronic diseases.

Hazards affect the structural integrity of health facilities as well as the support system that these facilities and their communities depend upon. Destruction of health facilities reduces the ability to respond to the direct victims of disasters. In 2013, almost 800 health facilities were destroyed in Eastern Visayas during Typhoon Haiyan.

For health facilities to respond to the healthcare needs of the population during and after disasters, they must be resilient. In the context of COVID-19 pandemic, a resilient health system means that there are sufficient health workers and health facilities to accommodate the surge in both COVID-19

and non-COVID patients. During earthquakes or typhoons, the features of health facilities – (1) structural and non-structural components, (2) energy source, and (3) water, sanitation and chemical waste management remain functional (WHO, 2020).

Figure 31 shows the availability of power generators, electricity, and water in toilets. These are critical elements in resilient health facilities suggested by WHO.

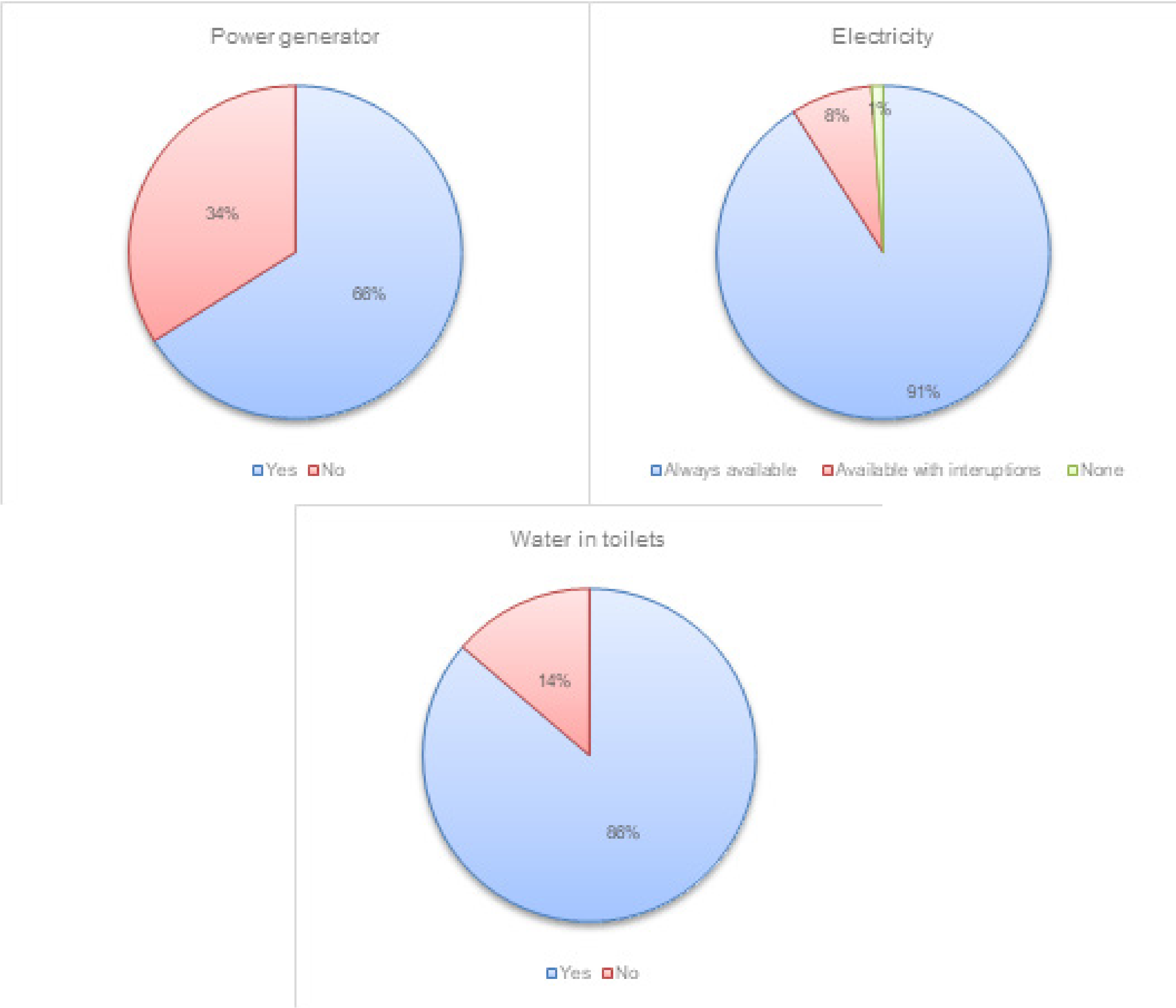

Figure 31. Availability of power generator, electricity, and sanitation facilities in RHUs

CHAPTER II

Vision: A Modern, Resilient, and Sustainable Healthcare System

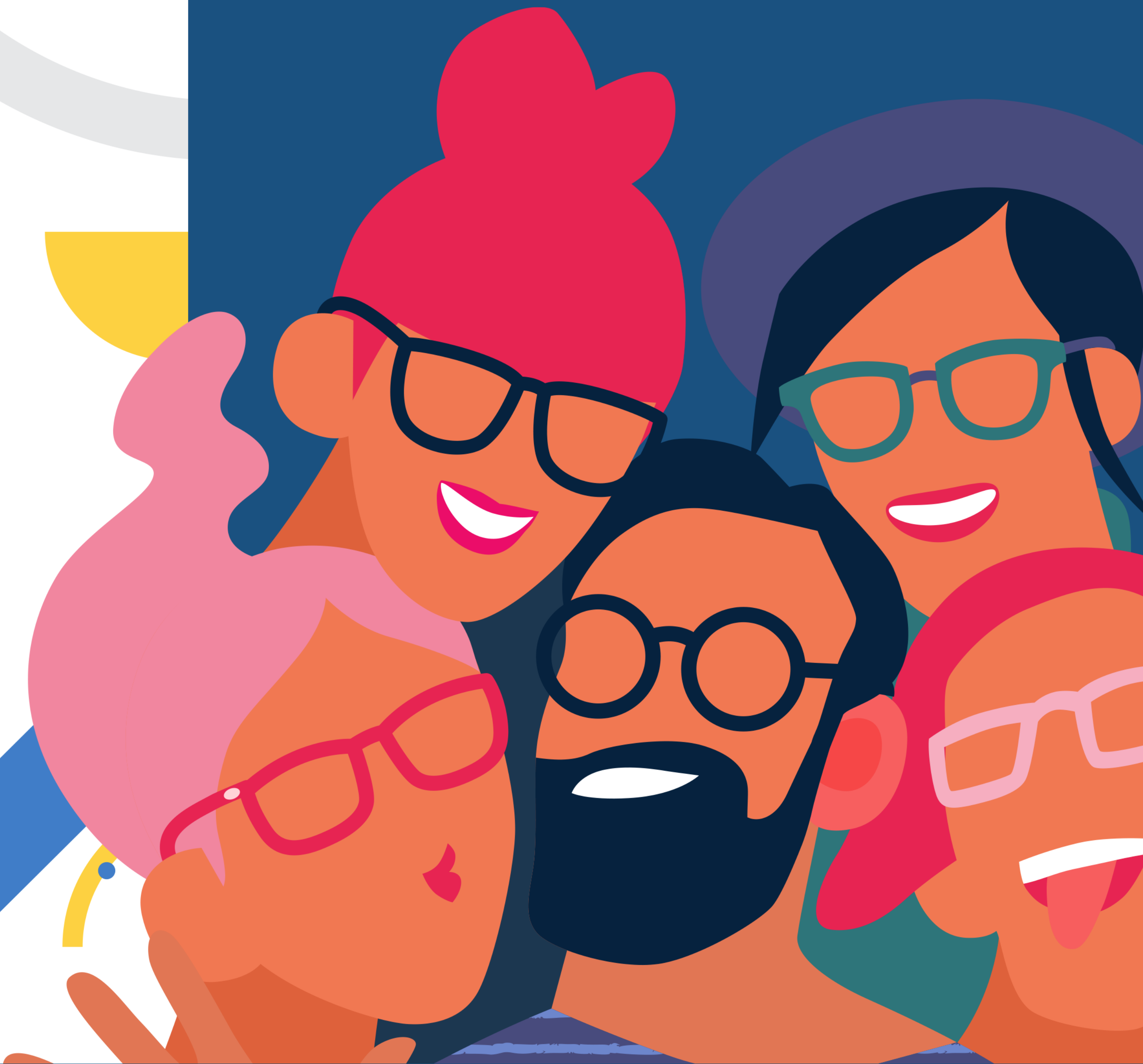

A. The Vision of Universal Health Coverage

Aligned with AmBisyon Natin 2040, the country aspires that Filipinos will be among the healthiest people in Asia. There are three strategic goals to achieve this vision:

- Improved health outcomes. Filipinos are healthy throughout their life course.
- Improved financial protection. Filipinos are not impoverished or pay an excessive share of their income for healthcare needs.
- Improved responsiveness. Filipinos experience dignity, respect, and prompt attention when they go to health facilities.

The UHC Act of 2019 provides the basis to pursue health reforms and achieve these strategic goals. One of the important provisions of the Act is transforming an equitable, primary care-oriented, and integrated healthcare system through the creation of province or city-wide health care provider networks (HCPN) where both public and private health facilities are integrated to provide coordinated and comprehensive healthcare. Integration also means coordination of non-clinical functions through, for instance, sharing of electronic medical records (EMR) across facilities in the HCPN.

What does a HPCN look like? At the minimum, HCPN consists of (see Figure 32):

- Primary care provider network (PCPN); BHS and Primary Care Facility (PCF); composed of primary care services.
- Levels 1 and 2 hospital
- Ancillary facilities such as private medical outpatient clinics, infirmaries, standalone birthing homes, standalone laboratories and dental clinics

The HPCN is linked to an apex hospital, a level 3 single-specialty or a multi-specialty general hospital, which serves as the end referral center.

The primary care facility shall serve as the first point of contact of patients, families, and communities with the healthcare system to access basic and comprehensive primary care. If a higher level of care is needed, they will be referred to hospitals (Level 1 or 2) or standalone ancillary or specialized facilities within the HCPN. Level 2 hospitals will provide intensive care services and some specialty care. Those needing complex specialty care will be referred to an affiliated Level 3 general or specialty hospital at the regional, subnational, or national levels.

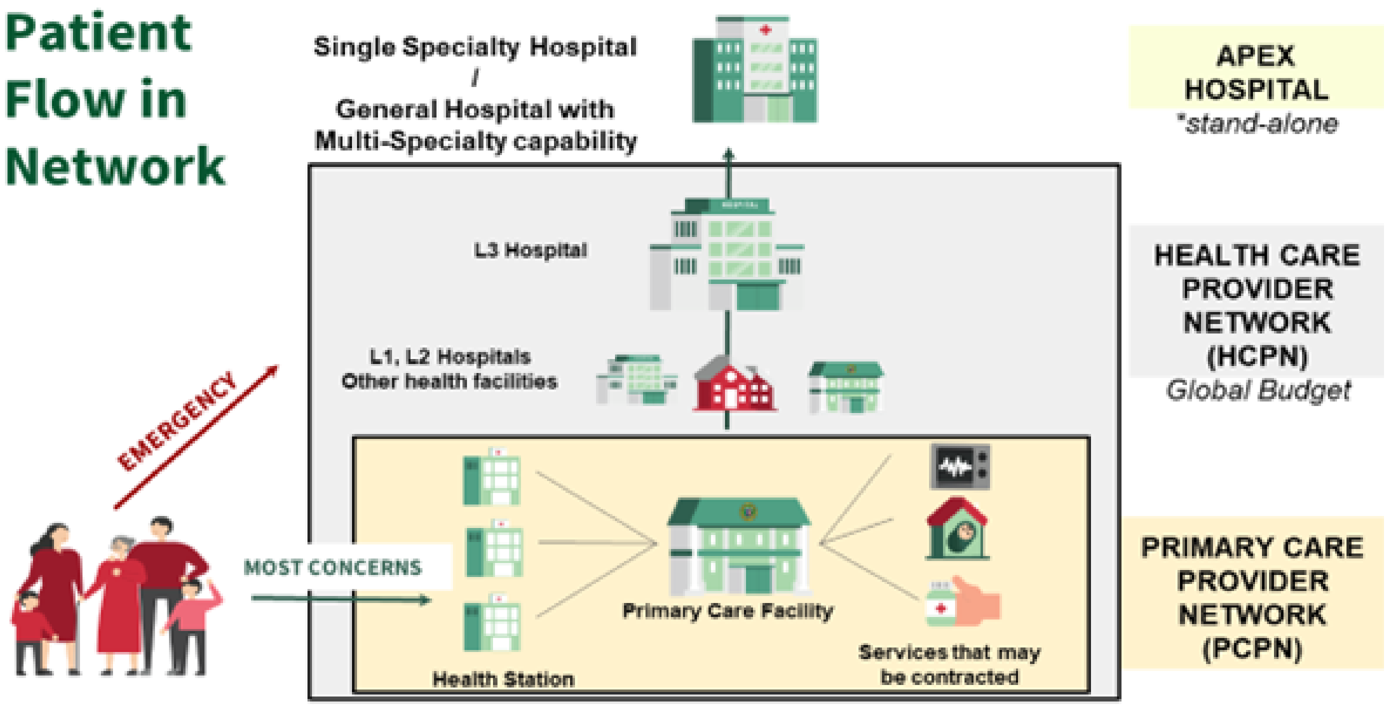

Figure 32. Patient flow under UHC

*Note: *Apex hospitals are contracted as standalone facilities by PhilHealth*

In a HPCN, it is the responsibility of the province or HUC to ensure that they have adequate health facilities to meet the needs of the population. The provincial government has the leeway in ensuring their availability, financed primarily using local government resources. This can be done through the following:

- Building and expanding publicly-owned BHS, RHUs, levels 1 and 2 hospitals, and ancillary facilities.
- Encouraging privately-owned clinics and level 1-2 hospitals to be part of the HPCN complementing the publicly-owned system.
- Encouraging privately-owned ancillary facilities (that is diagnostics, pharmacy, ambulance systems) to be part of the HPCN.
- Tapping the private sector to build and manage clinical and non-clinical functions of the HPCN.

While provinces and HUCs do not need to have referral hospitals (Level 3 and apex hospitals) within their geographical area, they need to be attached to one.

- Level 3 or apex hospitals (public or private) located within the province or HUC could be tapped as the end referral hospital. Other provincial HPCN could contract out the same facility.
- Level 3 and apex hospitals should cater multiple provincial HPCNs. The local government with the support of the national government should ensure the availability and adequacy of these health facilities.

Table 2. Roles of health facilities under UHC

	Primary care provider network within HCPN	Health facilities within HCPN	Apex Hospital (multi-specialty) outside HCPN	Apex Hospital (single specialty) outside HCPN
Role	Primary health care (individual and population-based)<br><br>Delivery of public health interventions (main)	Inpatient general care (level 1-2 hospitals), Specialized care, Transition care<br><br>Delivery of public health interventions	Level 3 hospitals, providing general to specialty care (with focus on designated specialty care)<br><br>For designated specialties:<br>• Specialty care<br>• Oversight for health workforce training & distribution<br>• Data repository for specific specialty<br>• Multi-center research	Specialty care<br><br>Oversight for HR training & distribution<br><br>Data repository for specific specialty<br><br>Multi-center research
Scope	Barangay, municipality	Province, HUC and ICC	Multiple HCPNs (Regional, Sub-national)	National
Facilities	Primary care facilities (RHU, HC, Private Medical Outpatient Clinic), Health stations, Infirmaries, Birthing homes, Dental Clinics	District & Provincial Hospitals, General hospitals, Specialized health facilities, Transition care facilities, Diagnostic facilities	Regional Hospitals, Medical Centers, Designated multi-specialty Centers	Single-specialty hospitals,

Governance and financing reforms as enacted in the UHC Act should be fully implemented for HPCN to realize:

- o *The creation of special health fund (SHF) and provider payment reforms (that is prospective global budgets)*
- o *The expansion of PhilHealth's primary care benefits*
- o *The implementation of equity framework to rationalize national government resources*

B. Resilient and sustainable health system: necessary in achieving UHC

The National Objectives for Health (NOH) 2017-2022 aims for a resilient health system, which is defined as the capacity of the system to absorb, adapt, and transform when exposed to shocks and still retain the same control on its structure and functions to help the country prepare for and respond to disaster. The inclusion of climate-resilient and environmentally sustainable approaches is essential in ensuring continuous performance of the health system during disasters.

For the health system to become climate-resilient, its building blocks (that is, service delivery, health

technology and infrastructure, health financing, health workforce, leadership and governance, and health information), which are necessary to support UHC, should be climate-resilient as well. The primary goal of UHC is to promote equitable healthcare access, it is therefore critical that even during disasters, basic healthcare services should be maintained and accessible to everyone. Figure 33 shows the resilience components under each health system block.

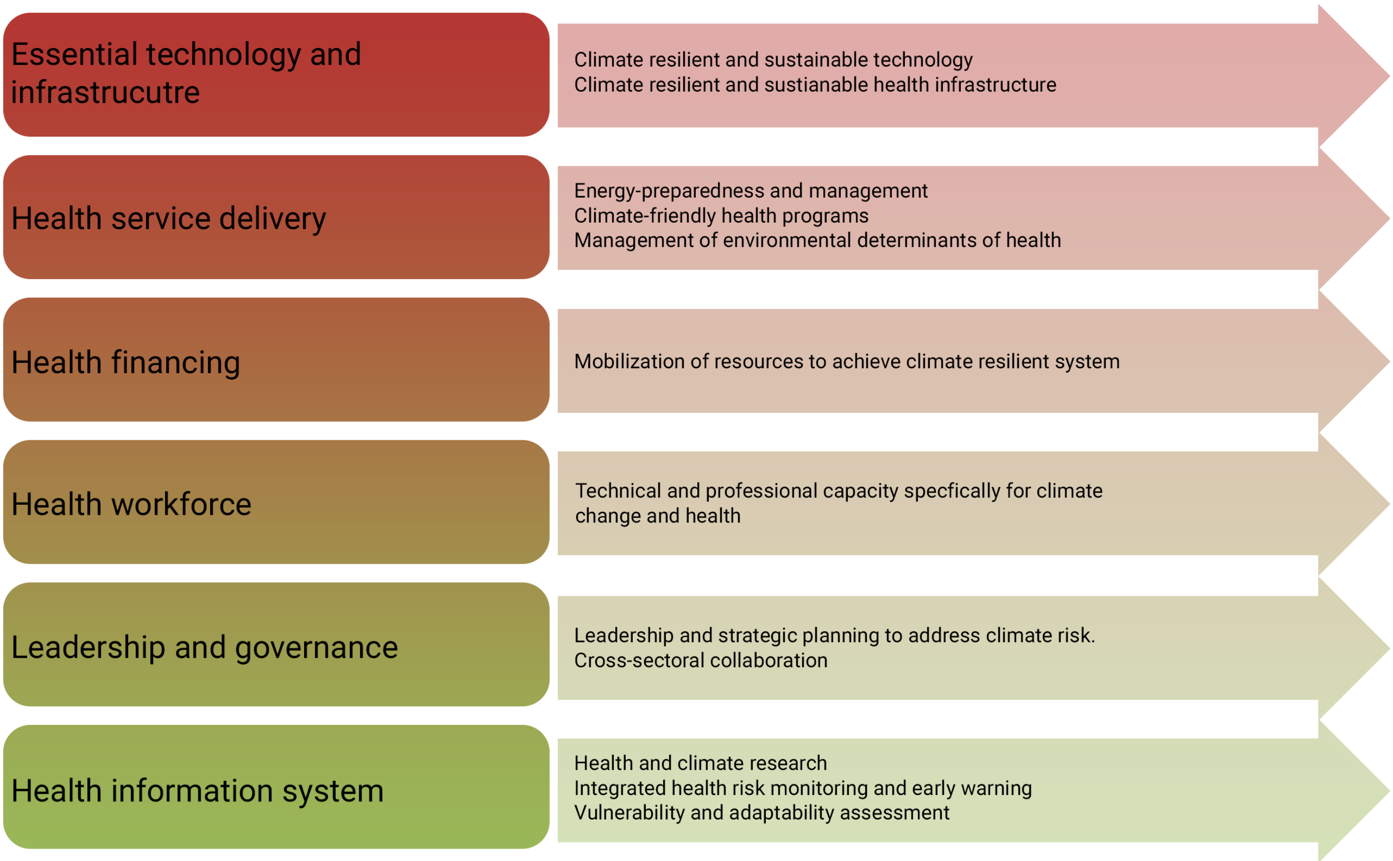

Figure 33. Climate-resilient health system and building blocks of the health system

While all building blocks should be considered in system-wide planning, only the vision for climate resilient and environmentally sustainable health facilities is elaborated in the Plan.

Why focus on health facilities? Health facilities are settings which provide healthcare to individuals and communities. They vary in size from a small BHS to big and complex level 3 hospitals, but they all face risks. Health facilities are vulnerable to climate change and environmental stresses. Some of these facilities lack proper infrastructure, sufficient health workforce, and experience inadequate water and energy supply.

Also, health facilities have a negative impact on health and the environment, through emissions of greenhouse gases, which contribute to climate change and through discharges of different kinds of waste to the environment.

The Figure 34 below shows the four (4) areas for interventions in achieving climate resilient and

environmentally sustainable health infrastructure. Under each area, the Plan identified essential features of all modern and future health facilities in the country. Investing in these features have two primary goals: (1) improve climate resilience and (2) environmental sustainability. These features were adopted from the WHO's guidance of climate and resilient environmentally sustainable health infrastructures.

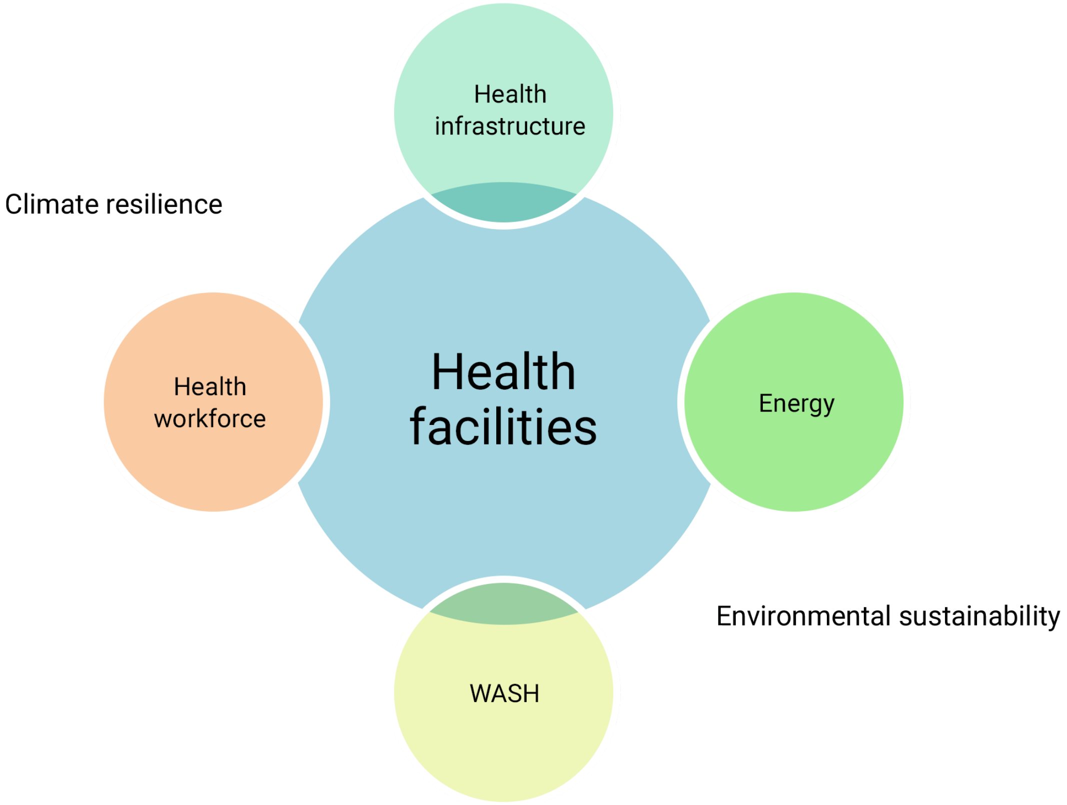

Figure 34. Conceptual framework: Building climate-resilient and environmentally sustainable health care facilities

Health infrastructure

Health infrastructure is composed of structural and non-structural components. Structural components are load-bearing components that make a building stand including foundations, footings, columns and beams to resist gravity, earthquakes, wind, floor, and other pressures. Non-structural components are on-load bearing features and contents of the building, such as walls, divisions, partitions, windows, doors, ceilings, and floor finishing. These are components of the building that enable and support the adequate functioning of a facility and, systems, procedures, and protocols to enable a hospital to have capacity to remain functional and operational.

Climate resilience	Environmental sustainability
• Healthcare facilities implement building regulations that promote climate resilience. For example, assessment of health facilities for hazards, regular assessment for signs of deterioration of structural components, and assessment of safety after extreme weather events or disasters).<br>• Healthcare facilities adopt new technologies and processes that could provide climate resilience and enhanced health service delivery (that is, availability of national and local warning signs, health IT system with climate information to provide information for early intervention, availability of reliable backup communication systems, use of proven smart materials and applications, sensors, low power electronics and equipment)<br>• Healthcare facilities adopt and procure technologies and to support continuous hospital operations. For example, availability of emergency room surge capacity, availability of medicines to cover surge can sustain the provision, availability of stockpile of essential supplies and pharmaceuticals in accordance with national guidelines, availability of backup food and water sources, and other contingency protocols and measures.	• Healthcare facilities implement building regulations that promote environmental sustainability and energy efficiency. In constructing and retrofitting healthcare facilities, low carbon approaches should be used in the design. For example, the facility should be structured and equipped with air pollution filters to improve indoor air quality, and the corridors should be designed with exterior walls to maximize use of daylight and natural ventilation.<br>• Healthcare facilities adopt new technologies and processes that promote environmental sustainability. For example, use of renewable energy sources such as solar panels and winds, use of clean and renewable energy sources (such as solar panels, wind turbines and biofuel, use of solar panels in hospital parking, use of modern equipment that are energy saving.<br>• Healthcare facilities should institutionalize business operations that is environmentally sustainable. For example, implement a clear environmentally sustainable procurement policy for all types of products, prioritize the purchase of medical equipment and medical devices and supplies that are environmentally-friendly, and promote local and sustainable food production.

Note: See WHO's guidance of climate and resilient environmentally sustainable health infrastructures for detailed interventions.

Energy

Many health care facilities, particularly those in far-flung areas, lack reliable electricity supply needed to power essential services, including communications and medical equipment. Weather disturbances such typhoon could destroy power lines; floods may affect backup generators.

Inefficient use of energy contributes to higher costs and adds to air pollution. In the medium to long-term, the country envisions that all health facilities invest in climate resilient approaches and sustainable energy sources.

Climate resilience	Environmental sustainability
• Healthcare facilities have the capacity to assess their energy use and practices to inform appropriate action. For example, assessment of energy needs and alternative sources and assessment of ventilation and air-conditioning.<br>• Healthcare facilities have the capacity in assessing hazards, and identifying and reducing risks and vulnerabilities. For example, availability of plans for managing intermittent energy failure, and availability of maintenance plans for preventable energy problems.<br>• Healthcare facilities implement regulations and plans on energy use particularly during emergency situations to reduce disruptions during disasters. For example, the availability and functionality of emergency electricity generators and backup energy source if system fails, availability of lighting, communications, and refrigeration equipment if system fails.	• Healthcare facilities have the capacity to determine the efficiency of energy use, and identify areas where energy can be reduced.<br>• Healthcare facilities have the capacity to implement efficient energy solutions that is environmentally sustainable. For example, use of energy efficient LED lighting, occupancy sensor switches, use of design features that maximize natural lighting.<br>• Healthcare facilities implement regulations and policies aim to use energy efficiently. For example, education and awareness campaign to health staff, develop incentive plans to reduce energy consumption, and implementation of energy saving plans in each hospital department.

Note: See WHO's guidance of climate and resilient environmentally sustainable health infrastructures for detailed interventions.

Water, sanitation, chemical and health care waste

The availability of sustainable water, sanitation and environmental, chemical and health care waste management services are essential to quality of care and infection prevention and control in health care facilities.

Climate resilience	Environmental sustainability
• Healthcare facilities have the capacity to monitor and assess the water, sanitation, chemical and healthcare and waste situations of the facility. For example, health facilities were assessed and have all the elements of water distribution system (tank, pipes, and valves) are safe and functional, have a regular monitoring system of water quality, etc<br>• Healthcare facilities have the capacity to implement risk management strategies when water supply fails. For examples, availability of water management plan, including alternative sources during emergency, availability of water storage tank that is built and free from contamination, availability of sanitation and sewer management plan.	• Healthcare facilities should institutionalize business operations and practices that reduce water consumption and waste, and reduce carbon emission. For example, establishment of water recycling facility, establishment of recycling program for non-hazardous waste, phase out incineration of medical waste, and use non-burn technologies.

Note: See WHO's guidance of climate and resilient environmentally sustainable health infrastructures for detailed interventions.

CHAPTER III

Approach in Estimating the Need for Health Facilities

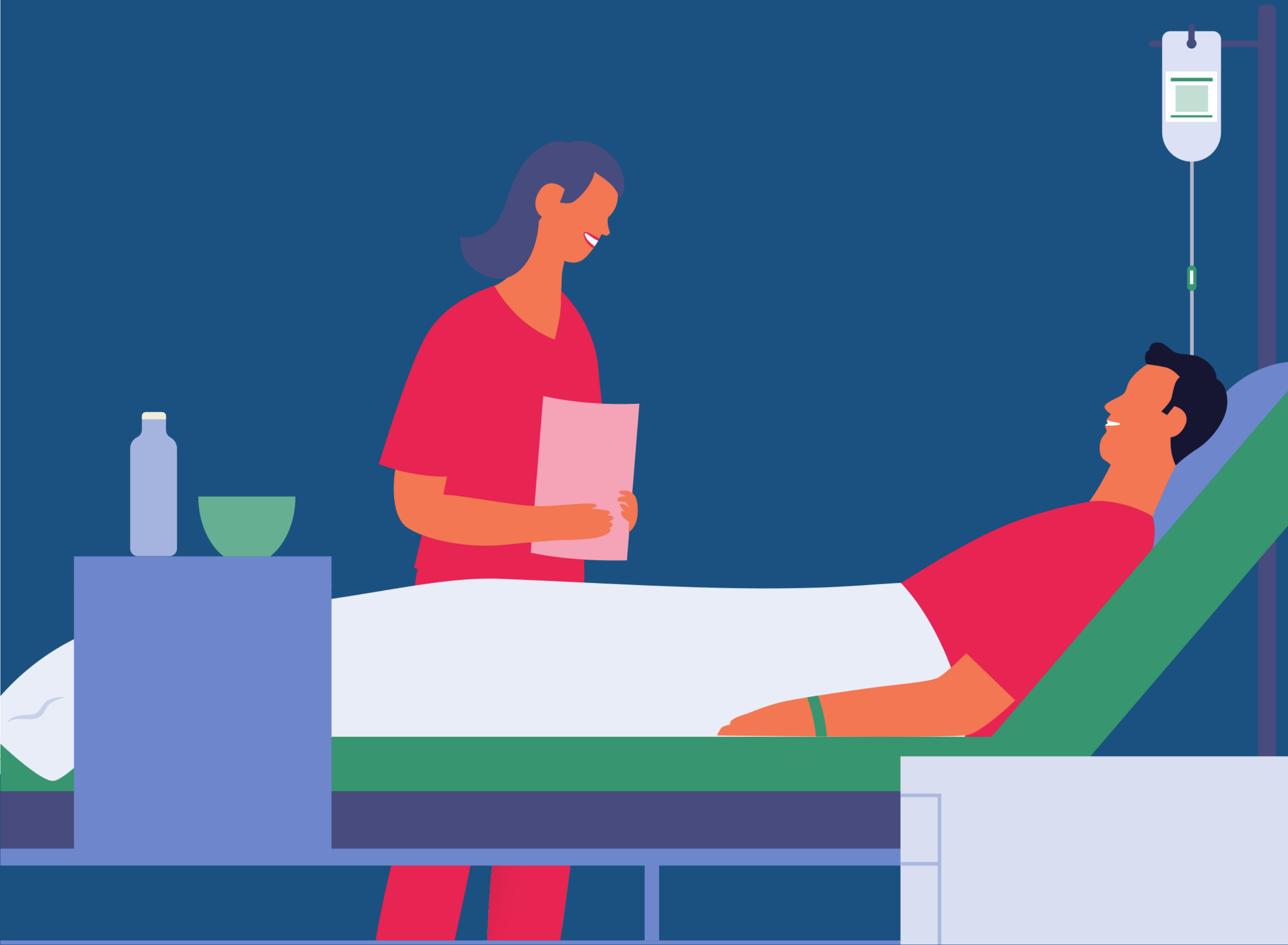

The PHFDP 2020-2040 estimates the health facility requirements for UHC using a needs-based approach. Estimation is done in the context of the envisioned integrated health care provider networks with functioning referral systems. Figure 35 summarizes the steps in estimating the need and gap in health facilities and medical equipment presented from 2022 to 2040.

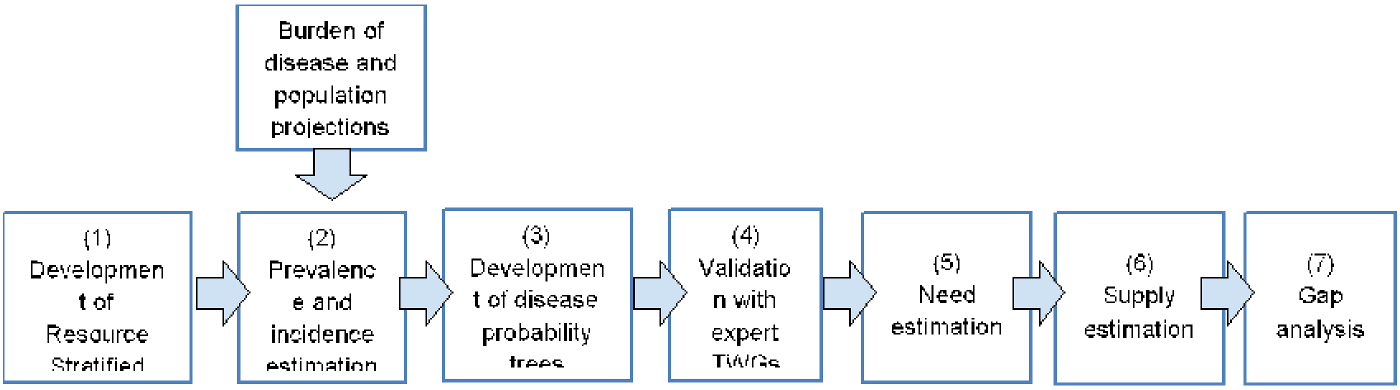

Figure 35. Steps in estimating need for health facilities

A. Development of Resource Stratified Frameworks (RSF)

DOH developed Resource Stratified Frameworks (RSF) for sixteen (16) medical specialties (Figure 36). RSFs delineate roles, minimum service capabilities, resource requirements, and accountabilities for facilities in each level of care in the HCPN. The goal of stratification of capital assets, equipment, and human resources is to foster improved coordination and collaboration across facilities in HCPNs. Likewise, it is intended to rationalize health sector investments to increase access to healthcare, especially by those with the greatest needs.

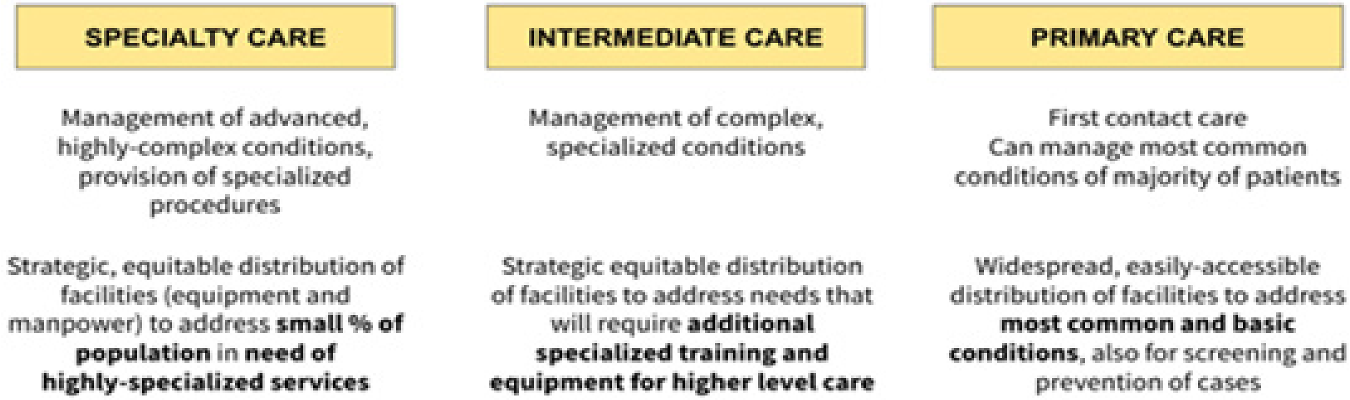

Figure 36. Resource Stratified Framework - Defining services across the Continuum of Care

RSFs were designed in partnership with DOH Hospitals and other health facilities in the second half of 2019 through Technical Working Groups (TWG) composed of leading medical specialists and facility managers. These RSFs were further validated via stakeholder consultations with regional

Centers for Health Development, DOH-retained hospitals, local government units, PhilHealth, government facilities, medical societies, and relevant private institutions.

B. Projecting the Burden of Disease

The prevalence and incidence rates of twenty-five (25) diseases were projected using a multiple regression model. Predictors in the model include human capital (secondary education and per capita income) and technological change (year) (Murray, 2005). The prevalent and incident cases for 2020 to 2040 were estimated by multiplying the projected rates with the population for the same time period from the Philippine Statistics Authority (2019).

The 25 diseases accounted for about 63% of the total burden of disease in the Philippines. The historical incidence and prevalence of 25 diseases was obtained from the following various sources: Institute for Health Metrics for Evaluation (IHME) 2017 Global Burden of Disease Study, Department of Health Surveillance data and National Nutrition Survey. The choice of diseases was based on the top burden of disease, significant laws passed in recent years, and commitment to special needs such as the Millennium and Sustainable Development Goals (SDG).

Table 3. List of the 25 causes of morbidity and mortality, Philippines, 2017

Cause	Share of total DALYs	Cause	Share of total DALYs
Ischemic heart disease	7.93%	Diarrheal diseases	1.63%
Neonatal disorders	7.54%	Depressive disorders	1.13%
Lower respiratory infections	7.08%	Breast cancer	1.02%
Stroke	6.06%	Lung cancer	1.01%
Diabetes mellitus	3.92%	HIV/AIDS	0.80%
Tuberculosis	3.40%	Colon and rectum cancer	0.75%
Congenital birth defects	3.40%	Dengue	0.70%
Chronic kidney disease	3.31%	Drug use disorders	0.53%
Low back pain	2.93%	Malnutrition	0.49%
Chronic obstructive pulmonary disease	2.90%	Maternal disorders	0.38%
Road injuries	1.98%	Appendicitis	0.08%
Asthma	1.92%	Malaria	0.02%
Hypertensive heart disease	1.74%		

Source: Institute for Health Metrics and Evaluation, 2017 Global Burden of Disease

C. Development of Disease Models and Probability Trees

For each disease or condition, a model that represents major health states with significant or distinct resource consumptions for outpatient visits, inpatient bed days, and equipment use at the appropriate level of care was developed. Probability tree diagrams were drafted for each disease model and resource consumptions for major disease management were determined using the RSFs, clinical practice guidelines, literature on past modelling studies, and clinical experience. The lowest possible level of care was identified for outpatient and inpatient consumptions using the RSFs and following the gatekeeping principles of HCPNs under UHC. Draft disease models and resource consumptions were finalized through consultation with relevant specialty TWGs last February 2020.

D. Estimation of Need for Health Facilities and Equipment

The need for outpatient, inpatient, equipment, and national specialty center resources were estimated for each disease for each province, HUC and ICC for 2020 to 2040.

The probabilities for health states were determined by an epidemiologist through a review of literature. Priority was given to studies from the Philippines, then Southeast Asia, then South Asia, and lastly Western countries. The projected disease burden and health state probabilities were used to calculate the resource consumptions in terms of outpatient visits, inpatient bed-days, and equipment use. These numbers were converted to numbers of outpatient physicians, inpatient beds, and machines using the formulas and assumptions in Table 4.

Table 4. Assumptions in estimating the need for health facilities

Facility	Formula and Assumptions
BHS	1 BHS :1 barangay based on the Local Government Code (1991)
PCF	# PCF needed = (% of population without access to RHU/HC within 30 minutes of travel) * population size) / 20,000<br><br>Quantum GIS (QGIS) was used to obtain zonal statistics of the population per province/HUC with access to an RHU/HC within 30 minutes. The data sources were the 2020 administrative shapefiles of the National Mapping and Resource Information Authority (NAMRIA) and the 2020 population estimate from the WorldPop program.<br>AccessMod 5.0 implemented accessibility analyses by considering land cover, elevation, barriers (i.e. inland waters), road networks, travel speeds and the GPS coordinates of the health facilities with the scenarios: walking (5kph), cycling (15kph), motorized vehicle (40kph).
Outpatient: PCP (GP)	# Physicians<br><br>= # consults in a year / # consults one full time physician can do in a year<br># Consults one full time physician can do in a year<br>= (Working days in a year) * (# minutes for consults per day) / # minutes per consult<br><br>PCF services are pegged to physicians and no other types of health staff.<br>Assumed 264 working days a year, 420 minutes per day for consults, 25 minutes per consult.

Inpatient: L1, L2, L3 hospitals	# Beds = # inpatient days for year / (365.25 bed days per bed per year) * (% occupancy) Assumed beds are available all year and that the bed occupancy rate is 80% (global acceptable benchmark).
Equipment	Number of machines (that is X-ray, CT-scan, MRI)<br>= # uses in year / (365.25 days of operation * # uses per day)<br>Number of uses per day = Minutes of operation per day / # minutes per session<br><br>Assumed that machines are operational all days in a year, 24 hours a day.<br>Assumed that existing machines will be replaced once useful life is over.<br>Assumed the following about the number of minutes of use for each machine:

CHAPTER IV

The Need for Health Facilities

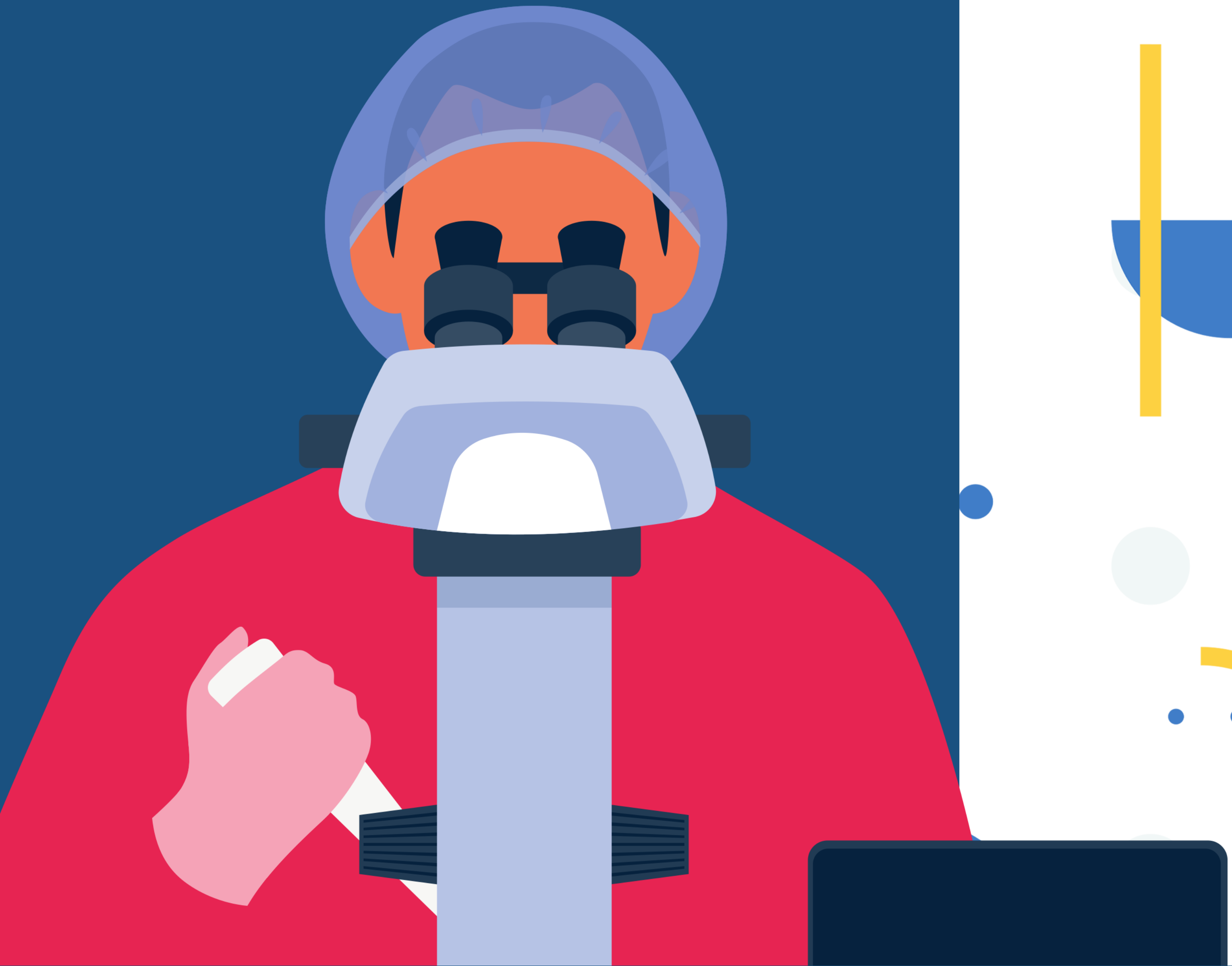

This chapter presents the projected need and supply gaps in health facilities and medical equipment over the next twenty (20) years. The need for health facilities and equipment were based on the expected outpatient consultations and inpatient bed-days.

Outpatient consultations and hospitalization are expected to increase because of rapid population growth and changing disease patterns. NCDs will be the major driver of outpatient primary care and hospitalization visits (see Figure 37 and 38). Table 5 shows projected outpatient visits, inpatient bed-days, and equipment use from 2022 to 2040.

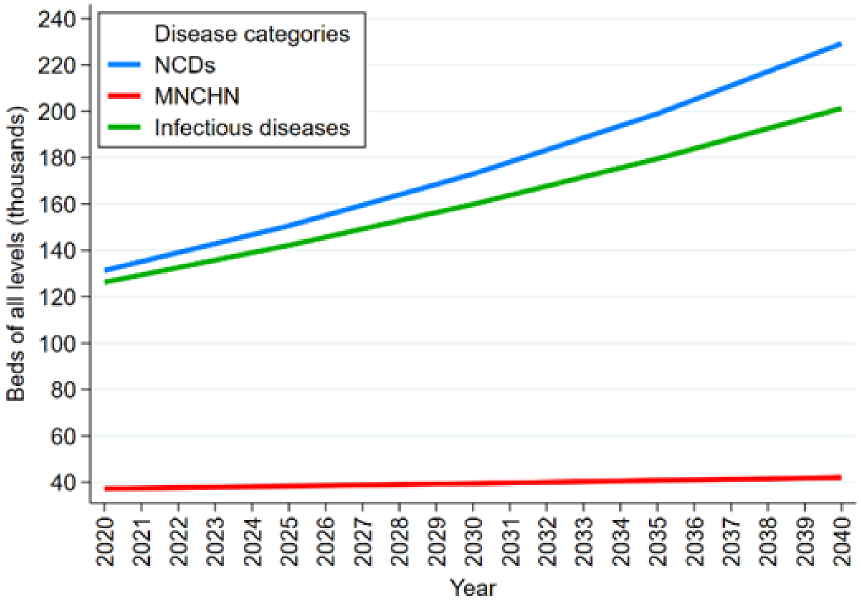

Figure 37. Projected bed-days for all hospital levels, by disease category

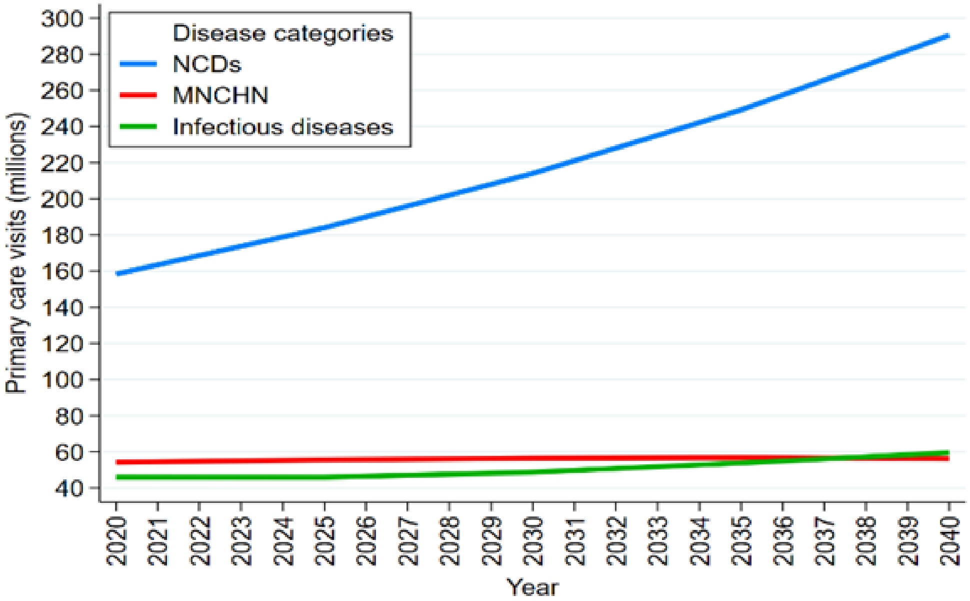

Figure 38. Projected outpatient primary care visits, by disease category

Table 5. Projected number of primary care consultations, inpatient visits, and equipment use

	2020	2025	2030	2035	2040
Estimated PCF outpatient consultations (in thousands)	263,069	290,853	325,491	366,969	414,296
Estimated inpatient bed days (in thousands)					
Level 1	171	191	214	240	269
Level 2	69	77	85	95	107
Level 3/Apex	65	76	87	100	114
Estimated number of uses (in thousands)					
X-ray	119,945	134,994	152,226	172,010	194,785
MRI	1,358	1,547	1,764	2,016	2,308
CT-scan	6,031	6,849	7,763	8,782	9,916
Ultrasound	58,084	66,645	76,403	87,490	100,117
ECG	31,095	36,426	42,534	49,498	57,471
LINAC	1,544	1,878	2,246	2,648	3,083
Peritoneal Dialysis	476	552	639	739	853
Hemodialysis	301	349	404	467	539

A. The Need for Health Stations and Primary Care Facilities

Primary care facilities serve as the entry point of communities into the HCPN. The Local Government Code mandates that all barangays should have at least one (1) BHS, but about 50% of the total barangays in the country do not have a BHS. Table 6 shows the number of barangays and the number of BHS by region. Ideally, the BHS to barangay ratio should be ≥1.

Table 6. Number of barangay health stations and barangays

Region	BHS	Barangays	Share (BHS/ barangay)	Estimated gap
**Philippines**	22,613	42,045	0.5	19,095
**NCR**	22	1,710	0.0	1,114
**CAR**	918	1,177	0.8	304
**I - Ilocos**	1,791	3,267	0.5	1,476
**II - Cagayan**	1,427	2,311	0.6	884
**III – Central Luzon**	2,063	3,102	0.7	1,039
**IVA - CALABARZON**	2,801	4,019	0.7	1,310
**IVB – MIMAROPA**	1,155	1,460	0.8	305
**V - Bicol**	1,497	3,471	0.4	1,974
**VI. Western Visayas**	1,971	4,051	0.5	2,109
**VII - Central Visayas**	2,254	3,003	0.8	758
**VIII - Eastern Visayas**	927	4,390	0.2	3,463

IX - Zamboanga Peninsula	753	1,859	0.4	1,106
X - Northern Mindanao	1,238	2,022	0.6	787
XI – Davao Region	1,190	1,162	1.0	8
XII – SOCCSKSARGEN	1,172	1,195	1.0	46
XIII – CARAGA	784	1,311	0.6	527
BARMM	650	2,535	0.3	1,885

Source: National Health Facility Registry 2019. Some Provinces and HUCs/ ICCS have more BHS than barangay hence gaps were converted to zero. The National Health Facility Registry 2019 only contains 22 BHS for NCR.

There are approximately 4,000 PCFs in the country, of which more than half are government owned (i.e., RHUs or HCs). Based on access analysis, around 53% of the population do not have access to an RHU/ HC within 30 minutes.

Table 7. Supply and need for primary care facilities

Region	Govern-ment	Private	Current supply (2019) (a)	Project-ed need for PCF in 2025 (b)	Projected additional need for PCF (c)			Cumu-lative projected need for PCF 2025-2040 (d)
					2030	2035	2040	
Philippines	2,593	1,265	3,858	6,258	182	207	215	6,862
NCR	495	351	846	849	-	-	-	849
CAR	101	48	149	188	1	3	4	196
I - Ilocos	152	55	207	323	6	9	7	345
II - Cagayan	96	52	148	288	9	9	11	317
III – Central Luzon	283	214	497	679	16	17	18	730
IVA - CALABARZON	228	116	344	475	11	12	13	511
IVB – MIMAROPA	81	28	109	242	11	12	13	278
V - Bicol	129	50	179	418	15	19	18	470
VI. Western Visayas	145	104	249	482	14	17	14	527
VII - Central Visayas	160	58	218	373	11	13	13	410
VIII - Eastern Visayas	168	48	216	381	9	11	13	414
IX - Zamboanga Peninsula	94	12	106	221	9	9	10	250
X - Northern Mindanao	120	30	150	285	15	13	15	328
XI – Davao Region	68	22	90	252	14	15	17	298
XII – SOCCSKSARGEN	62	31	93	260	17	20	20	317
XIII – CARAGA	81	16	97	206	8	8	9	231
BARMM	130	30	160	336	16	19	20	391

a - Current supply of Primary Care Facilities including RHU, HC and Private Medical Outpatient Clinics as of 2019 (Source: National Health Facility Registry and 2019)
b - Projected total need for PCF by 2025 to meet population demand
c - Projected additional need for 2030, 2035 and 2040 to meet population demand for the given year
d - Cumulative projected need from 2025-2040

The ideal approach in estimating the need for primary care should be in terms of primary care physicians (PCPs) and not by the sole presence of a health facility. Based on the staffing requirements of the DOH, primary care facilities (PCFs) should be staffed with at least one primary care physician (PCP). A PCF can employ more than one PCP to meet the population need.

The country needs around 65,000 physicians (0.52 per 1000 population) to meet the primary care needs of the population. This estimated density of PCP is quite ambitious; it is comparable to countries with robust primary care systems in advanced economies (that is United Kingdom). Table 9 shows the number of PCP needed by the population. Deeper analysis of health workforce needs shall be made available in a separate Human Resources for Health (HRH) Master Plan.

Table 8. Need for outpatient primary care physicians

Region	Projected need for PCP in 2025	Projected additional need for PCP			Cumulative projected need for PCP 2025-2040
		2030	2035	2040	
Philippines	65,578	7,810	9,352	10,671	93,411
NCR	7,702	816	916	1,029	10,463
CAR	1,059	120	136	154	1,469
I - Ilocos	3,000	182	262	295	3,739
II - Cagayan	2,201	227	262	227	2,917
III – Central Luzon	7,376	855	824	576	9,631
IVA - CALABARZON	10,179	1,623	2,065	2,528	16,395
IVB – MIMAROPA	1,912	146	247	277	2,582
V - Bicol	3,521	284	354	380	4,539
VI. Western Visayas	4,765	298	303	420	5,786
VII - Central Visayas	4,817	569	674	799	6,859
VIII - Eastern Visayas	2,730	257	325	408	3,720
IX - Zamboanga Peninsula	2,488	396	480	574	3,938
X - Northern Mindanao	3,017	415	466	525	4,423
XI – Davao Region	3,108	340	444	500	4,392
XII – SOCCSKSARGEN	3,409	667	878	1,141	6,095
XIII – CARAGA	1,672	173	205	244	2,294
BARMM	2,624	440	511	593	4,168

B. The Need for Hospital Care

The country has 105,000 hospital beds (or 1.2 per 1,000 population). To meet the health needs of the population, the current supply of hospital beds must increase to 2.7 per 1,000 population. The estimated need for hospital beds is still lower than the average bed to population ratio for upper-middle- and high-income countries (3.8 beds and 4.1 per 1,000, respectively) (see Figure 39). This

gap in beds may be addressed by building new hospitals or by expanding existing hospitals or infirmaries. Infirmaries are most suited for upgrading as base infrastructure already exists and many are located in low-resource areas where the need to expand access is most critical. However, this has to be matched with other resource requirements such as adequate financing, human resources and other operational needs.

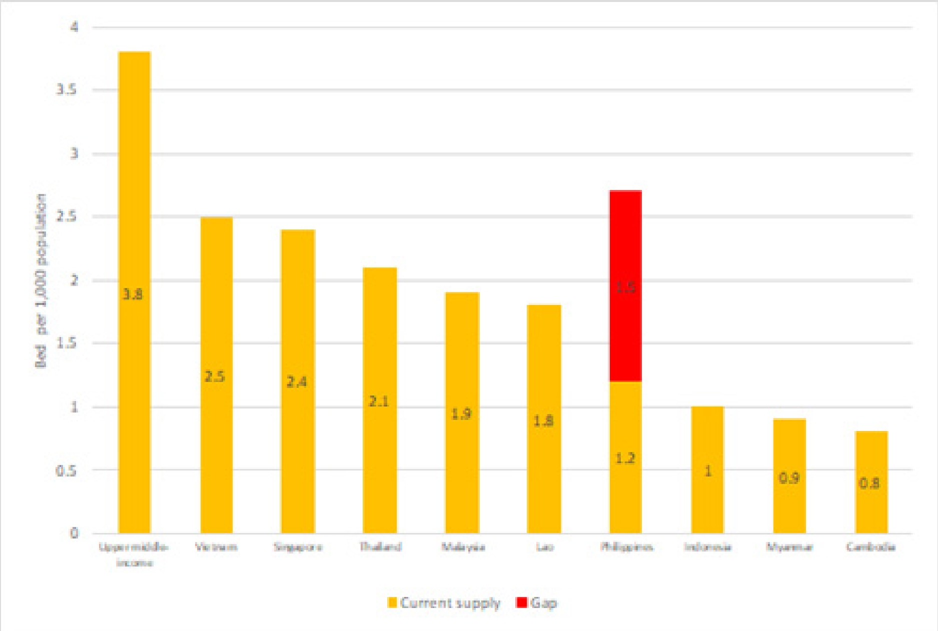

Figure 39. Bed-to-population ratio (per 1000), selected comparator countries

Source: Raw data for current supply (yellow bar) from World Bank

Tables 9-11 show the supply and need for hospital beds, by level. The Philippines, in general, needs to expand the number of hospital beds. However, Level 1 hospitals should be prioritized as it appears to have the largest gap.

Table 9. Supply and need for Level 1 hospital beds

Region	Current supply (2019) (a)	Projected need for L1 beds in 2025 (b)	Projected additional need for L1 bed (c)			Cumulative Projected need for L1 beds in 2025-2040 (d)
			2030	2035	2040	
Philippines	31,494	191,218	22,909	25,793	29,055	268,975
NCR	3,135	23,548	2,544	2,849	3,189	32,130
CAR	803	3,167	328	362	402	4,259
I - Ilocos	1,597	8,784	732	787	849	11,152
II - Cagayan	1,913	6,234	571	624	675	8,104
III – Central Luzon	2,901	21,309	2,599	2,902	3,244	30,054
IVA - CALABARZON	4,755	29,650	4,993	5,851	6,851	47,345
IVB – MIMAROPA	1,269	5,569	612	684	762	7,627
V - Bicol	1,666	10,609	979	1,059	1,147	13,794

VI - Western Visayas	2,007	13,467	1,211	1,308	1,407	17,393
VII - Central Visayas	1,488	13,982	1,608	1,801	2,021	19,412
VIII - Eastern Visayas	1,789	8,012	749	832	931	10,524
IX - Zamboanga Peninsula	1,297	6,644	718	785	854	9,001
X - Northern Mindanao	1,923	9,060	1,126	1,258	1,404	12,848
XI – Davao Region	1,196	9,339	1,135	1,264	1,406	13,144
XII – SOCCSKSARGEN	2,347	9,131	1,321	1,510	1,725	13,687
XIII – CARAGA	567	4,785	476	526	585	6,372
BARMM	841	7,928	1,207	1,391	1,603	12,129

Note: Military hospital beds are excluded in the current supply as they are not for general public use.

a - Current supply of beds as of 2019 (Source: National Health Facility Registry and 2019)
b - Projected total need by 2025 to meet population demand
c - Projected additional need for 2030, 2035 and 2040 to meet population demand for the given year
d - Cumulative projected need from 2025-2040

Table 10. Supply and need for Level 2 hospital beds

Region	Current supply (2019) (a)	Projected need for L2 beds in 2025 (b)	Projected additional need for L2 bed (c)			Cumulative Projected need for L2 beds in 2025-2040 (d)
			2030	2035	2040	
Philippines	32,063	76,581	8,531	10,143	12,140	107,395
NCR	2,725	9,338	940	1,123	1,351	12,752
CAR	534	1,258	116	137	162	1,673
I - Ilocos	1,653	3,545	268	315	368	4,496
II - Cagayan	1,120	2,481	203	242	284	3,210
III – Central Luzon	4,000	8,504	995	1,179	1,401	12,079
IVA - CALABARZON	6,589	11,749	1,839	2,240	2,745	18,573
IVB – MIMAROPA	307	2,235	223	263	313	3,034
V - Bicol	1,274	4,266	358	418	495	5,537
VI - Western Visayas	1,972	5,378	440	511	602	6,931
VII - Central Visayas	1,859	5,639	596	708	840	7,783
VIII - Eastern Visayas	692	3,241	272	317	372	4,202
IX - Zamboanga Peninsula	1,062	2,684	275	321	379	3,659
X - Northern Mindanao	2,710	3,640	419	492	582	5,133
XI – Davao Region	2,114	3,767	428	502	593	5,290
XII – SOCCSKSARGEN	1,929	3,670	490	579	695	5,434
XIII – CARAGA	2,725	9,338	940	1,123	1,351	12,752
BARMM	1,085	1,921	166	191	228	2,506

Note: Military hospital beds are excluded in the current supply as they are not for general public use.

a - Current supply of beds as of 2019 (Source: National Health Facility Registry and 2019)
b - Projected total need by 2025 to meet population demand
c - Projected additional need for 2030, 2035 and 2040 to meet population demand for the given year
d - Cumulative projected need from 2025-2040

Table 11. Supply and need for Level 3/ Apex hospital beds

Region	Supply of L3 beds (2019) (a)	Need for L3 beds in 2025 (b)	Projected additional need for L3/ Apex beds (c)			Cumulative Need for L3 beds in 2025-2040 (d)
			2030	2035	2040	
Philippines	31,043	75,475	11,726	12,913	14,119	114,233
NCR	13,140	10,876	1,857	1,946	1,985	16,664
CAR	680	1,200	154	165	179	1,698
I - Ilocos	1,000	3,391	363	380	393	4,527
II - Cagayan	806	2,351	274	294	313	3,232
III – Central Luzon	2,094	8,468	1,417	1,596	1,793	13,274
IVA - CALABARZON	1,702	11,841	2,534	2,985	3,503	20,863
IVB – MIMAROPA	0	2,084	290	325	362	3,061
V - Bicol	850	3,939	454	485	515	5,393
VI - Western Visayas	2,446	5,265	674	734	799	7,472
VII - Central Visayas	2,783	5,611	803	802	765	7,981
VIII - Eastern Visayas	1,129	3,010	360	396	438	4,204
IX - Zamboanga Peninsula	400	2,447	312	338	363	3,460
X - Northern Mindanao	560	3,376	499	553	611	5,039
XI – Davao Region	2,630	3,721	610	680	758	5,769
XII – SOCCSKSARGEN	823	3,390	554	620	690	5,254
XIII – CARAGA	0	1,783	216	232	249	2,480
BARMM	0	2,722	356	381	402	3,861

Note: Military hospital beds are excluded in the current supply as they are not for general public use.

a - Current supply of beds as of 2019 (Source: National Health Facility Registry and 2019)
b - Projected total need by 2025 to meet population demand
c - Projected additional need for 2030, 2035 and 2040 to meet population demand for the given year
d - Cumulative projected need from 2025-2040

C. The Need for Medical Equipment

The supply of medical equipment for diagnostics and treatment is scarce relative to need. Tables 13-16 show the supply and need for X-ray, CT scan, MRI, and LINAC equipment. The estimated need significantly exceeds the current supply. Most of the equipment is concentrated in the National Capital Region (NCR). The number of CT scan, MRI, and LINAC equipment in NCR exceeds the estimated need in the region. Ideally, X-ray machines should be available in each province or city-wide HCPN while the need and supply of MRI, CT scan and LINAC may be shared by multiple HCPNs.

Table 12. Supply and need for X-ray machines

Region	Supply of X-ray (2019) (a)	Need for X-ray in 2025 (b)	Projected additional need for X-ray machines (c)			Cumulative Need for X-ray in 2025-2040 (d)
			2030	2035	2040	
Philippines	1,112	3,850	491	565	649	5,555
NCR	262	487	60	68	77	692
CAR	18	64	7	8	8	87
I - Ilocos	53	179	17	18	20	234
II - Cagayan	39	125	13	14	15	167
III – Central Luzon	128	433	59	67	78	637
IVA - CALABARZON	161	598	106	126	151	981
IVB – MIMAROPA	15	111	13	15	17	156
V - Bicol	36	212	22	24	26	284
VI - Western Visayas	134	272	27	30	33	362
VII - Central Visayas	76	280	34	39	44	397
VIII - Eastern Visayas	25	161	17	18	21	217
IX - Zamboanga Peninsula	28	131	15	17	19	182
X - Northern Mindanao	24	180	23	26	30	259
XI – Davao Region	61	187	24	28	31	270
XII – SOCCSKSARGEN	31	180	27	30	36	273
XIII – CARAGA	17	95	10	11	13	129
BARMM	4	152	21	25	28	226

a - Current supply as of 2019 (Source: Food and Drug Administration)
b - Projected total need by 2025 to meet population demand
c - Projected additional need for 2030, 2035 and 2040 to meet population demand for the given year
d - Cumulative projected additional need from 2025-2040

Table 13. Supply and need for CT scan machines

Region	Supply of CT scan (2019) (a)	Need for CT scan in 2025 (b)	Projected additional need for CT scan machines (c)			Cumulative Need for CT scan in 2025-2040 (d)
			2030	2035	2040	
Philippines	456	586	78	87	97	848
NCR	83	74	10	11	11	106
CAR	7	10	1	1	1	13
I - Ilocos	26	27	3	3	3	36
II - Cagayan	32	19	2	2	3	26
III – Central Luzon	57	66	10	10	12	98
IVA - CALABARZON	119	91	17	19	23	150
IVB – MIMAROPA	5	17	2	2	3	24
V - Bicol	17	32	4	4	4	44
VI - Western Visayas	25	42	4	4	5	55

VII - Central Visayas	21	43	5	6	6	60
VIII - Eastern Visayas	10	25	2	3	3	33
IX - Zamboanga Peninsula	7	20	2	3	3	28
X - Northern Mindanao	16	27	4	4	4	39
XI – Davao Region	12	28	4	4	5	41
XII – SOCCSKSARGEN	7	27	4	5	6	42
XIII – CARAGA	9	14	2	2	2	20
BARMM	3	23	3	3	4	33

a - Current supply as of 2019 (Source: Food and Drug Administration)
b - Projected total need by 2025 to meet population demand
c - Projected additional need for 2030, 2035 and 2040 to meet population demand for the given year
d - Cumulative projected need from 2025-2040

Table 14. Supply and need for MRI machines

Region	Supply of MRI (2019) (a)	Need for MRI in 2025 (b)	Projected additional need for MRI machines (c)			Cumulative Need for MRI in 2025-2040 (d)
			2030	2035	2040	
Philippines	109	176	25	29	33	263
NCR	34	22	3	4	4	33
CAR	2	3	0	1	0	4
I - Ilocos	5	8	1	1	1	11
II - Cagayan	4	6	0	1	1	8
III – Central Luzon	13	20	3	3	4	30
IVA - CALABARZON	18	27	6	6	7	46
IVB – MIMAROPA	0	5	1	1	0	7
V - Bicol	2	10	1	1	2	14
VI - Western Visayas	9	13	1	1	2	17
VII - Central Visayas	8	13	2	1	3	19
VIII - Eastern Visayas	1	7	1	1	1	10
IX - Zamboanga Peninsula	1	6	1	1	1	9
X - Northern Mindanao	4	8	1	2	1	12
XI – Davao Region	5	9	1	1	2	13
XII – SOCCSKSARGEN	2	8	2	1	2	13
XIII – CARAGA	1	4	1	0	1	6
BARMM	0	7	1	1	2	11

a - Current supply as of 2019 (Source: Food and Drug Administration)
b - Projected total need by 2025 to meet population demand
c - Projected additional need for 2030, 2035 and 2040 to meet demand for the given year
d - Cumulative projected need from 2025-2040

Table 15. Supply and need for LINAC

Region	Supply of LINAC (2019) (a)	Need for LINAC in 2025 (b)	Projected additional need for LINAC machines (c)			Cumulative Need for LINAC in 2025-2040 (d)
			2030	2035	2040	
**Philippines**	56	214	42	46	50	352
**NCR**	28	27	5	6	6	44
**CAR**	1	4	0	1	1	6
**I - Ilocos**	2	10	2	1	2	15
**II - Cagayan**	1	7	1	1	2	11
**III – Central Luzon**	7	24	5	6	6	41
**IVA - CALABARZON**	5	33	9	9	11	62
**IVB – MIMAROPA**	0	6	1	2	1	10
**V - Bicol**	1	12	2	2	2	18
**VI - Western Visayas**	3	15	3	2	3	23
**VII - Central Visayas**	3	16	2	4	3	25
**VIII - Eastern Visayas**	0	9	2	1	2	14
**IX - Zamboanga Peninsula**	0	7	2	1	2	12
**X - Northern Mindanao**	2	10	2	2	2	16
**XI – Davao Region**	3	10	2	3	2	17
**XII – SOCCSKSARGEN**	0	10	2	3	2	17
**XIII – CARAGA**	0	5	1	1	1	8
**BARMM**	0	8	2	2	2	14

a - Current supply as of 2019 (Source: Food and Drug Administration)
b - Projected total need by 2025 to meet population demand
c - Projected additional need for 2030, 2035 and 2040 to meet demand for the given year
d - Cumulative projected need from 2025-2040

CHAPTER V

Allocation Framework

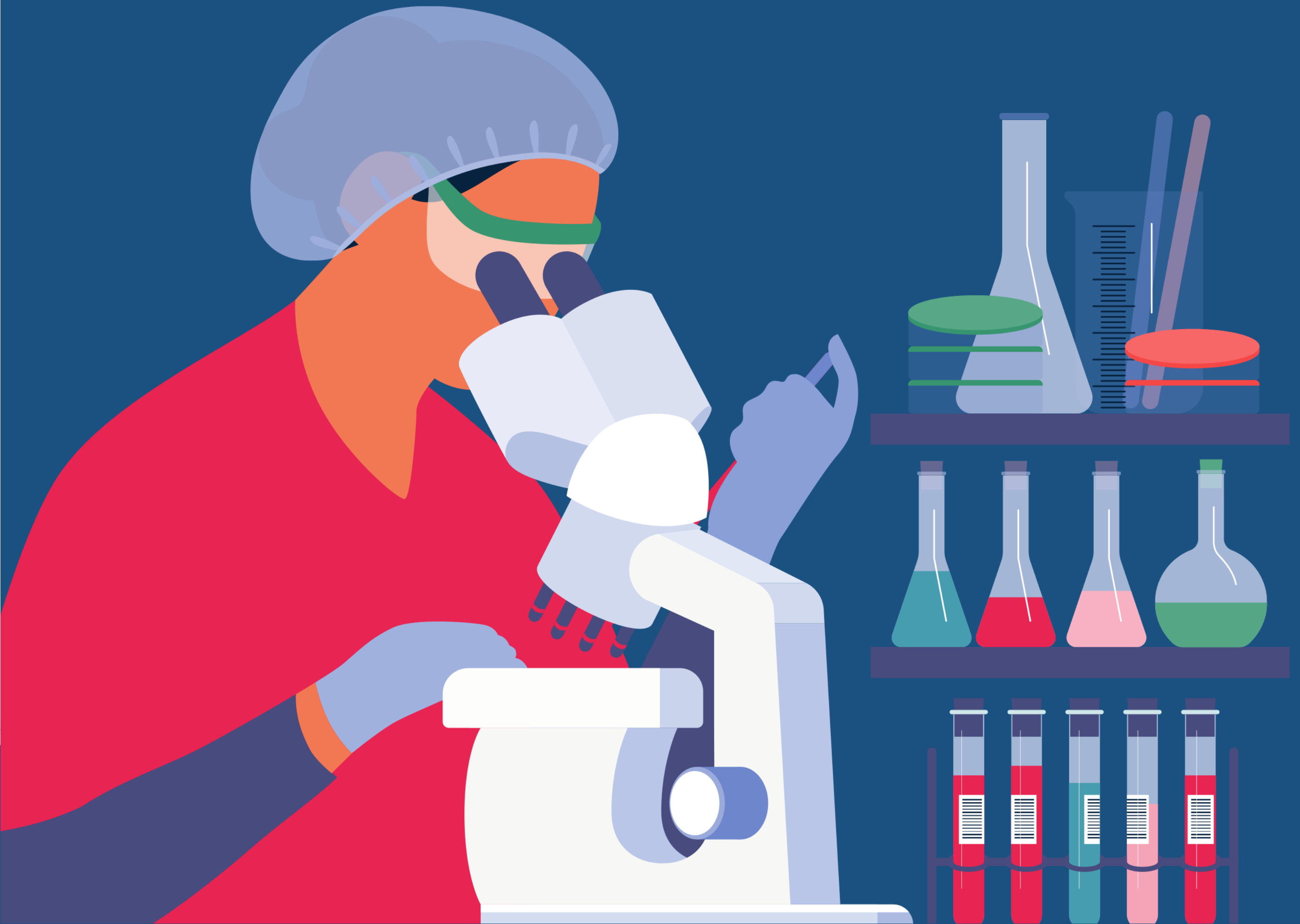

The previous chapter presented the gap in primary care facilities and hospital beds in the next 20 years. But a critical question remains: who will finance the large gap in health facilities?

The first step is to identify the share of the gap to be fulfilled both by the public and private sectors. The Plan proposes the gap in PCF should be financed by the public sector. While the gaps in hospital beds will be shouldered both by the private and public sectors. The share of public and private sector depends on the capacity and the private market of the province and HUCs; the higher the capacity and larger presence of private sector in the area, the higher expected private sector share. Table 16 shows the share of the public and private sectors of the cumulative gap.

Table 16. Estimated cumulative gap in hospital beds until 2040, by public and private

	Level 1			Level 2		
	Private	Public	Share of private	Private	Public	Share of private
Philippines	143,555	93,639	61%	44,100	31,120	59%
NCR	24,646	4,349	85%	8,523	1,504	85%
CAR	1,771	1,685	51%	565	574	50%
I - Ilocos	6,621	2,934	69%	1,992	851	70%
II - Cagayan	3,209	2,982	52%	1,079	1,011	52%
III – Central Luzon	17,571	9,582	65%	5,261	2,818	65%
IVA - CALABARZON	32,209	10,382	76%	8,948	3,036	75%
IVB – MIMAROPA	3,532	2,826	56%	1,470	1,257	54%
V - Bicol	4,860	7,268	40%	1,658	2,605	39%
VI - Western Visayas	8,477	6,909	55%	2,490	2,469	50%
VII - Central Visayas	11,656	6,268	65%	3,685	2,239	62%
VIII - Eastern Visayas	4,017	4,718	46%	1,532	1,978	44%
IX - Zamboanga Peninsula	2,909	4,795	38%	944	1,653	36%
X - Northern Mindanao	4,910	6,015	45%	945	1,478	39%
XI – Davao Region	5,910	6,038	49%	1,530	1,646	48%
XII – SOCCSKSARGEN	4,600	6,740	41%	1,264	2,241	36%
XIII – CARAGA	2,707	2,811	49%	580	730	44%
BARMM	3,951	7,337	35%	1,633	3,032	35%

Based on the projected shares of the private and public sectors, the medium and long-term public spending for capital outlay can be then estimated.

The national government can use a simple decision-making framework to identify priority provinces and HUCs/ ICCs. The Plan uses this framework in the calculation of expected cost of the national and local governments.

- First, identify priority provinces and HUCs/ICCs based on capacity. The Plan proposes three parameters converted into a single score to measure capacity:
    - fh Resources of local government. Public spending is a strong indicator of LGUs capacity

to carry out health infrastructure programs. This indicator is measured using the latest public spending per capita of provinces and HUC/ ICCs (2018).

fh Presence of GIDA. Building health infrastructure in geographically isolated and disadvantaged areas (GIDA) is costly. This indicator is measured by the share of barangays in the province and HUC/ ICCs classified as GIDA

fh Level of household income. Areas with high poverty incidence are expected to have lower private sector penetration. This indicator is measured using poverty incidence, which is the proportion of the population below the poverty line.

- Second, the national government should examine the ‘capacity score’ together with the gap in health facilities. The total gap was measured by adding the min max scores of both PCF and hospital beds (level 1 and level 2). The median score was used as cut-off point to classify high gap and low provinces/HUCs (see Figure 40).

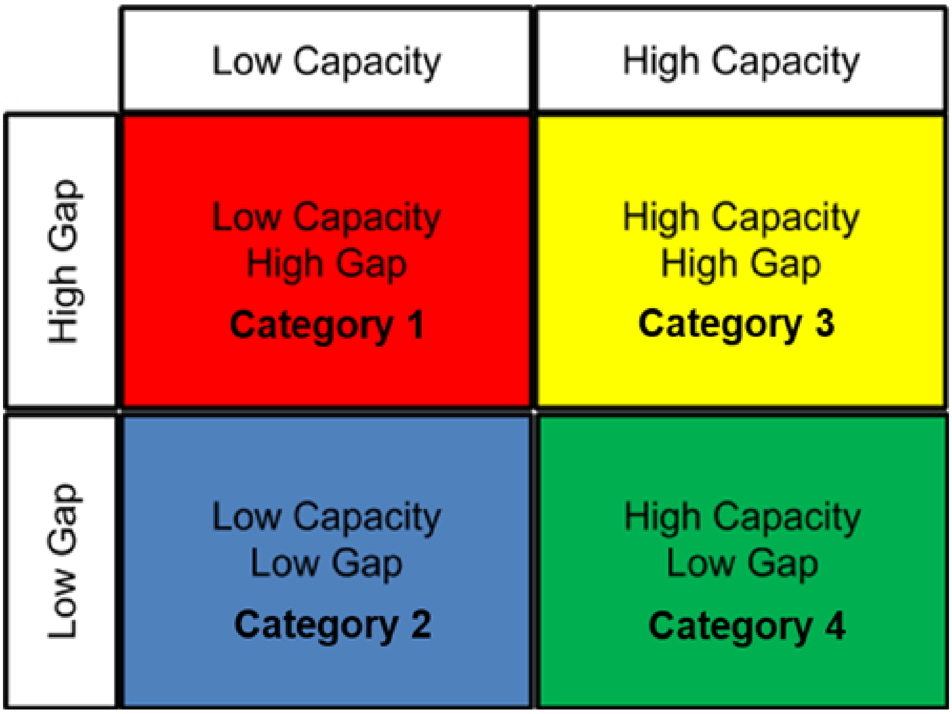

Figure 40. National Allocation Framework

The framework only applies to health facilities with provincial catchment. The Plan uses regional GDP per capita as the only measure of ‘regional capacity’. Regions typically serve as the catchment area for Level 3 hospitals and priority technologies (that is MRI, CT Scan). Table 17 shows the gap in Level 3 hospital beds across regions. Again, from an equity standpoint, the national government should prioritize the poorest regions in the country. BARMM, CARAGA, and MIMAROPA do not have Level 3 hospitals, and a surplus in NCR. As with the other health infrastructure investments above, these facilities and equipment ought to be complemented with the appropriate human resources and operational requirements.

Table 17. Capacity vs. Gap in Level 3 hospital beds and equipment

Region	GDP per capita (as measure of regional capacity)	Category
BARMM	639	Category 1
V – Bicol	1,163	Category 1
XIII – CARAGA	1,334	Category 1
VIII - Eastern Visayas	1,468	Category 1
IVB – MIMAROPA	1,659	Category 1
II – Cagayan	1,650	Category 1
IX - Zamboanga Peninsula	1,714	Category 2
VI - Western Visayas	1,826	Category 2
XII – SOCCSKSARGEN	1,925	Category 2
I – Ilocos	2,040	Category 2
III – Central Luzon	2,774	Category 3
X - Northern Mindanao	2,782	Category 3
VII - Central Visayas	2,938	Category 3
XI – Davao Region	3,088	Category 3
CAR	3,212	Category 3
IVA – CALABARZON	3,419	Category 3
NCR	9,939	Category 3

The third step is to estimate the required public spending in the medium-term (2020-2025). The share of national and local governments in the total public spending is dependent on the category as described in Figure 40 and Table 17. The Plan assumes the following shares:

- Level 1 and level 2 government hospitals:
  - fh Category 1: 100% - national government
  - fh Category 2: 75% - national government; 25% local governments
  - fh Category 3: 50% - national government; 50% local governments
  - fh Category 4: 25% - national government; 75% local governments
- PCF
  - fh Regardless of category as long as there is gap: 50% - national government; 50% local government
- For regional catchment (level 3 hospitals and equipment):
  - fh Category 1: 100% national government
  - fh Category 2: 75% national government; 25% local governments
  - fh Category 3: 25% national government; 75% local governments

Table 18. Public investments by health facility, 2021-2025

	PCF	L1	L2	L3	TOTAL
5- year public spending					
National	5.7	28.6	77.1	31	142.4
Local	5.7	22.8	84.8	22.1	135.4
All	11.5	51.4	161.9	53.1	277.9
Annualized public spending					
National	1.1	5.7	15.4	6.2	28.5
Local	1.1	4.6	17	4.4	27.1
TOTAL by Facility	2.3	10.3	32.4	10.6	55.6

Table 19 shows the medium and long-term gaps in public PCF and public level 1 and 2 hospital beds by province/HUC. Ultimately, the goal of provinces and HUCs is to have a complete set of health facilities necessary to build an HPCN. Decision makers, however, should identify which type of health facility should be prioritize when facing limited resources. The red color means high priority, blue means medium priority, and green means low priority/no priority. The table also includes the classification of provinces/HUC based on national priority. This will guide the national government which provinces should be prioritized or requires higher national government subsidy. The Plan uses a criterion to identify which HPCN facility should be prioritized.

Table 19. Gap and priority facility, by province and HUC

Regional code	UHC sites	National priority	Gap until 2025			2026-2040 (Gap)		
			PCF	L1 beds	L2 beds	PCF	L1 beds	L2 beds
14	Abra	1	4	32	42	0	96	127
16	Agusan del Norte	1	14	81	121	3	243	362
16	Agusan del Sur	2	33	186	256	8	559	767
6	Aklan	1	19	128	230	4	385	689
5	Albay	2	58	255	400	11	764	1199
6	Antique	4	21	81	179	4	242	537
14	Apayao	1	6	15	18	1	44	54
3	Aurora	3	9	28	59	2	83	177
15	Basilan	1	24	153	200	8	460	600
3	Bataan	4	24	119	298	8	357	895
2	Batanes	1	0	-4	-9	0	-13	-26
4	Batangas	4	51	410	999	18	1230	2998
14	Benguet	1	6	105	172	2	316	515
8	Biliran	3	6	20	38	1	60	115
7	Bohol	2	43	212	317	7	635	951
10	Bukidnon	2	62	390	565	19	1171	1696

3	Bulacan	4	0	547	1213	0	1640	3639
3	CITY OF ANGELES	3	0	29	181	0	87	544
6	CITY OF BACOLOD (Capital)	3	20	38	212	6	114	636
14	CITY OF BAGUIO	3	0	59	152	0	178	457
16	CITY OF BUTUAN (Capital)	3	14	43	130	3	128	390
10	CITY OF CAGAYAN DE ORO (Capital)	3	4	110	297	2	331	890
13	CITY OF CALOOCAN	4	0	101	428	0	303	1285
7	CITY OF CEBU (Capital)	4	0	61	317	0	183	951
11	CITY OF DAVAO	4	38	281	665	14	843	1994
12	CITY OF GENERAL SANTOS (DADIANGAS)	3	10	106	303	3	318	909
10	CITY OF ILIGAN	3	5	45	143	1	135	428
6	CITY OF ILOILO (Capital)	3	0	28	178	0	83	535
7	CITY OF LAPU-LAPU (OPON)	4	0	44	190	0	132	570
13	CITY OF LAS PIÑAS	3	0	34	186	0	102	559
4	CITY OF LUCENA (Capital)	3	0	19	181	0	57	543
13	CITY OF MAKATI	4	0	35	165	0	104	494
13	CITY OF MALABON	3	0	17	72	0	50	215
13	CITY OF MANDALUYONG	3	0	26	135	0	77	405
7	CITY OF MANDAUE	3	0	27	220	0	81	659
13	CITY OF MANILA	4	0	73	270	0	218	809
13	CITY OF MARIKINA	3	0	21	118	0	62	353
13	CITY OF MUNTINLUPA	3	0	17	93	0	51	279
13	CITY OF NAVOTAS	3	0	11	46	0	33	137
3	CITY OF OLONGAPO	3	0	23	84	0	69	251
13	CITY OF PARAÑAQUE	4	0	46	235	0	138	704
13	CITY OF PASIG	4	0	55	283	0	166	850
17	CITY OF PUERTO PRINCESA (Capital)	3	11	16	83	5	48	248
13	CITY OF SAN JUAN	3	3	2	-2	0	5	-5
8	CITY OF TACLOBAN (Capital)	3	2	17	105	0	52	316
13	CITY OF TAGUIG/ PATEROS	4	0	75	332	0	225	997
13	CITY OF VALENZUELA	4	0	39	195	0	118	585
9	CITY OF ZAMBOANGA	1	19	204	344	7	613	1031
2	Cagayan	4	51	129	294	10	388	881

5	Camarines Norte	1	21	124	202	4	371	607
5	Camarines Sur	2	70	447	583	16	1341	1748
10	Camiguin	1	3	11	13	1	34	39
6	Capiz	3	25	89	233	4	267	699
5	Catanduanes	1	11	50	88	3	151	264
4	Cavite	4	0	348	1735	0	1044	5204
7	Cebu	4	65	477	1035	21	1430	3106
12	City of Cotabato	1	2	108	167	1	325	501
11	Compostela Valley	2	31	201	264	7.999998	603	792
11	Davao Occidental	1	17	86	113	3	257	338
11	Davao Oriental	2	26	151	204	7	452	611
11	Davao del Norte	2	34	247	414	11	740	1242
11	Davao del Sur	1	16	92	164	3	275	491
16	Dinagat Islands	2	5	.	.	1	.	.
8	Eastern Samar	1	22	94	124	5	283	372
6	Guimaras	1	7	37	54	1	111	163
14	Ifugao	1	9	51	66	2	152	197
1	Ilocos Norte	3	14	29	141	3	86	424
1	Ilocos Sur	3	10	25	127	1	76	382
6	Iloilo	2	52	349	498	11	1047	1495
2	Isabela	2	64	279	432	14	836	1297
14	Kalinga	3	9	20	35	2	61	106
1	La Union	2	14	96	215	3	287	646
4	Laguna	4	0	227	1230	0	681	3691
10	Lanao del Norte	2	22	181	226	9	544	679
15	Lanao del Sur	2	45	329	453	12	986	1360
8	Leyte	2	56	328	484	11	984	1452
15	Maguindanao	2	53	437	569	22	1311	1708
17	Marinduque	3	9	20	36	0	59	107
5	Masbate	2	44	233	304	11	699	912
10	Misamis Occidental	1	20	104	193	5	313	578
10	Misamis Oriental	2	19	210	310	6	631	931
14	Mountain Province	1	5	13	20	1	39	61
6	Negros Occidental	2	89	460	672	15	1379	2017
7	Negros Oriental	2	45	269	400	8	806	1199
12	North Cotabato	2	64	356	478	24	1067	1433
8	Northern Samar	2	27	152	196	7	455	588
3	Nueva Ecija	4	64	294	631	17	883	1893
2	Nueva Vizcaya	1	17	82	127	3	246	382
17	Occidental Mindoro	1	23	103	148	6	308	443
17	Oriental Mindoro	4	35	111	240	7	332	719
13	PASAY CITY	3	0	23	101	0	68	303

17	Palawan	2	44	198	282	17	594	845
3	Pampanga	4	35	308	708	11	923	2125
1	Pangasinan	4	78	364	839	15	1092	2518
13	QUEZON CITY	4	0	187	926	0	562	2777
4	Quezon	2	80	237	503	18	712	1509
2	Quirino	1	8	36	50	2	108	151
4	Rizal	4	0	575	1239	0	1726	3717
17	Romblon	1	11	47	68	1	142	204
8	Samar (Western Samar)	2	33	175	230	7	525	689
12	Sarangani	2	28	161	212	9	484	635
7	Siquijor	3	2	8	13	1	24	40
5	Sorsogon	2	35	163	255	7	490	766
12	South Cotabato	2	26	241	328	7	724	985
8	Southern Leyte	3	19	39	83	2	118	249
12	Sultan Kudarat	2	37	207	276	13	621	827
15	Sulu	2	32	256	327	9	768	981
16	Surigao del Norte	1	18	106	171	5	317	513
16	Surigao del Sur	3	25	76	187	5	228	560
3	Tarlac	4	35	185	426	8	556	1279
15	Tawi-Tawi	1	22	109	140	4	327	420
3	Zambales	1	15	144	230	5	433	689
9	Zamboanga Sibugay	2	27	167	216	7	500	648
9	Zamboanga del Norte	2	40	248	330	9	743	989
9	Zamboanga del Sur	2	29	220	293	6	661	880

Table 20. Prioritization criterion among HCPN facilities

High priority	PCF	Level 1 (high gap means above median gap)	Level 2 (high gap means above median gap)
Level 1 hospital	with gap PCF	high gap in L1	high gap in L2
PCF	with gap PCF	low gap in L1	high gap in L2
Level 1 hospital	with gap PCF	high gap in L1	low gap in L2
PCF	with gap PCF	low gap in L1	low gap in L2
Level 1 hospital	no gap PCF	high gap in L1	high gap in L2
Level 2 hospital	no gap PCF	low gap in L1	high gap in L2
Level 1 hospital	no gap PCF	high gap in L1	low gap in L2
Level 1 hospital	no gap PCF	low gap in L1	low gap in L2

The cost to reduce environmental risk and improve resilience in health facilities

The proposed costs of building or expanding health facilities (Table 25 and 26) does not account measures to reduce environmental risk and increase resilience. How much will it cost to construct

a safe, sustainable resilient health facility?

The costs to build and expand new health facilities are related to protective measures needed to withstand identified risks due to natural or environmental hazards. Other measures and standards are related to increase in the general safety, sustainability, and ability to respond to disasters. Most of these measures are context-dependent thus only general costing can be calculated. It depends on the location of the building and the type of environmental risk in the area. Exact costing must be made based on a specific architectural design for a specific health facility in a specific location.

The Plan proposes a simple formula:

$$Cost = S \times E \times R$$

Where:

- Cost equate to the total cost for the construction works, whether retrofitting, an extension or a completely new health facility.
- S are costs related to the size of the construction works (in square meter or in bed capacity)
- E are cost factors for the environmental risk on that location
- R are cost factors for resilient measures

The total cost for the construction work, be it retrofitting, an extension, or a completely new health facility. Total cost includes expense for the complete building and the technical infrastructure (electricity, water, sanitation, ICT, waste). It does not include medical equipment and furniture. Extension is a new building connected or separate from the existing structure, while retrofitting is the installation of parts or equipment within the existing building.

S are costs related to the size of the construction works (in square meter or in bed capacity). Table 21 below shows the current estimations based on historical data of the construction costs per square meter and costs per bed for a new health facility.

Table 21. Estimated cost of health facilities (in 2020 prices)

	Type of health care facility	Proxy for costs in Php*
New	Barangay health station (BHS)	1,750,250 per facility
New	Rural health unit (RHU)	4,775,750 per facility
New or increase in bed capacity	Hospital Level 1	4,038,222 / bed
	Hospital Level 2	4,366,944 / bed
	Hospital Level 3	4,659,740 / bed

*Note: *Inflation and a higher ambition level for the future healthcare facilities, will both increase the costs of healthcare facilities; costs per bed for APEX hospital are based on a level 3 hospital with 250 bed capacity*

E are cost factors for the environmental risk of that specific location. The local conditions and context should be considered in computing for total costs of a specific health facility. Although several variables such as topography, climate conditions, soil conditions, transportation, labor and material costs ought to be included, only identified risks following a climate change and disaster risk evaluation and their corresponding adaptations are considered below.

Table 22. Factors for environmental risk measures

Identified risk	Adaptations	Factor (E)*
Earthquake risk	Appropriate foundation system; extra horizontal reinforcements; robust detailing of connections of structural elements	1.03
Flood risk	Locate all critical infrastructure above flood level; increase floor levels; waste water treatment unit with flood capacity	1.04
Typhoon risk	Roof cover adjustment; specific materials for walls and window; locate all critical infrastructure above flood level; increase floor levels; waste water treatment unit with flood capacity	1.05

*Note: *Factor is calculated is based on cost calculations for the above proposed adaptations on a reference plan*

R are cost factors for resilient measures. A set of measures, using the framework for safe and resilient health facilities is defined in order to estimate costs for resilience. Three scenarios are defined each with different levels of measures and outcomes namely, basic, medium, and high.

Table 23. Factors for resilient measures

Scenarios	Description	Resilient measures/ components	Benefits
**Scenario 1**<br>**Basic**<br><br>R=1.05*	Standard building requirements were followed	General<br>• Regular construction standards of the Philippine government (National Building Code, etc.)<br>• Off-grid powered communication, IT system	HCF is able to communicate and function after a disaster event (up to a minimum of 72 hours after))

**Scenario 2**<br>**Medium**<br><br>R=1.3*	Improved resilience will be achieved through higher standards for the main structure, critical systems and robust materials<br><br>Higher quality level of health care is achieved with improved disaster response, better medical protocols, improved management and higher qualified human resources	Basic plus the following:<br><br>*Structural components*<br>• Structural integrity<br>• Floor to floor height of four (4) meters<br>• Insulated roof<br>• use of robust and sustainable materials<br><br>*Critical components*<br>• Energy saving and generation<br>• Efficient heating, ventilation and air-conditioning (HVAC<br>• Water supply and distribution plus savings<br>• Hospital waste management and separation implemented<br>• Surge protection of medical equipment<br>• Protection of (medical) stock	Basic plus the following:<br>• Structure will be safe during all disaster events<br>• Reliable, stable water supply and electricity<br>• Functioning medical equipment<br>• Cost savings on energy, water and waste<br>• Reduction of environmental risk of medical waste<br>• Reduction of maintenance burden<br>• Reduction of greenhouse gas emissions
**Scenario 3**<br>**High**<br><br>R=1.5*	High level of resilience will be achieved through higher standards for the main structure, critical systems, functionality and robust materials<br><br>Higher quality level of health care and is achieved with improved functional design, better infection control measures, increased disaster response, better medical protocols, improved management and qualified human resources	All above plus the following:<br><br>*Site components*<br>• Topography, vegetation<br>• organized for mass influx<br><br>*Non-structural components*<br>• Use of robust, durable and sustainable materials<br>• Shading, insulated walls and reflective materials<br>• Optimization of natural ventilation<br>• Infection Prevention Plan<br><br>*Functional components*<br>• Sustainable and resilient design (space for patients)<br>• Prevent heat islands, install roof gardens<br>• Dedicated personnel for maintenance and disaster preparation<br>• Infection prevention and control plan implemented	All above plus the following:<br>• Well organized in case of emergency and able to receive mass influx of patients capable to managing surge capacity<br>• Less maintenance on walls, windows, doors, etc.<br>• Less heating from the sun<br>• Improved infection prevention<br>• More space and better functional design contribute to better health outcomes<br>• More pleasant environment for patients, healthcare workers and visitors

*Note: * Factor is calculated based on cost calculations for the above proposed resilient measures on a reference plan*

Raising the standard of new health facilities or upgrading the existing stock to a higher standard that is resilient to climate change and disasters will necessarily increase the cost of health facilities. However, the initial higher investment will be paid back after a few years and is more cost-effective in the long term. Strategic investments in sustainable and resilient health facility infrastructure are cost effective as these structures, especially hospitals, generally have long lifespans and will be most beneficial for governments. Table 24 shows the expected return of investments (ROI) of selected resilient measures.

Table 24. Return of investment (ROI) of resilient health infrastructure

Measure	Return of investment (ROI)	Benefits
Solar Rooftop system on-site electricity generation	8-9 years	Independent of public grid<br>Cheaper on the long run
High-efficient chillers in HVAC system	7-8 years	Lower energy consumption<br>Less dependent on energy
Efficient lighting	3-5 years	Lower energy consumption<br>Less dependent on energy
Water saving fixtures and management	1 year	Less waste of water<br>Less dependent on supply from local water grid
Waste separation and reduction	1 year	Better control over the hazardous waste<br>Less volume to manage<br>Money savings through contract
Solar water heating	10-15 years	Less energy required for heating up water<br>Less dependent on energy
On-site drinking water production	3-5 years	Reduction of plastic bottles waste<br>No need to purchase water<br>Less dependent on supplier

CHAPTER VI

Localization and Operationalization

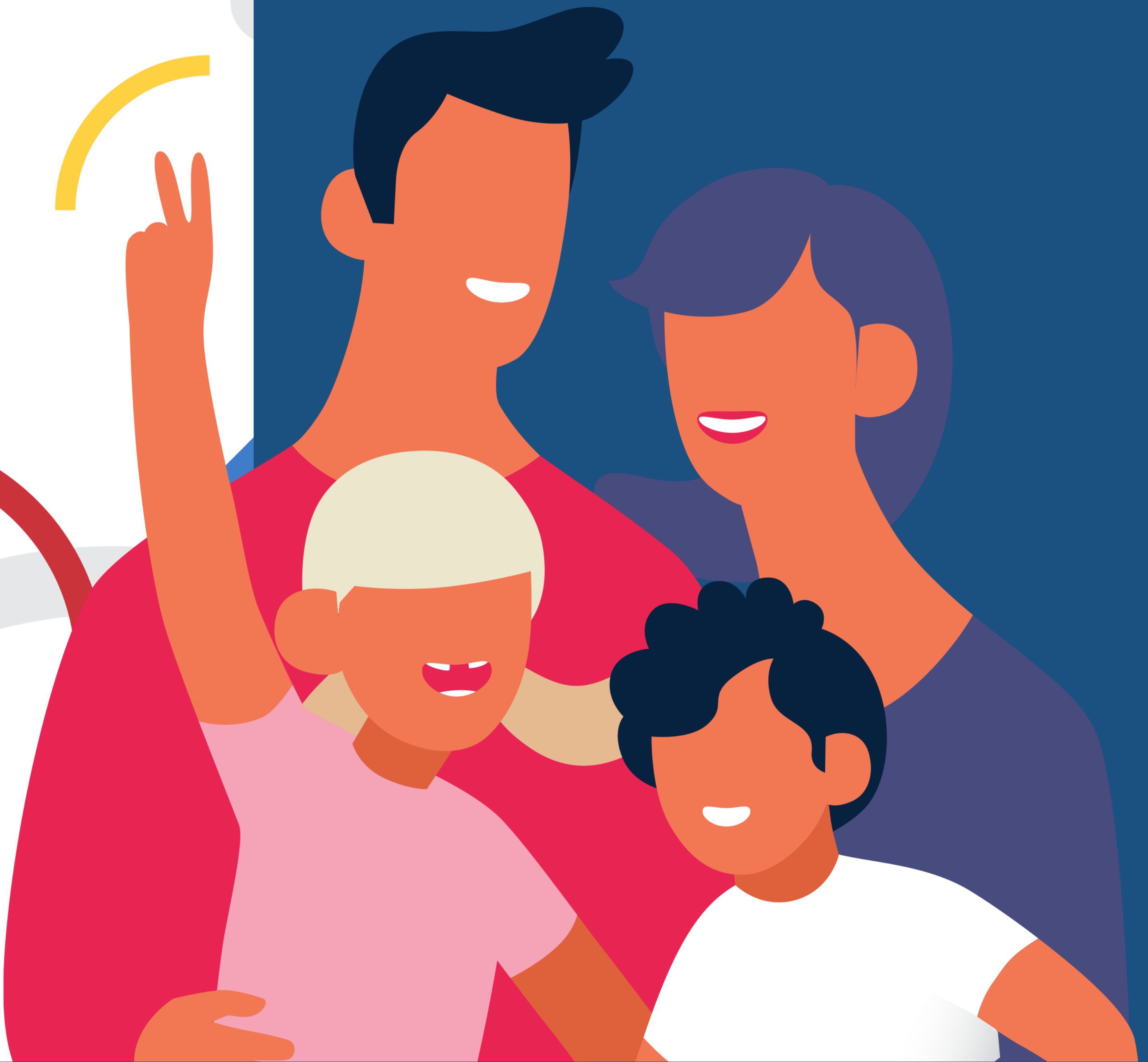

The previous chapter presents the current supply of health facilities and the expected need in the future. However, practical questions remain:

- How should these numbers inform the investment plan of province and HUCs/ICCs?
- What are the financing options and strategies to address the health infrastructure gaps of province and HUCs/ICCs?

This chapter proposes practical steps to inform local health investment plan. The health system capacity, and the socio-economic environment varies across province and HUC/ICC. The steps therefore must be adapted by considering the local context.

There are six (6) general actions that provinces could follow to localize the Plan:

Step 1: Commit to a goal	Envision a provincial health system with a network of different types of health facilities
Step 2: Examine the supply and need	Identify and validate the supply, the gap, and the needed investments of the province
Step 3: Identify the financing options	Assess fiscal space for health and explore different types of sources of financing
Step 4: Identify strategies	Explore innovative strategies to realize health infrastructure goals
Step 5: Contextualize the gap	Identify important factors in constructing or expanding health facilities
Step 6: Incorporate in the LIPH	Incorporate the gap analysis and strategies in the Local Investment Plan for Health

Figure 41. Steps to Localize the PHFDP

Step 1: Commit to a goal

Local governments should accelerate one of the core tenets of the UHC Act. That is, province and HUC/ICC should have a network of health facilities offering healthcare services in an integrated and coordinated manner. Each provincial and city-wide HPCN should have the following core facilities:

- Primary care network
  - Barangay Health Station (BHS) in each barangay
  - Primary Care Facilities accessible to everyone. That is, accessible within 30- minute distance.
- Level 1 hospital

- Level 2 hospital
- Ancillary facilities (that is diagnostics, pharmacy, and ambulance) to support the functions of primary care network facilities and hospitals
- The province or HUC/ICC must be attached to an Apex hospital

Building a provincial HPCN is a long-term vision, which requires long-term investment and planning. Provincial governments must have the commitment and support of local government leaders, civil society, private sector, and the constituency.

Local government leaders should be aware of the goals of HPCN and the required investment. For health facilities owned by municipal governments, local government leaders must commit and support the integration of their RHUs with other health facilities in the province.

Also, awareness and support from the private sector, civil society, and the general public are critical. In a system in which primary care and referral system are priorities, it requires a significant change on how Filipinos seek healthcare services.

The provincial government should assess the maturity level in implementing UHC. The Department of Health (DOH) has identified key reforms areas needed to facilitate UHC and integration of care, including referral transport, surveillance, health promotion through the Local Health System Maturity Levels (AO 2020-0037). The following are the activities of the provincial government in ensuring commitment and support:

**Activity 1: Ensure that goals are aligned with LGUs**	Ensure the LGUs support the vision of HPCN. Their commitment should be reflected in their local investment plans.
**Activity 2: Engage other sectors**	Improve awareness of the public, civil societies, the private sector about the vision of HPCN.
**Activity 3. Assess maturity level of the province**	Assess the maturity of the province or HUC/ICC to implement the UHC and integrated care, and commit to address the deficiencies. See DOH document on maturity framework.

Step 2. Examine the supply and need

If the goal and commitment are set, the provincial government should examine the supply and gap in health facilities in their province.

- **First step.** Validate the total (cumulative) gap of primary care facilities and levels 1 and 2 hospitals in the province. This information can be found in Appendix A.
- **Second step.** Distribute the need in the next twenty (20) years to align with the national goal, which is to close the cumulative gap in BHS and RHU of the province by 2025. The cumulative gap in level 1 and 2 hospital beds should be addressed in the next twenty years (20) by

spreading the need quinquennially (every five years).

To illustrate, if the cumulative gap of level 1 hospital beds in province A is 400, the target is to build 100 beds for every five years (see C in the table below).

	Gap in 2025	Gap in 2030	Gap in 2035	Gap in 2040
**Primary care**<br>BHS (A)<br>Primary care facilities (B)	 70<br>25	 0<br>2	 0<br>2	 0<br>2
Level 1 hospital bed (C)	100	100	100	100
Level 2 hospital bed (D)	25	25	25	25

- **Third step.** Identify the expected share of the gap by public and private sectors. To align with the national goal, the share of primary care network facilities (BHS and RHU) should be covered by public funds hence the 100% share. The share of public and private facilities by province or HUC/ICC can be found in Appendix A. The provinces may opt to use ground data, local expert opinions, or actual target share of public or private hospital beds depending on the private market penetration of the province.

	Gap in 2025	Gap in 2030	Gap in 2035	Gap in 2040
**Primary care**<br>BHS (A)<br>Primary care facilities (B)<br>Public (B1)	 100%<br>100%<br>100%	 No gap<br>100%<br>100%	 No gap<br>100%<br>100%	 No gap<br>100%<br>100%
Level 1 hospitals I<br>Public (C1)<br>Private (C2)	100%<br>X%<br>X%	100%<br>X%<br>X%	100%<br>X%<br>X%	100%<br>X%<br>X%
Level 2 hospitals (D)<br>Public (D1)<br>Private (D2)	100%<br>X%<br>X%	100%<br>X%<br>X%	100%<br>X%<br>X%	100%<br>X%<br>X%

- **Fourth step.** Determine the quinquennial (every five years) targets by ownership. The table below shows the number of BHS, primary care facility (that is RHU), and hospital beds assuming the share of private hospital beds to total hospital beds was set by planners at 50%.

	Gap in 2025	Gap in 2030	Gap in 2035	Gap in 2040
**Primary care**<br>BHS (A)<br>Primary care facilities (B)<br>Public (B1)	70<br>25<br>25	No gap<br>2<br>2	No gap<br>2<br>2	No gap<br>100%<br>100%
Level 1 hospitals I<br>Public (C1)<br>Private (C2)	100<br>50<br>50	100<br>50<br>50	100<br>50<br>50	100<br>50<br>50
Level 2 hospitals (D)<br>Public (D1)<br>Private (D2)	25<br>12<br>13	25<br>12<br>13	25<br>12<br>13	25<br>12<br>13

- **Fifth step.** Calculate the total cost of constructing health facilities in the next twenty (20) years by multiplying the actual gap and the unit cost of constructing BHS, PCF, level 1 and level hospitals.
    - For the private sector investments (C1 and C2), the province should call for private investors to build hospitals. From the example above, the private sector is expected to build level 1 hospital with 200 bed capacity and level 2 hospital with 50 bed capacity in the next 20 years. Box 2 contains innovative strategies to encourage the private sector to supplement health infrastructure.
    - For the public sector investments (A, B, C1, D1), primary care network facilities (A, B) will be the priority in the next five (5) years to align with the national goal, which is to close the gap in primary care network facilities by 2025. However, from 2026 onwards, public investments (C1 and D1) for L1 and L2 hospitals will be the priority.

Box 2: what are strategies to encourage the private sector to expand health facilities within the national vision?

Tax exemptions and incentives can be offered to private. For instance, private hospitals can be exempted from wide-range of local and national taxes (e.g., income taxes, property taxes, and import duty taxes) especially in areas where is there large scarcity of health infrastructure as long as they are willing to be part of the HPCN and accept pro-equity conditionalities set by the national and local governments.

Design an attractive financing scheme. Currently, PhilHealth case rates are the same for public and private hospitals. However, public sector hospitals receive direct subsidies from government through salaries and capital outlay in addition to PhilHealth reimbursements. As a result, the cost of healthcare services paid through the social health insurance scheme is much higher for public hospitals than private hospitals. To remove this imbalance, explore the possibility of higher reimbursements for the private sector. PhilHealth might also explore weighted case rates. Higher case rates in provinces where there is scarcity of health facilities.

Provide subsidies to the private sector. The private sector will build facilities in government lands as long as they are willing to be part of the HPCN, and willing to accept pro-equity conditionalities. The national government should provide both financial grants and non-financial assistance for LGU decision-makers to adopt such approach.

Step 3. Identify financing options

Once the required public sector investment is determined (A, B, C1, D1), the provincial government should explore sources of financing primary care network facilities (BHS and RHU), levels 1 and 2 hospitals. The Plan proposes the following steps in identifying options.

- **First step.** Assess the historical budget for health and health infrastructure allocated by the provincial and local governments. This is to determine how much the province and LGUs typically spend on health infrastructures vis-à-vis expected health infrastructure expenditures.
- **Second step.** Assess fiscal space for health by identifying the level of public expenditures, revenues, and deficit. It is also necessary to examine the historical priority spending of the provincial government. This exercise will allow the provincial government to examine the feasibility of mobilizing resources for health infrastructure through budget increase or through reprioritization (that is, re-allocation from non-efficient expenditures).
- **Third step.** Identify different sources of financing considering the fiscal capacity of the province in the medium to long-term. The table below shows the indicative sources of financing that that province or HUC/ICC should explore.

Table 25. Sources of financing

Sources of financing	Strategies
LGU revenue (tax and non-tax	• The province and municipalities should broaden tax and non-tax base revenues to finance health infrastructures.<br>• Re-allocate less efficient expenditures to health infrastructure expenditures. This could be explored especially among provinces with relatively low health spending to total public expenditures (less than 5%).
IRA and national share	• Allocate higher IRA to finance health and health infrastructures. In the medium to long-term, higher IRA share is expected because of the Mandanas ruling.
PhilHealth	• Provinces and HUCs need to maximize PhilHealth income to finance health expenditures. Explore the possibility of using PhilHealth income to finance MOOE and capital outlay. In theory, provinces are not precluded to use PhilHealth income to finance capital outlay.
Loans	• The local government should explore loans from financial institutions.
Grants from the national government (that is Health Facility Enhancement Program)	• In the next chapter, the Plan provides a national allocation framework that identifies provinces to be prioritized by the national government for grants and other subsidies.

Step 4. Explore innovative strategies

Traditionally, the construction and expansion of facilities (A, B, C1, D1) initiated by local governments using public resources are constructed through the usual route. That is, provinces and local governments individually build and manage the health facility.

The province should explore innovative practices and models in building and managing health facilities.

Explore bundled procurement

- Provinces should engage the private sector in the construction of multiple government health facilities through bundled procurement. After the construction, the private sector will turn over to the provincial and local governments for it to manage and operate.

Harness the private sector to provide certain functions of the HPCN.

- Subcontract the building and management of clinical functions of the HPCN (that is ancillary services of the network (that is laboratories/diagnostics, pharmacies)
- Subcontract the building of and management of non-clinical functions of the HPCN (that is IT system connecting primary care network facilities, ambulance networks)
- Public and private concessions (that is primary care network to be built and managed entirely or partly by the private sector)

Step 5. Contextualize the gap

The previous chapter aids provinces and HUCs to examine the gaps for each type of health facility. The next step is for the province or HUC to contextualize these gaps in their own settings.

Primary Care Facilities (PCF)

- **First step.** Establish the gap in PCF in the province or HUC. Note the gap is based on the population without access to PCF within 30 minutes. Annex A contains the supply and gaps in PCF by province.
- **Second step (1):** If there is gap, identify where to build the PCF. Annex B contains the maps of each province with different types of health facilities. This should aid provinces and HUCs to identify where to strategically locate the facility.
- **Second step (2):** If there is no gap - a case mostly in highly urbanize cities because time travel is not a challenge, the local government should consider the following:
    - Increase the number of primary care physicians (PCPs) working in one PCFs. While facilities may be accessible, services may be constrained because of workforce availability. In these instances, addition of workforce is critical. See Annex A.
    - Expand the physical structure of the PCF to accommodate patients.
- **Third step.** If there is a gap, the province and HUC should explore two options.
    - Build PCF in the area without PCF
    - Use of telemedicine in the nearest BHS in lieu of building a physical PCF.
- **Fourth step.** If the provinces and HUC/ ICCs will build a PCF, decision-makers should consider the following:
    - Availability of land and building
    - Accessibility of the area by public/ private transportation
    - Topography of the land and existing soil conditions
    - Access to public utilities such as water, sewerage system, electricity, telephone line, and internet
    - Level of potential hazard, which will inform the kind of physical and architectural design of the PCF. The assessment will examine the level of different types of hazards, such geological, hydrometeorological, man-made. See: https://hazardhunter.georisk.gov.ph/
    - Prevailing socio-political-cultural conditions/ situation, including presence of indigenous communities and other vulnerable populations

Level 1 and 2 Hospital Beds

- **First step.** Establish and validate the gaps in levels 1 and 2 hospitals beds.
- **Second step.** Determine the hospital safety and resilience index to assess the required

upgrade of the existing hospital infrastructure. Determine level of potential hazard of the area. See: https://hazardhunter.georisk.gov.ph/

- **Third step.** Determine options to addressing the gap in hospital bed:
  - Expand the number of beds in L1 or L2 beds of existing hospitals.
  - Upgrade infirmaries to Level 1 hospitals.
  - Build new Level 1 or 2 hospitals in low access areas.
- **Fourth step.** If decision makers decide to build a new hospital, the province and HUC/ICC should think about the following:
  - Strategic location of the hospital. The access maps in the appendix should provide a general picture of the distribution of health facilities. See Annex A.
  - Level of potential hazard, which will inform the kind of physical and architectural design of the new hospital. The assessment will examine the level of different types of hazards, such geological, hydrometeorological, man-made. See: https://hazardhunter.georisk.gov.ph/
  - What laws support this and/ or what laws need to be passed to realize this new hospital?
  - What policies and strategies are needed to ensure adequate financing and human resource availability to sustain hospital operations? What incentives need to be institutionalized?
  - What policies and strategies are needed to employ to support future operations such as access to supplies, medicines and other supply chain considerations.
  - What are the determinants of health seeking behavior in the locality? (i.e. underutilization of facilities may be due to perceived poor quality of care or associated financial cost of services and not due to poor physical access)

CHAPTER VII

Special Facilities

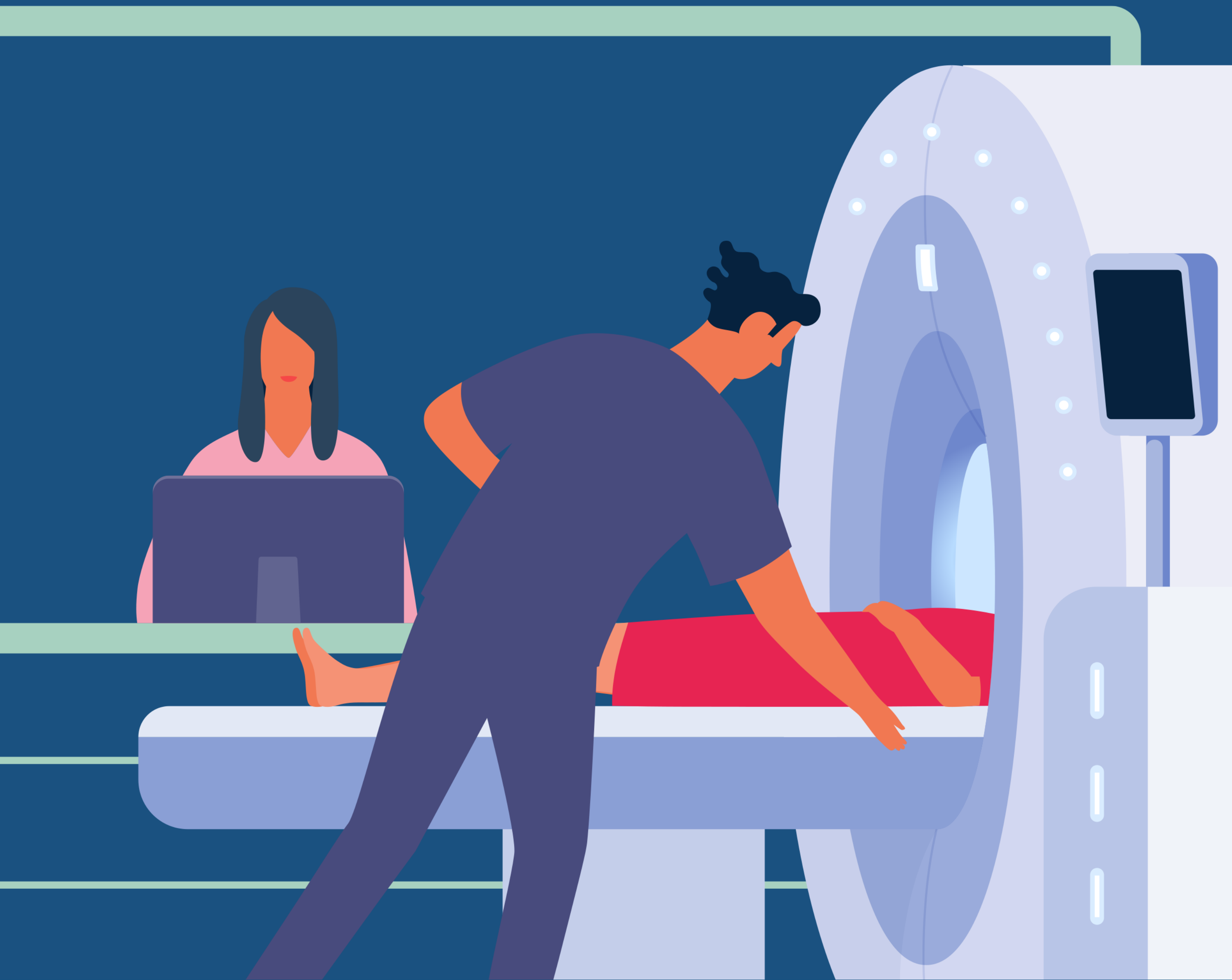

While core facilities (health stations, primary care facilities, and hospitals) in HCPNs are meant to provide care for the majority of patients, particular services require specialized care from facilities with capability to cater to specific patient populations for particular procedures and overall care. This section focuses on special facilities meant to complement province- and city-wide HCPNs.

These facilities include:

- Specialty Centers
- Specialized Laboratories
- Blood Service Facilities,
- Drug Abuse Treatment and Rehabilitation Facilities
- Military Health Facilities
- Hospitals of State Universities and Colleges

A. Specialty Centers

i. Rationale

Under the Universal Health Care (UHC) Act of 2019, health care provider networks (HCPNs) composed of health facilities providing primary to tertiary level care are linked to an apex or end-referral hospital and other facilities providing specialized services needed by its catchment population. To ensure accessibility of health facilities across the continuum of care, select DOH hospitals will be upgraded to operate with Specialty Centers for one or more identified medical and/or surgical specialties and shall serve as apex or end referral hospitals of HCPNs. This section focuses on the resources needed for selected DOH Hospitals to become Specialty Centers.

Specialty care services were selected by the DOH based on the country's: top burden of disease, significant laws and mandates, and commitment to special needs. Other specialty care services not included in the list will be integrated in the service capability of a general hospital (i.e. reproductive health, pediatrics).

Top Burden of Disease. The following specialty care services addressing the leading causes top mortality and morbidity were considered:

a. Cardiovascular Care
b. Cancer Care
c. Lung Care
d. Renal Care
e. Mental Health
f. Brain and Spine
g. Neonatology
h. Trauma and Burn
i. Orthopedic Care and Physical Rehabilitation
j. Infectious Disease

Significant Laws & Mandates. Table 26 shows the priority specialty services with existing laws and mandates, especially those signed into law in recent years.

Table 26. Laws and Mandates relevant to Specialty Care Services

- Infectious Diseases - COVID-19, Pneumonia, Tuberculosis, HIV/AIDS, Malaria (Republic Act 11469, Sustainable Development Goals)
- Cancer Control Law (Republic Act 11215)
- Geriatrics Wards (Republic Act 9994)
- Mental Health Law (Republic Act 11036)
- Orthopedics & Physical Rehabilitation and Medicine (Republic Act 2679, Republic Act 6786)
- Cardiovascular Care (Presidential Decree No. 673)
- Lung Care (Presidential Decree No. 1823)
- Renal Care and Kidney Transplant (Presidential Decree No. 1832)

Special Needs. Other specialty care services were selected to address commitment to special needs and neglected conditions of the country.

a. Eye Care - Blindness is a top contributor to disability in the country.
b. Toxicology - There is a need to establish expertise in addressing toxicologic emergency response, especially for poisoning.
c. Dermatology Care - Many systemic disease conditions have skin manifestations (i.e., HIV/AIDS, Lupus) and several neglected conditions cause stigma (that is, psoriasis) and may inequitably affect the poor (i.e., leprosy, skin infections)

To arrive into the crafting of a 20-year development for upgrading of Specialty Centers for selected hospitals with defined milestones and success indicators by 2022, 2030 and 2040, a structured process was undertaken by the fifteen (15) technical working groups (TWGs).

The DOH utilized a resource-stratified framework (RSF) in the development of health facility standards. As described in Chapter III of this plan, the RSF intended to define the essential resource requirements necessary at each level of care - from the most basic health station all the way to national specialty centers. Specialty care services are at the end of the continuum, offering advanced and highly-complex care, often requiring highly specialized health workforce, equipment and infrastructure. The RSF was adapted to our local context, practice and policies. Outputs underwent stakeholder consultations with various partners, followed by service capability mapping of existing facilities. TWGs utilized these inputs to craft the development plan for each of the Specialty Centers.

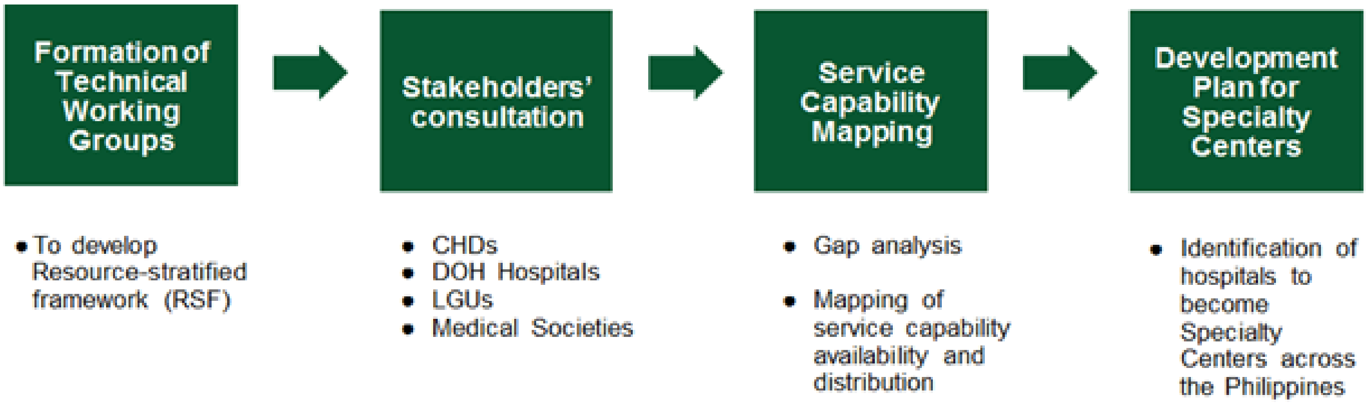

Figure 42. Process for Creating the Development Plans for Specialty Centers

ii. Roles and Responsibilities

A Specialty Center is a unit or department within a licensed Level 3 hospital that offers highly specialized care addressing particular conditions and/or providing specific procedures and management of cases requiring specialized training and/or equipment (AO 2020-0019). Table 27 shows the sixteen (16) Specialty Centers to be established in selected DOH Hospitals.

Table 27. Different types of Specialty Centers

I. Cardiovascular Care	IX. Physical Rehabilitation Medicine
II. Lung Care	X. Infectious Disease and Tropical Medicine
III. Renal Care and Kidney Transplant	XI. Toxicology
IV. Cancer Care	XII. Mental Health
V. Brain and Spine Care	XIII. Geriatric Care
VI. Trauma Care	XIV. Neonatal Care
VII. Burn Care	XV. Dermatology Care
VIII. Orthopedic Care	XVI. Eye Care

Specialty care may be provided in three different types of specialty centers with varying levels of service capability. Each of the sixteen (16) specialties have identified National Specialty Centers, Advanced Comprehensive Specialty Centers and Basic Comprehensive Specialty Centers to primarily deliver specialty care for specific catchment areas (Figure 43). Details of each type as well as their roles and responsibilities are listed below.

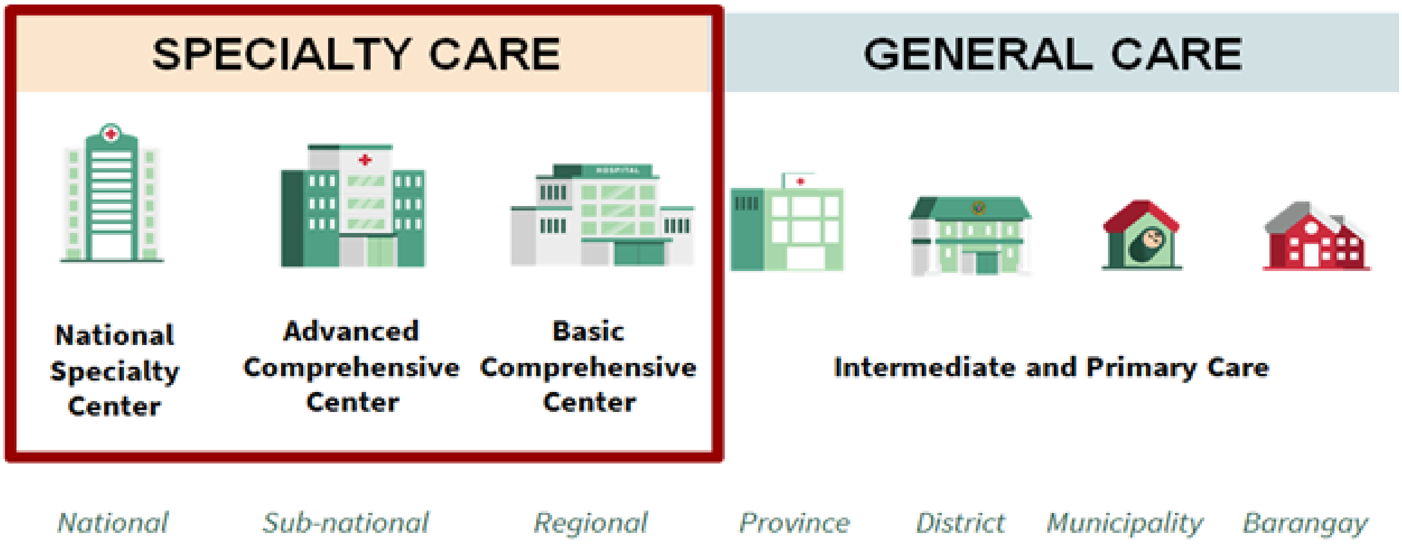

Figure 43. Health Facilities and their Catchment Population
Specialty Centers in red box

National Specialty Center (NSC) – a Level 3 specialty or general hospital with the highest level of expertise in clinical services, teaching and training, and research in a given specialty and is the country's apex or end-referral facility for a given specialty. NSCs have the following roles and responsibilities:

- Assume the responsibility of being the core data/ information hub for their respective specializations and diseases they cover, in coordination with concerned DOH offices;
- Lead in the development of policies, protocols, and standards for the particular specialty and shall have the highest level of clinical services, training, and research;
- Provide scientific leadership in research by conducting specialized clinical, public health, and operations research with a multidisciplinary or multi-center clinical approach;
- Lead selected DOH hospitals in establishing Specialty Centers by providing specialty training and technical assistance in collaboration with the DOH and relevant professional organizations;
- Oversee the Advanced Comprehensive and Basic Comprehensive Specialty Centers to ensure delivery of quality services and strengthen the network of care across the country for the specific specialty.

Advanced Comprehensive Center (ACC) – a Level 3 specialty or general hospital which serves as apex or end-referral facility at the subnational or regional level with advanced level of comprehensive clinical services, serves as a facility for specialty and subspecialty training, and with capacity for multi-specialty, multi-center clinical, public health, and operations research.

Basic Comprehensive Center (BCC) – a Level 3 specialty or general hospital which generally serves as apex or end-referral facility at the regional level, capable of managing complex cases, serves as a facility for specialty training, and capable of conducting clinical, operational, and public health

research.

iii. Development Plan & Cost

The designated DOH hospitals which shall serve as National Specialty Centers, Advanced Comprehensive Specialty Centers, and Basic Comprehensive Specialty Centers are presented in Annex D. Details of the target year of establishment and estimate costs for each specialty center are also in Annex D. Table 28 below summarizes the total estimated cost for each of the specialty centers until 2025. Actual cost may vary depending on the existing facilities of each DOH hospital and other local considerations.

Table 28. Costing for Specialty Centers (in millions Php)

Specialty Centers	2022	2023	2024	2025
Cancer Care Centers	10,056	6,122	1,850	4,594
Cardiovascular Care Centers	2,490	750	750	81
Renal Care and Transplant Centers	5,710	914	1,300	-
Lung Care Centers	5,390	3,060	5,500	1,660
Brain and Spine Care Centers	1,132	1,132	1,942	2,693
Orthopedic Centers	7,210	-	-	4,612
Physical Rehabilitation Medicine Centers	743	276	496	-
Mental Health Care Centers	-	274	1,054	822
Neonatal Care Centers	-	389	814	396
Eye Care Centers	882			
Dermatology Centers	238			
Burn Care Centers	-	399	226	453
Trauma Care Centers	-	1,198	1,974	1,751
Toxicology Centers	-	337	527	338
Infectious Disease and Tropical Medicine Centers	1,566	486	648	324
Geriatric Centers	1,241			
**Total**	**39,108**	**10,332**	**15,608**	**17,437**

B. Specialized Laboratories

i. Rationale

Using the RSF for Infectious Diseases and Tropical Medicine, a framework for the upgrading of the Laboratory Network was crafted to ensure access to quality highly-specialized laboratory services.

ii. Types of Specialized Laboratory Facilities

Specialized laboratories in the network include the National Reference Laboratory (NRL), Sub-

national Reference Laboratories (SRL), which is an extension of the NRL, Regional and Provincial Laboratories, and Specimen Collection Laboratory. The service capabilities of NRL, SNLs, and Regional and Provincial Laboratories are listed in Table 29 while the designated DOH facilities, up to the regional level, are indicated in Table 30.

Table 29. Summary of Service Capability for Laboratories

Facility level	National Reference Laboratory	Sub-national Refer-ence Laboratory	Regional Laboratory	Provincial Labor-atory	Specimen Col-lection Labora-tory
Catchment	National	North Luzon<br>NCR<br>South Luzon<br>Visayas<br>Mindanao	Regional & Provincial	Provincial	District Hospitals &<br>Primary Health Care Facilities
Service Capability	Biosafety Level 3 (BSL 3)<br><br>Sets standards, guidelines, and protocols	Biosafety Level 2 (BSL 2)	Biosafety Level 2 (BSL 2)	Tertiary Laboratory<br>*At least 1 lab with RT-PCR per province*	Specimen collection

Table 30. Facilities in the Specialized Laboratory Network

National Reference Laboratory: Research Institute of Tropical Medicine		
Luzon	Visayas	Mindanao
**Sub-national Reference Laboratories**		
NCR - San Lazaro Hospital*<br>NCR - Lung Center of the Philippines*<br>CAR - Baguio General Hospital and Medical Center*	VII - Vicente Sotto Memorial Medical Center*	XI - Southern Philippines Medical Center
**Regional Laboratories**		
I - Mariano Marcos Memorial Hospital and Medical Center<br>II - Cagayan Valley Medical Center<br>III - Jose B. Lingad Memorial General Hospital<br>IV-A - Batangas Medical Center<br>IV-B - Ospital ng Palawan<br>V - Bicol Regional Diagnostic and Reference Laboratory	VI - Corazon Locsin Montelibano Memorial Regional Hospital<br>VI - Western Visayas Medical Center<br>VIII - Eastern Visayas Regional Medical Center	IX - Zamboanga City Medical Center<br>X - Northern Mindanao Medical Center<br>XII - Cotabato Regional and Medical Center<br>XIII - Caraga Regional Hospital<br>BARMM - Amai Pakpak Medical Center

iii. Development Plan & Cost

The estimated cost for the upgrading the NRL, SRL and Regional Specialized Laboratories are summarized in Table 31.

Table 31. Cost Estimate for Upgrading the Specialized Laboratory Network (in millions)

Facility Category	Cost Per Unit		Estimated Total Cost
	Infrastructure	Equipment	
National Reference Laboratory (Research Institute for Tropical Medicine into BSL 3)	7.5	18	25.5
Subnational Reference Laboratory (5 DOH Tertiary Labs into BSL 2)	6	18	120
Regional Laboratories (40 Tertiary Labs into BSL 2)	4.8	18	912
**Total Cost for Upgrading the Laboratory Network**	**1,060**		

C. Blood Service Facilities

i. Rationale

Republic Act (RA) 7719, also known as "National Blood Services Act of 1994", mandates the safe and efficient blood banking and transfusion services in the country. In Section 5, it is stated that the DOH, in cooperation with the Philippine Red Cross (PRC), Philippine Blood Coordinating Council (PBCC), other government agencies and non-governmental organizations shall plan and implement a National Voluntary Blood Services Program (NVBSP) to meet in an evolutionary manner, the needs for blood transfusion in all regions in the country.

The DOH-NVBSP is created as the overall policy making and program planning body which include among others, the conceptualization, planning, and coordination of core activities such as promotion of voluntary blood donation, public education and advocacy, upgrade of blood services and facilities, and effective and equitable collection and distribution of blood and other resources.

Blood Services Networks (BSN) are meant to ensure availability and accessibility of voluntarily donated blood and blood products within a particular geographic catchment and are integrated within and across Health Care Provider Networks (HCPN).

The Philippine National Blood Services, comprise the various Blood Service Facilities (BSF) owned by the DOH, various Local Government Units, Private and the PRC.

Pursuant to RA 7719 section 2, item G, the DOH is mandated to establish and organize a National Blood Transfusion Network in order to rationalize and improve the provision of adequate and safe

supply of blood. Section 4 item D states that Blood Centers shall be strategically established in every province and city nationwide within the framework of this National Blood Transfusion Service Network.

Blood Services Networks (BSN) are composed of all identified BSFs with designated Lead and Satellite Blood Service Facilities, hospitals and non-hospital-based health facilities performing transfusion (government and private), National Reference Laboratory, LGUs, community-based volunteer donors and partner agencies. BSNs are established to provide the blood needs of specific geographic areas in the Philippines through efficient and effective blood transport and distribution system of voluntary donated blood. It is mandated to ensure blood availability to all patients, maximizing utilization of available blood and avoiding wastage. One of its objectives also is to advocate for adherence to patient safety standards in all its procedures.

Further, BSFs are intended to provide safe, adequate and accessible blood supply to patients and transfusing facilities which include hospitals or other non-hospital facilities such as dialysis clinics and other stand-alone facilities. Policies/ issuances relevant to standards and operations of blood service facilities are listed below.

- AO 2005-0002: Rules and Regulations for the Establish of the Philippine National Blood Services Amending Pertinent Provisions of Admin Order No. 9, s. 1995 (Rules and Regulations Implementing R.A. 7719 Otherwise known as the National Blood Services Act of 1994)
- AO 2008-0008 and 2008-008A: Rules and Regulations Governing the Regulation of Blood Service Facilities
- AO 2010-0001: Policies and Guidelines for the Philippine National Blood Services and the Blood Services Networks
- Manual of Standards for Blood Service Facilities
- Blood Donor Selection and Counselling Manual
- Philippine Clinical Practice Guidelines for the Rational Use of Blood and Blood Products and Strategies for Implementation

ii. Classification/Types/Roles and Responsibilities

Blood Centers (BC) have the highest level of service capability among all Blood Service Facilities (BSF), and are classified into three (3) namely, National Blood Center, Subnational Blood Center and Regional Blood Center. These Blood Centers designated as the Lead BSF shall have responsibility to conduct close supervision of each Satellite BSF within its Blood Service Network. These facilities shall be identified in the Certificate of Inclusion issued by Center for Health Development (CHD) and recommended by the Lead Blood Service Facility for compliance to their licensing requirements. Blood centers are non-hospital-based and can be managed and owned by the DOH, LGU or the PRC.

All Level 2 or 3 national and local government hospitals as well as Level 1 Basic Emergency Obstetrical and Neonatal Care (BEmONC) providers are mandated to have either a blood station or a blood bank within their hospital premises to ensure timely access to needed blood for their patients.

All Blood Banks are hospital-based. Blood Stations may be hospital-based or standalone facilities while Blood Collecting Units are non-hospital based. All BSF are likewise mandated to utilize the National Blood Bank Network System (NBBNetS) to facilitate traceability of donated blood, validation of test results and access to other pertinent information. The resource-stratified framework (RSF) for Blood Service Facilities are available in Annex E.

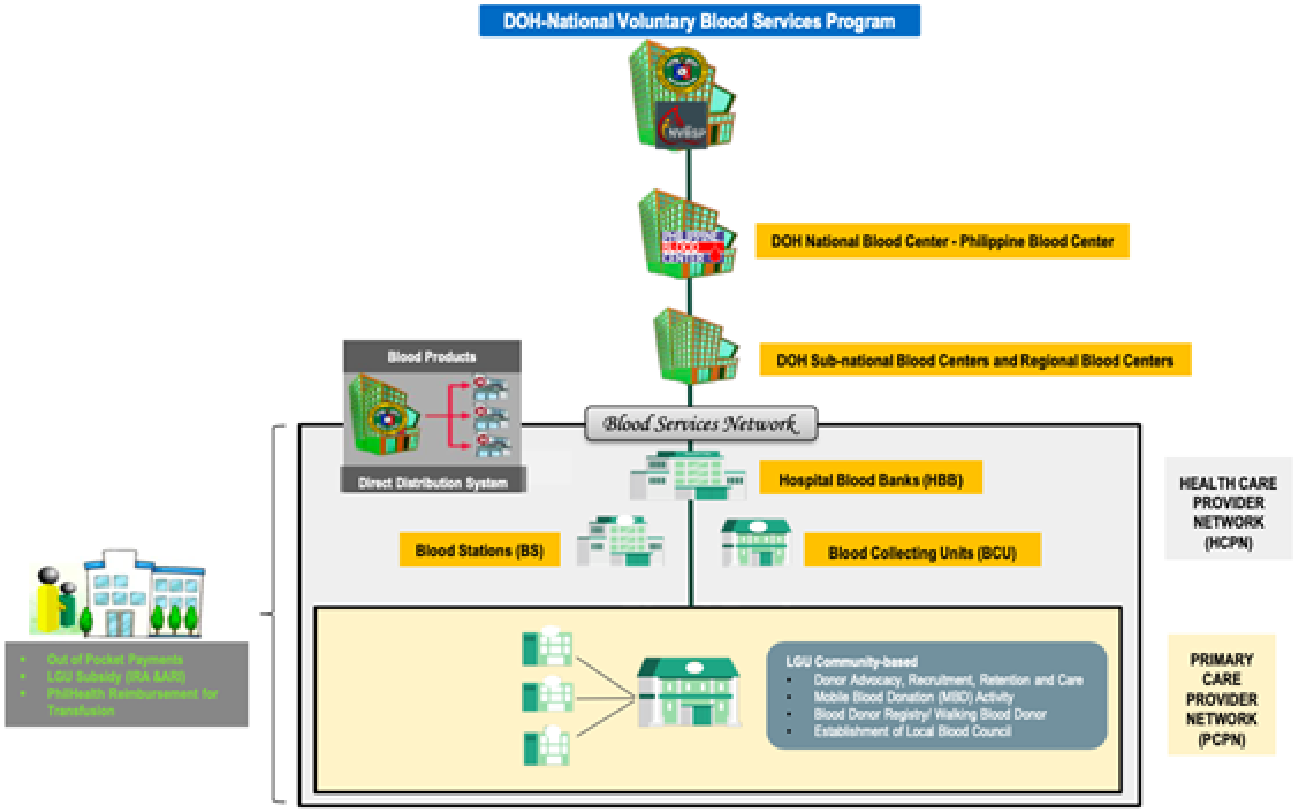

Figure 44. Blood Service Network

Table 32.Current Classification of Blood Service Facilities

	Blood Center	Blood Bank	Blood Station	Blood Collecting Unit
Type of Institution	Non-Hospital-based	Hospital-based	Hospital-based or Non-Hospital-based	Non-Hospital-based
Ownership	DOH, LGU or Philippine Red Cross (PRC)	Government or Private Hospital	PRC, LGU, Government or Private Hospital	PRC, LGU, Government or Private

General description of Service Capability	a. Advocacy and promotion for voluntary blood donation and healthy lifestyle<br>b. Recruitment, retention and care of voluntary blood donors<br>c. Collection of blood (mobile or facility-based) from qualified voluntary blood donors<br>d. Conduct health education and counseling<br>e. Testing of units of blood for TTIs<br>f. Processing and provision of blood components<br>g. Storage, issuance, transport and distribution of units of whole blood and/ or blood products to hospitals and other health facilities	a. Advocacy and promotion of voluntary blood donation and healthy lifestyle<br>b. Storage and issuance of whole blood and blood components obtained from a Blood Center<br>c. The following services shall also be provided:<br>i. Compatibility testing of red cell units<br>ii. Direct Coombs Test<br>iii. Red cell antibody screening<br>iv. Investigation of transfusion reactions<br>v. Assist the Hospital Blood Transfusion Committee (HBTC) in the conduct of post transfusion surveillance (hemovigilance)	a. Advocacy and promotion of voluntary blood donation and healthy lifestyle<br>b. Provision of whole blood and packed red cells<br>c. Storage, issuance, transport and distribution of whole blood and packed red cells<br>d. Compatibility testing of red cell units, if hospital based	a. Advocacy and promotion of voluntary blood donation and healthy lifestyle<br>b. Recruitment, retention and care of voluntary blood donors<br>c. Screening and selection of voluntary blood donors<br>d. Conduct of health education and counseling services<br>e. Collection of blood (community based) from qualified voluntary blood donors<br>f. Transport of blood to Blood Center for testing and processing

Table 33. Blood Centers in the Philippines in the context of Universal Health Care (UHC) Act

	National Blood Center	Subnational Blood Center	Regional Blood Center	Regional/ Provincial/ City and Inter-Provincial/ Inter-City Blood Centers	
Service capability	*Details available in the attached Resource Stratified Framework for BSF (Annex E)*				
Ownership	DOH	DOH	DOH	PRC	LGU
Number (Operational Blood Centers)	1	2	1	29	4

iii. Development Plan & Cost

Target development plans for blood service facilities will focus on meeting access standards and having additional blood services. Currently, there are two established Subnational Blood Centers, meant to mirror most of the service capability of the National Blood Center. This is intended to minimize unnecessary travel of patients as well as for appropriate processing of blood and blood products, and to allow better access to blood services across the country.

Baseline for Regional Blood Centers is one facility in Region V. Expansion of blood service facilities will include availability of at least one DOH Blood Center for all other regions. Detailed costing of Blood Service Facilities is available in Annex F.

Table 34. Development Plan for DOH Blood Centers with Estimated Total Cost

DOH Blood Centers	Baseline 2020	2021	2022	2025
National Blood Center				
Philippine Blood Center (NCR)	95,230,563	130,000,000	50,000,000	For PPP
Sub-national Blood Centers				
Region VII	70,230,563	115,000,000		
Region XI	95,230,563	90,000,000		
Regional Blood Centers				
Region I	40,480,563		128,750,000	
CAR				266,650,000
Region II		169,230,563		
Region III		128,750,000		
Region IV-A				266,650,000
Region IV-B				266,650,000
Region V		169,230,563		
Region VI				266,650,000
Region VIII				266,650,000
Region IX			128,750,000	
Region X			128,750,000	
Region XII			128,750,000	
Region XIII				266,650,00
BARMM				266,650,00
**TOTAL (Php)**	**301,172,252**	**802,211,126**	**565,000,000**	**1,866,550,000**
**Grand TOTAL (Php)**	**3,534,933,378**			

D. Drug Abuse Treatment and Rehabilitation Facilities

i. Rationale

Drug rehabilitation is defined as the process of medical or psychotherapeutic treatment for dependence on psychoactive substances such as alcohol, prescription drugs, and other dangerous drugs. This process of drug rehabilitation may be facilitated through residential programs or as outpatient services in specialized health facilities.

The Comprehensive Dangerous Drugs Act of 2002 (RA 9165), signed in 2002, provides the mandate for Department of Health to oversee, monitor, supervise all drug rehabilitation, intervention, after-care and follow-up programs including the establishment, operations and maintenance of drug

treatment and rehabilitation facilities, both government and private, in coordination with other agencies such as the Dangerous Drugs Board, Department of Social Welfare and Development and the Department of the Interior and Local Government.

As aligned to principles articulated in the UHC Act 2019, Drug Abuse Treatment and Rehabilitation Facilities shall be organized into networks, providing services within and across HCPNs. As specialized facilities, these cater to specific populations and complement services that may not be available in facilities providing generalized care.

Policies/ issuances relevant to Drug Abuse Treatment and Rehabilitation Facilities:

- RA 9165: Comprehensive Dangerous Drugs Act of 2002
- RA 11223: Universal Health Care Act 2019
- DBM-DOH Joint Circular No. 1, s. 2014: Standards on Organizational Structure and Staffing Pattern of Treatment and Rehabilitation Centers
- AO2019-005: Guidelines for the Establishment of Pilot Recovery Clinic for Persons who use drugs (PWUD)

ii. Roles and Responsibilities

There are four (4) types of health facilities included in the network of Drug Abuse Treatment and Rehabilitation Facilities namely, Subnational Drug Abuse Treatment and Rehabilitation Center (DATRC), Regional DATRC, Provincial/ City Outpatient DATR Facilities (Outpatient DATRC Facilities including Recovery Clinics and Homes), and Community Based Drug Rehabilitation Program (CBDRP). The service capability of each facility is reflected in the resource stratified framework detailed in Annex G. In addition to these, Level 2 hospitals are expected to be able to provide immediate care for patients requiring emergency services including detoxification of patients diagnosed with substance use and substance induced-psychosis among others.

Access to drug abuse treatment and rehabilitation facilities are to be distributed following the description below. An overview of the differentiation of facilities is also provided in Table 35.

- Subnational DATRCs shall be located in five (5) strategic locations namely NCR, North Luzon, South Luzon, Visayas, and Mindanao.
- Regional DATRCs shall be made available in all regions to provide inpatient treatment and rehabilitation services.
- Outpatient DATR Facilities shall be available in all provinces and highly-urbanized cities which may be a stand-alone facility or attached to a Level 2 hospital.
- Community-based drug rehabilitation centers are owned and managed by LGUs and may be operated by an Anti-Drug Abuse Council in barangay health stations or primary care facilities (i.e. Rural Health Unit/ Health Center).

Table 35. Summary of Service Capabilities of Drug Abuse Treatment and Rehabilitation Facilities

	Subnational DATRC	Regional DATRC	Outpatient DATR Facility/ Recovery Clinic	Community-Based Drug Rehabilitation Center +	
Catchment	Cluster of regions	Region	Province/ City	Municipality	Barangay
Ideal (Minimum) Distribution/ Access	One per subnational cluster	One per region	One per Province/ City	One per Munici-pality	One per Barangay
General description of service capability	Residential treatment and rehabilitation program for those diagnosed with Severe Dependence Substance Use Disorder (SUD) with additional capability for those with co-occurring psychiatric or medical conditions.<br>Special Populations such as Women, Adolescent, and those with infectious conditions (PTB)	Residential treatment and rehabilitation program for those diagnosed with Severe Dependence SUD	Intensive Outpatient Treatment and rehabilitation program for those with Moderate SUD	Outpatient treatment and rehabilitation program for those with Mild SUD<br>Aftercare and reintegration programs<br>General Interventions	Outpatient treatment and rehabilitation program for those with Mild SUD<br>Aftercare and reintegration programs<br>General Interventions
Type of patient (persons who use drugs) appropriate for the level of care	1. Severe SUD<br>2. Severe SUD with a co-occurring medical or psychiatric condition<br>3. Special Populations PWUDs (People Living with HIV, Women, Adolescent, and those with infectious conditions (PTB)**	Severe uncomplicated SUD	Moderate SUD	Mild SUD	Mild SUD

**Details available in Annex: Resource-stratified framework for Drug Abuse Treatment and Rehabilitation Facilities*
+Community-Based Drug Rehabilitation Programs are services integrated into the service capabilities of RHUs and City Health Centers.

iii. Development Plan & Cost

Drug abuse treatment and rehabilitation centers shall be established in different parts of the country for improved distribution of services. Subnational DATRC shall be distributed in five (5) strategic locations while Regional DATRCs shall be established in each region where there is no Subnational DATRC. Tables 36-37 show the results matrix and development plan of these facilities and their target completion dates. Total cost estimates are in Table 38.

Table 36. Results Matrix for select Drug Abuse Treatment and Rehabilitation Facilities

Indicator	Baseline (2020)	2022	2025	2030	2035	2040
Number of upgraded Subnational DATRC	1	2	3			
Number of new Regional DATRCs			2			
Number of upgraded Region DATRCs	11			11		
Provincial/ City Outpatient DATR Facility	8	15	20	25	25	20

Note: Numbers in each column reflect additional facilities to be added by the identified milestone year

Table 37. Development Plan for Drug Abuse Treatment and Rehabilitation Facilities

	Facility Name	Target Year of Completion
**Subnational DATRCs**		
NCR	DOH Bicutan TRC	2022
North Luzon	DOH TRC Dagupan	2025
South Luzon	DOH CamSur TRC, DOH Malinao TRC	2025
Visayas	DOH TRC Argao, DOH TRC Cebu City	2025
Mindanao	DOH TRC Malagos, Davao	2022
**Regional DATRCs**		
NCR	DOH Las Pinas TRC	2030
Region I (on-going)	DOH TRC Cordillera Autonomous Region	2022
Region II	DOH TRC Isabela	2030
Region III	DOH TRC Bataan	2030
Region IV-A	DOH Tagaytay City TRC	2030
Region IV-B	*New Facility	2025
Region VI	DOH TRC Iloilo	2030
Region VIII	DOH TRC Dulag	2030
Region IX	DOH TRC Zamboanga City	2030
Region X	DOH TRC Cagayan De Oro City	2030
Region XII	SOCCSKSARGEN DATRC	2030
Region XIII	DATRC CARAGA	2030
BARMM (New)	*New Facility	2025
**Outpatient Drug Abuse Treatment and Rehabilitation Facilities and Recovery Clinics**		
NCR - Valenzuela	Valenzuela Medical Center	2025
NCR - Pasay City	Pasay City	(Currently Operational)
NCR - Paranaque	Ospital ng Parañaque	2025
CAR - Benguet	Outpatient Drug Abuse Treatment and Rehabilita-tion Center- Baguio General Medical Center	(Currently Operational)
CAR - Ifugao	Lagawe, Ifugao	(Currently Operational)
Region XI - Davao City	Region XI Outpatient and Aftercare Center for Drug Depend-ents	(Currently Operational)

Region I - Dagupan	Region 1 Medical Center	2025
Region II - Isabela	Southern Isabela General Hospital	2025
Region II - Quirino	Quirino Provincial Medical Center	2025
Region II - Nueva Vizcaya	Region II Trauma and Medical Center	2025
Region III - Tarlac	Tarlac Drug Abuse Treatment and Rehabilitation and Drug Testing Laboratory	(Currently Operational)
Region III - Bataan	Bataan General Hospital	2025
Region IV A - Batangas	Batangas Medical Center	2025
Region IV A - Quezon	Quezon Medical Center	2025
Region IV B - Oriental Min-doro	Calapan	(Currently Operational)
Region V - Sorsogon	Dr. Fernando B. Duran Sr. Memorial Hospital	2025
Region V - Masbate	Masbate Provincial Hospital	2025
Region VI - Iloilo	Western Visayas Medical Center	2025
Region VI - Aklan	Dr. Rafael S. Tumbokon Memorial Hospital	2025
Region VI - Antique	Angel Salazar Memorial General Hospital	2025
VI - Guimaras	Dr. Catalino Gallego Nava Provincial Hospital	2025
Region VII - Cebu	Vicente Sotto Memorial Medical Center	2025
Region VII - Cebu City	Mandaue	(Currently Operational)
Region VIII - Leyte	Eastern Visayas Regional Medical Center	2025
Region VIII - Samar	Samar Provincial Hospital	2025
Region VIII - Biliran	Biliran Provincial Hospital	2025
Region IX - Zamboanga Del Norte	Zamboanga del Norte Medical Center	2025
Region X - Misamis Oriental	Northern Mindanao Medical Center	2025
Region X - Cagayan de Oro City	Northern Mindanao Medical Center	2025
Region XI - Davao De Oro (Compostela Valley)	Nabunturan	2022
Region XI - Davao del Norte	Davao Regional Medical Center	2025
Region XII - South Cotabato	South Cotabato Provincial Hospital	2025
Region XIII (CARAGA) - Surigao del Norte	CARAGA Regional Hospital	2025
BARMM - Maguindanao	Cotabato Sanitarium	2025

Table 38. Cost Estimate of Infrastructure and Equipment for Drug Abuse Treatment and Rehabilitation Facilities (in millions Php)

Facility	Cost per Unit			No.	Estimate Total Cost
	Infra structure	Equip ment	Total		
**Subnational DATRCs***<br>North Luzon-DOH TRC Dagupan<br>NCR-DOH Bicutan TRC<br>South Luzon-DOH CamSur TRC and DOH Malinao TRC<br>Visayas-DOH TRC Argao and DOHTRC Cebu City<br>Mindanao-DOH TRC Malagos, Davao	370	185	555	5	**2,775**
**Regional DATRCs**<br>(For Establishment of New Regional DATRC)<br>Region IV-B, BARMM	488.14	236.54	724.72	2	**1,450**
**Regional DATRCs**<br>(For upgrading of existing 100 Bed capacity to become 300 Bed capacity)<br>· Region I: DOH TRC Luis Hora<br>· Region II: DOH TRC Isabela<br>· Region III: DOH TRC Bataan<br>· NCR: DOH Las Pinas TRC<br>· Region IV-A: DOH Tagaytay City TRC<br>· Region VI: DOH TRC Iloilo<br>· Region VIII: DOH TRC Dulag<br>· Region IX: DOH TRC Zamboanga City<br>· Region X: DOH TRC Cagayan De Oro City<br>· Region XII: SOCCSKSARGEN DATRC<br>· Region XIII: DATRC CARAGA	200	100	300	11	**3,300**
**Outpatient DATR Facility/ Recovery Clinic*****<br>For Establishment of New Outpatient DATR Facility	5.56	2.78	8.35	105	**876.75**
**TOTAL**					**8,401.75**

**Cost estimates for upgrading existing bed capacity to 500 Bed Capacity DATRC and upgrading of equipment.*
***Cost estimates for upgrading from existing bed capacity to 300 Bed Capacity DATRC and upgrading of equipment.*
****Based on 2017 Cost Estimates for Outpatient Recovery Clinics. LGU's may opt to construct a dedicated facility for their CBDRP or utilize their existing health facilities.*

E. Military Health Facilities

i. Rationale

Grounded in the Armed Forces of the Philippines' (AFP) core values of honor, service and patriotism, the mission of the Armed Forces of the Philippines Health Service (AFPHS) is to conserve the fighting strength of the AFP and to effectively manage medical service resources through the application of diagnostic, therapeutic, rehabilitative, restorative and preventive medicine in maintaining the health and wellbeing of military personnel and their dependents.

Facilities of the AFPHS are unique primarily because of the population they serve. These health facilities cater to military personnel and their dependents and are not for the general civilian population. While services provided in the facilities may mirror general and specialized health services detailed in other sections above, the health workforce of the AFPHS require additional training on competencies as aligned with the unique needs of the military.

The Office of the Surgeon General heads the AFPHS and leads all medical and health matters relevant to the AFP, including the Office of the Chief Surgeon of each of the Major Services (Army, Air Force, Navy) and the Commanding Officers of Medical Treatment Facilities, Unified Commands, AFP-Wide Service Support Separate Units and Major Services including Medical Service Units namely the Medical Corps (MC), Veterans Corps (VC) and Medical Administrative Corps (MAC).

AFPHS health facilities intend to contribute the following population-based services:

- Enhance health screening of AFP Personnel and dependents
- Support immunization programs and screening especially in GIDAs or during medical missions and operations during calamities and disasters
- Strengthen health promotion through nationwide conduct of health education, screening and health surveillance through AFP Public Health Service Center Support govt efforts in response to epidemics or outbreaks

ii. Roles and Responsibilities

The continuum of care provided by military treatment facilities span the range of health promotion/ prevention, ambulatory care, emergency and inpatient care, rehabilitative care all the way to long-term care and end-of life care, spanning different services offered by facilities of varying levels of service capability. Tables 42 and 43 below provide an overview of the different types of facilities that are currently available.

Table 39. Summary of different types of Military Health Service Facilities

	National Medical Centers	General Hospitals	Station Hospitals	Infirmaries	Medical Dispensaries
Role in the network	Apex facility providing definitive, rehabilitative and specialized care	Definitive and rehabilitative care for each of the major service	Definitive care	Resuscitation, Emergency medical care	Resuscitation, Emergency medical care
Role	Role 4: Definitive Care	Role 3: Theater Hospitalization	Role 3: Theater Hospitalization	Role 1 and 2: First responder and Forward Resuscitative	Role 1 and 2: First responder and Forward Resuscitative
Rear/ Forward Medical Service Support	Rear	Rear	Rear	Forward	Forward

Table 40. Military Treatment Facilities and Veterans Hospital

Region	City/ Province	Hospital	ABC
**Level 3 Hospitals**			
NCR	Quezon City	Victoriano Luna Medical Center	1200
NCR	Quezon City	Veterans Memorial Medical Center	766
Level 2 Hospitals			
NCR	Quezon City	PNP General Hospital	176
NCR	Pasay City	Air Force General Hospital	100
**Level 1 Hospitals**			
NCR	City of Taguig/ Pateros	Army General Hospital	200
NCR	City of Taguig/ Pateros	Manila Naval Hospital	85
Region II	Isabela	Camp Melchor F. Dela Cruz Station Hospital	30
Region III	Nueva Ecija	Fort Magsaysay Army Station Hospital	50
Region III	Tarlac	Camp Aquino Station Hospital	50
Region III	Pampanga	Basa Airbase Hospital	25
Region III	Pampanga	Airforce City Hospital	25
Region IV-A	Batangas	Fernando Air Base Hospital	70
Region IV-A	Cavite	Cavite Naval Hospital	100
Region IV-B	City of Puerto Princesa (Capital)	Camp General Artemio Ricarte Station Hospital	20
Region VII	City of Cebu (Capital)	Camp Lapu Lapu Station Hospital, Centcom, AFP	50
Region VIII	Samar (Western Samar)	Camp Lukban Station Hospital	25
Region IX	City of Zamboanga	Edwin Andrew's Airbase Hospital	29
Region X	City of Cagayan De Oro (Capital)	Camp Evangelista Station Hospital, 4ID, PA	100
BARMM	Maguindanao	Camp Siongco Station Hospital	50
**Infirmaries**			
NCR	Quezon City	Camp Gen. Emilio Aguinaldo Station Hospital	25
CAR	Baguio City	Fort del Pilar Station Hospital	50
CAR	Benguet	Camp Bado Dangwa Hospital	10
Region III	Bataan	Arsenal "Kalusugan" Hospital	10
Region V	Camarines Sur	Camp Elias Angeles Station Hospital	20
Region VI	Capiz	Camp Peralta Station Hospital	25
Region VII	Cebu	Brigadier General Benito N Ebuen Air Base Hospital	25
Region VII	Cebu	PNP Station Hospital	10
BARMM	Maguindanao	Camp Brig. Gen. Salipada K Pendatun Hospital	10

Note: ABC - Authorized Bed Capacity

iii. Development Plan & Cost

As the apex hospital for Military Health facilities, priority for the upgrading of V. Luna Medical Center is critical. The table below reflects the cost of upgrading of the emergency room, operating room and acute critical units.

Table 41. Estimated Cost of Upgrading of V. Luna Medical Center for 2021 (in millions PhP)

Facility Category	Estimated Total Cost
ER and OR (Medical Equipment, Furnitures and Fixtures, ICT Equipment)	429
Acute Critical Unit	3.5
**Total Cost**	**432.5**

F. Hospitals of State Universities and Colleges (SUC-Hospitals)

i. Rationale

Hospitals of State Universities and Colleges are unique health facilities that play an important role in HCPNs. Strongly linked with the academe, these hospitals significantly contribute to health workforce teaching and training as well as various types and levels of research. Currently, the country has four (4) SUC-Hospitals in NCR, Regions IV-A, VI, and XII that differ in service capability and bed capacity.

Table 42. SUC-Hospitals in the country

Region	City/Province	Hospital	Level	ABC
NCR	Manila	UP-Philippine General Hospital	Level 3	1334
IV-A - CALABARZON	Laguna	University Health Service	Level 1	30
VI - Western Visayas	Iloilo	West Visayas State University Medical Center	Level 3	300
XII - SOCCSKSARGEN	North Cotabato	University of Southern Mindanao (USM) Hospital	Level 1	70

Note: ABC - Authorized Bed Capacity

ii. Classification/Types/Roles and Responsibilities

As SUC-Hospitals, their roles, as aligned with principles of Universal Health Care, are the following:

- Serve as a health facility of the HCPN
  - Depending on their service capability, these health facilities can either be general hospitals or Apex/ end-referral hospitals. Coordination and collaboration with other health facilities within and outside their HCPN is critical.
  - These hospitals may develop Specialty Centers as determined by the need of their catchment population and network. The UP-Philippine General Hospital has existing Specialty Centers for Trauma and Burn, Cancer Care, Eye Care, Dermatology, and Toxicology.
- Support the Return Service Agreement Policy. In accordance with the UHC Act, graduates of

allied and health-related government-funded scholarships will be deployed and trained in DOH-specified priority health facilities and fields of practice under the Return Service Agreement (RSA) policy of the Department of Health.

- Contribute to Teaching, Training and Research. As educational institutions, SUC-Hospitals provide teaching, training and research to its HCPN and catchment area. Other HCPNs may also establish partnerships with SUC-Hospitals to expand the scope of these services. Research agenda may be aligned to the National Unified Health Research Agenda and include clinical, laboratory, public health, operations and other relevant initiatives and topics, as defined.

CHAPTER VIII

Geolocating and Mapping Health Facilities

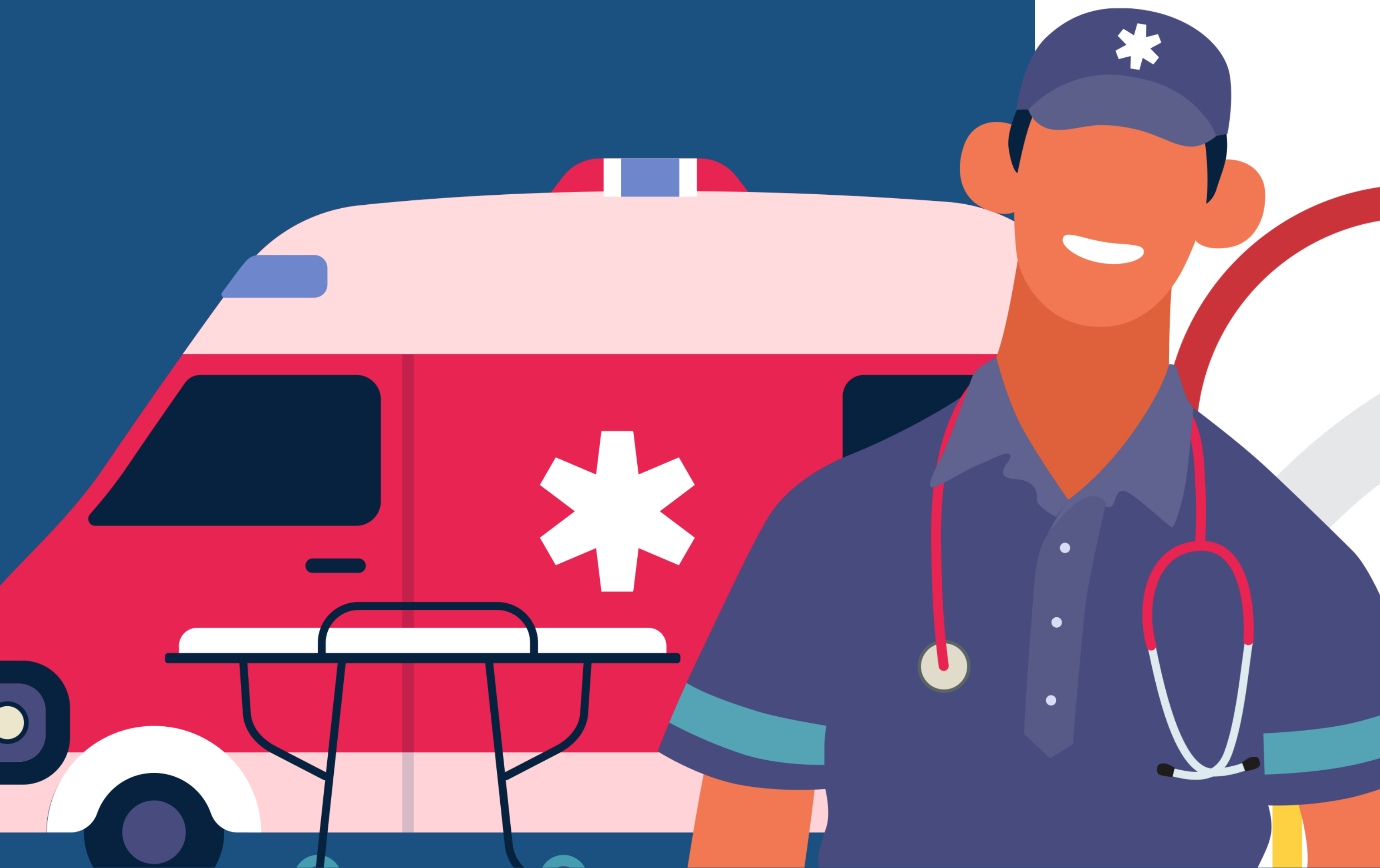

Reliable data is a prerequisite to good health infrastructure planning. This starts by accounting all healthcare facilities and determining their location on a map. Thus, the DOH is making an ongoing effort to collect and validate coordinates of all health facilities on a national scale in order to have a master list and map of the health infrastructure nationwide.

This section shows the efforts starting October 2019 to use data science to geolocate hospitals, infirmaries, primary care facilities (i.e. rural health units, health centers), and barangay health stations. From this effort, 82.77% of 24,644 of these facilities have certain coordinates (i.e. coordinates marked as having high precision from the NHFR, or coordinates with rooftop or approximate precision from the Google Geotagging API) while 4,711 (17.23%) have uncertain coordinates (i.e. coordinates failing to meet the above criteria).

Table 43. Nationwide Coordinate Certainties for Selected Facility Types

Facility type	Certainty	
	Certain	Uncertain
Barangay Health Station	18,561 (82.08%)	4,052 (17.92%)
Rural Health Unit/ Health Center	2,361 (91.12%)	230 (9.88%)
Hospital	1,184 (80.16%)	293 (19.84%)
Infirmary	538 (79.82%)	136 (20.18%)
**Total**	**22,644 (82.77%)**	**4,711 (17.23%)**

DOH Centers for Health Development and Local Government Units are invited to collaborate with the Health Facility Development Bureau (HFDB) and the Knowledge Management and Information Technology Service (KMITS) to update the coordinates of the health facilities within their respective areas to ensure their completeness and accuracy. This is critical for all ongoing and future efforts for data analytics and health facility planning. This will also ensure the credibility of supporting tools that will be made available in support of future planning activities.

Accessibility analysis was only conducted on provinces with 100% coordinate certainty for hospitals licensed in 2018. Among such provinces, accessibility analysis was done to calculate how many percent of the population of the province have access to a hospital within 1 hour in consideration of traffic and travel distance. This was the defined target travel time for accessibility of the population to a hospital. The process was repeated for 33 provinces with 100% coordinate certainty for RHUs.

Provisional maps are produced for this publication with the main objective of showing preliminary results of the Central Office's geolocating efforts.

After completing core facility coordinates, DOH's long term vision includes geolocating other facilities such as birthing homes, diagnostic facilities, medical outpatient clinics, specialized facilities, transition care facilities, drug abuse treatment and rehabilitation facilities, blood service facilities and other health-related establishments. Getting this data would require data sharing and collaboration between the private sector and governing agencies at all levels.

A. Baselining geospatial coordinates for each health facility

The initial coordinates of the health facilities were taken from two sources: (1) the National Health Facility Registry (NHFR) for hospitals, infirmaries and RHUs, and (2) the Service Capability Mapping (SCM) dataset for private outpatient clinics. The NHFR (downloaded on October 29, 2019) was considered as the master table since it is the most comprehensive list of health facilities at the time.

Sanity checking was performed to clean up outliers or invalid coordinates. Facilities with coordinates that were tagged as medium or low certainty by the NHFR, or no coordinates from NHFR, were then further geocoded using Geographic Information System (GIS).

Geocoding is the process of determining the GPS coordinates of places using available information such as facility name and location information. A three-way geocoding and verification were done between NHFR and open source tools (Google Places, Google Street View, and OpenStreetMap's library of points-of-interest).

Table 44. Nationwide coordinate certainties for all facility types

Facility Type	Coordinates Certainty		
	Certain	Uncertain	None
Ambulatory Surgical Clinic	2 (100.00%)		
Animal Bite Treatment Center	2 (33.33%)	4 (66.67%)	
Barangay Health Station	18,561 (82.08%)	4,052 (17.92%)	
Birthing Home	1,204 (63.20%)	689 (36.17%)	12 (0.63%)
City Health Office	7 (70.00%)	3 (30.00%	
DepEd Clinic	3 (33.33%)	6 (66.67%)	
Dialysis Clinic		8 (100.00%)	
Drug Abuse Treatment and Rehabilitation Centers	18 (30.00%)	42 (70.00%)	
Drug Testing Laboratory	13 (44.83%)	16 (55.17%)	
General Clinic Laboratory	67 (54.92%)	55 (45.08%)	
Hospital	1,184 (80.16%)	293 (19.84%)	
Infirmary	538 (79.82%)	136 (20.18%)	
Laboratory for Drinking Water Analysis	6 (100.00%)		
Municipal Health Office	9 (90.00%)	1 (10.00%)	
Occupational Dental Laboratory	2 (25.00%)	6 (75.00%)	
Private School Dental Laboratory	2 (100.00%)		
Provincial Health Office		1 (100.00%)	
Psychiatric Care Facility	3 (75.00%)	1 (25.00%)	
Rural Health Unit	2,361 (91.12%)	230 (8.88%)	
Social hygiene Clinic	20 (57.14%)	15 (42.86%)	
Special Clinical Laboratory		1 (100.00%)	
**Grand Total**	**24,002 (81.16%)**	**5,559 (18.80%)**	**12 (0.04%)**

B. Mapping health facilities

An overview map of hospitals, primary care facilities (i.e. RHU/ HC) and health stations were produced for each of the eighty-one (81) provinces and thirty-four (34) highly urbanized cities (HUCs) in the Philippines. These are available in Annex B.

The overview map is a combination of health facility location points overlaid on top of a choropleth. The choropleth represents the absence of Barangay Health Stations within the barangays of the province. Barangays where there was no BHS found were colored pink. The overlaid points represented hospitals, infirmaries, rural health units, and private outpatient clinics. Although BHS are considered a core health facility, it was only critical to determine if a barangay has at least one (1) BHS even without the exact coordinates of the BHS as per the Local Government Code of 1991.

For illustration purposes, Figure 45 shows the overview map of the province of Maguindanao with points as facilities. The facility type is differentiated by shape: cross for hospitals, diamond for infirmaries, hexagons for RHUs/ HCs. Government-owned facilities are green while private ones are red. Certainty is represented by the fill of the marker; those that are hollow do not have certain coordinates. The administrative boundaries shown are barangays, with those filled red as having no barangay health station, and the white polygons as having at least one BHS.

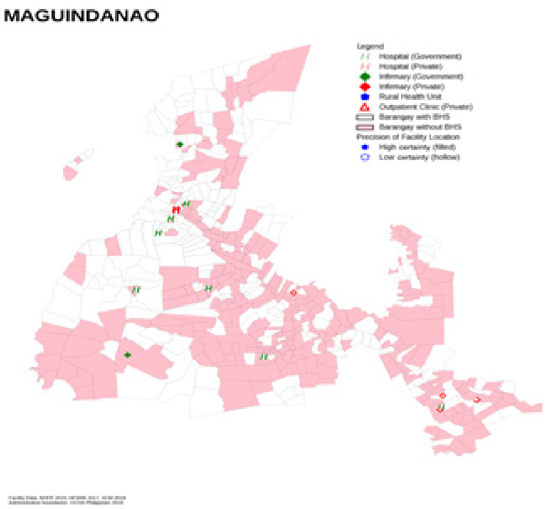

Figure 45. Health Facility Overview Map of Maguindanao

Using population data from Philippine Statistics Authority in 2015 and computed projections for 2019, two indicators were computed using health station presence and Rural Health Unit/ Health Centers counts against population: (a) how many people are living in areas without health stations; and (b) RHU-population.

Table 45. Coordinate certainties for main health facilities in Maguindanao

	Certain	Uncertain	No Coords	Total
Hospital	7 (63.64%)	4 (36.36%)	0 (0.0%)	11
Infirmary	2 (33.33%)	4 (66.67%)	0 (0.0%)	6
Rural Health Unit	35 (94.59%)	2 (5.41%)	0 (0.0%)	37

Sources of Coordinates: Google Maps, OpenStreetMap

Table 46. Barangay and population with a BHS in Maguindanao

	Count	Population (2015)	Population (2019)
With BHS	376 (48.5%)	1,221.163	1,324.726
Without BHS	399 (51.5%)	783,816	864,102

Table 47. Number and facility to population ratio for RHU in Maguindanao

	Count	RHU - Population Ratio
RHU	37	1 : 46,095 people

C. Accessibility Analysis for Hospitals and Rural Health Units

Among the 81 provinces, there are 30 provinces with 100% coordinate certainty on licensed hospitals, and 33 with 100% certainty of coordinates of RHUs. The Health Facilities and Services Regulatory Bureau (HFSRB) 2018 data was used to filter the hospitals to only the licensed ones. Hospital accessibility analysis was done to calculate how many percent of the population of the province have access to a hospital within 1 hour and to RHUs/ HCs within 30 minutes in consideration of traffic and travel distance through a motorized vehicle. These travel time standards have been set as targets for the realization of UHC. (Note: Time standards are specified in Table 5).

This approach has the following limitations. First, it only considers driving through a private vehicle and does not account for commuting by different transportation modes (walking, cycling, public transport, etc.). Second, it computes for the travel from the facility to the home and not from the home to the hospital. There is an assumption that the travel time and distance do not differ significantly in the opposite direction. Also, driving speed is based on the speed limit, and isochrones are generated with a preference for roads with the highest speed limits. Third, it doesn't account for travel over water (i.e. oceans, seas, rivers) and may affect results for provinces having separate islands. Fourth, the Philippines is listed under one of the countries for which Mapbox has limited predictability for travel times, having only partial coverage of traffic conditions. In addition, variability of traffic conditions in highly urbanized areas may be very drastic especially during rush hours. This may affect the accuracy of the resulting isochrones. Fifth, the analysis was done with set provincial boundaries. Thus, residents near borders may be close to facilities in adjacent provinces, but accessibility to these was only assessed within their province.

It is also important to note that the isochrones are a measure of physical access alone and are

not representative of other paradigms of access to health care, such as capacity, affordability, and quality.

This accessibility analysis involved the creation of isochrones, zones around each hospital and RHU that are reachable within a predetermined travel time. The threshold was set at 1 hour for hospitals and 30 minutes for RHUs, both through a motorized vehicle.

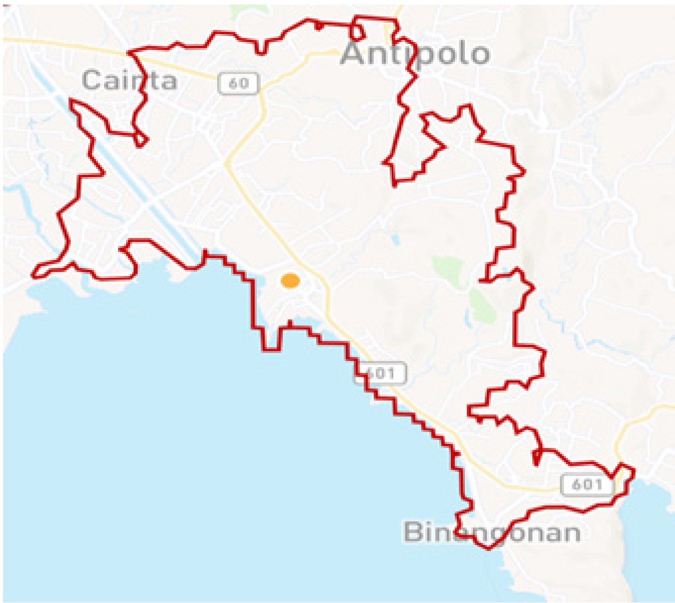

Figure 46. Sample of 60-minute isochrone around a health facility in Rizal

Isochrones were generated for each hospital and RHU. The isochrones were merged to classify the land area into two categories: high and low access. Infirmaries were not analyzed for physical access but were visualized together with the hospitals so that they can be considered when analyzing the map to determine strategically located infirmaries for possible upgrade to hospitals.

As an example, Figure 47 shows the access map of Kalinga. The administrative boundaries shown are municipalities and cities. The green shaded area is determined to be the coverage area of 1-hour travel from the hospital while the area in white is considered as having no access within 1 hour.

One municipality, Balbalan, was found to have no access to a public or private hospital within one hour. In contrast, 91% of the population in capital Tabuk has access to hospital care within an hour.

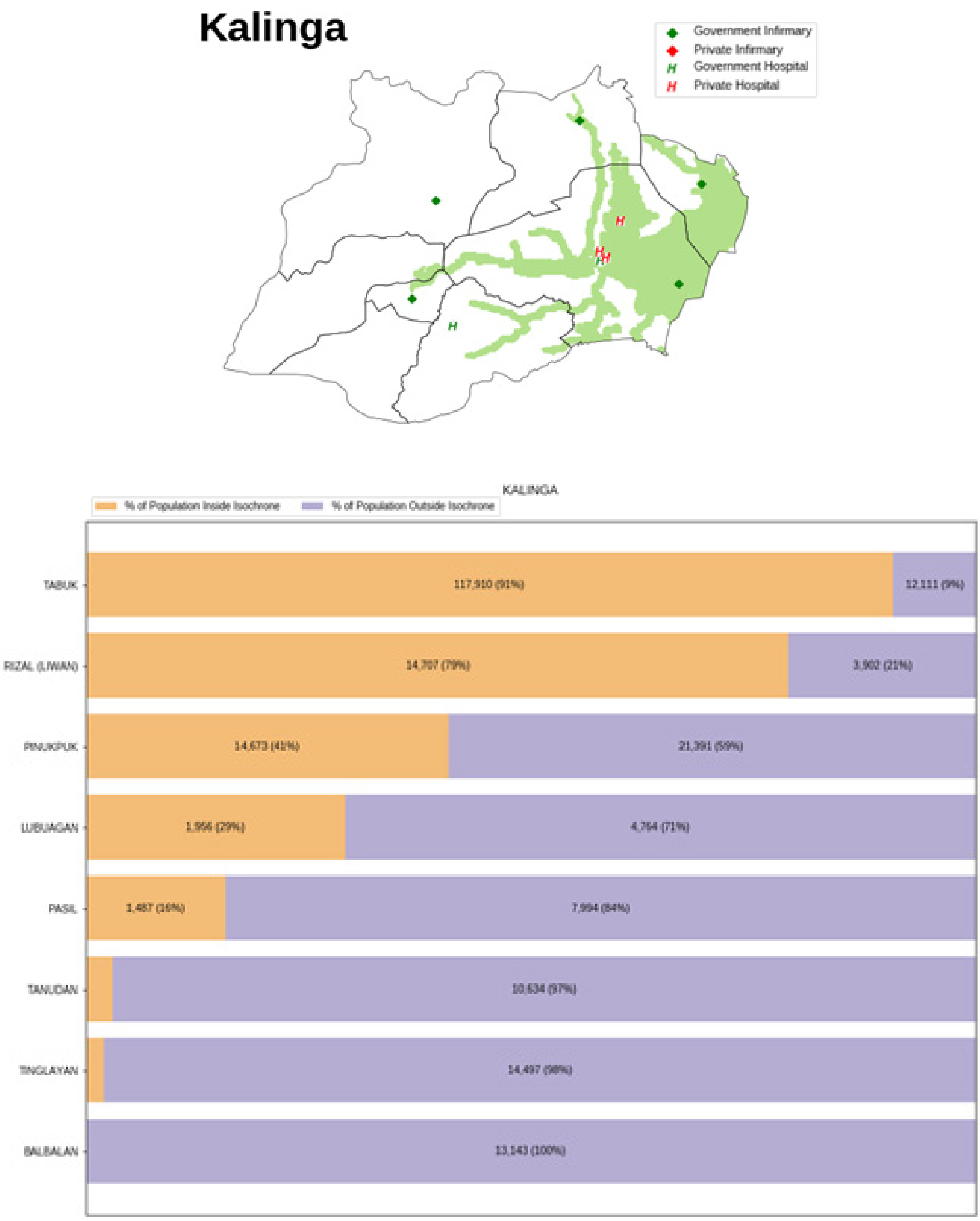

Figure 47. Kalinga Hospital Access Map

CHAPTER IX

Monitoring and Evaluation Plan

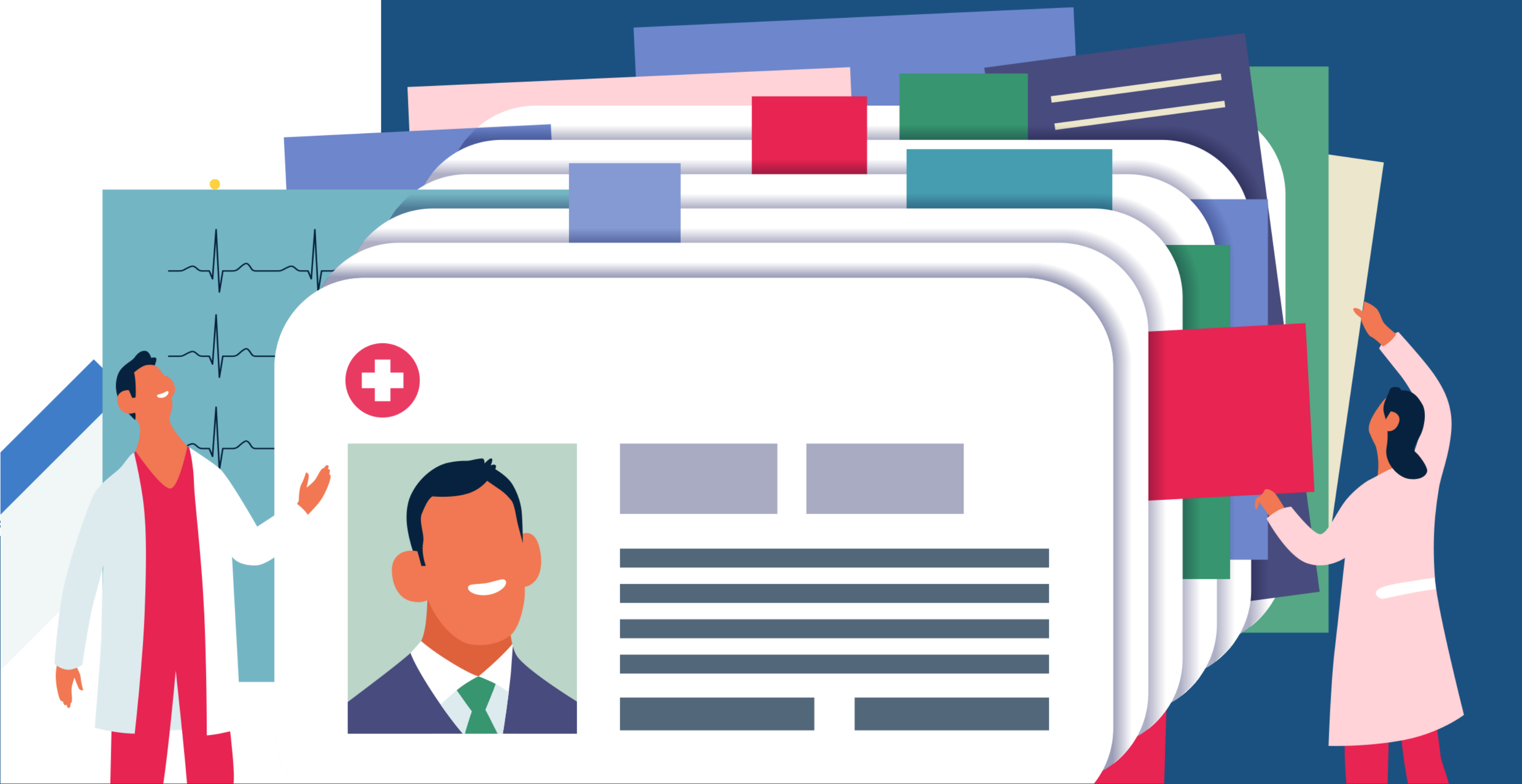

This chapter outlines the monitoring and evaluation framework for the PHFDP 2020-2040. The M&E component of the Plan was adopted from the IHP+ Framework, which comprises four (4) major indicators: system inputs and processes, outputs, and outcomes (intermediate and final).

The framework shows how inputs to the system and processes are reflected in outputs and eventual outcomes. This results-chain framework can be used to demonstrate performance of interventions. Here, the Plan assumes that inputs such as capital outlay subsidies with service delivery reforms will lead to higher quantity and quality of healthcare facilities and eventually improved healthcare utilization and access.

Table 48. Monitoring and Evaluation Framework

	Inputs	Outputs	Outcomes (Intermediate)	Outcomes (Final)
Indicator Domain	Funding<br>Human Resources<br>Governance	Availability of services<br>Quality of services	Utilization and access	Health status<br>Financial protection<br>Responsiveness
Data Domain	Administrative/ accounting data	Administrative data	Administrative data/ survey	

How does this framework translate to core indicators? Inputs and process indicators are generally related to funding (that is HFEP resources, local government resources) and capacity building (that is training). These inputs should be translated to outputs, which are mostly the physical availability of health facilities. The intermediate outcomes are mostly need-based indicators such as utilization and access to health facilities.

In IHP+ Framework, final outcomes are typically monitored. However, health status, financial protection and responsiveness are now measured elsewhere (please refer to National Objectives for Health 2017-2022). Tables 55-57 present the different indicators for each domain.

Table 49. Input/Process Indicators

Inputs	Formula	Disaggregation	Purpose	Duration	Data source
HFEP spending per capita	Total HFEP expenditure/population	Province/ HUC/ ICC	To determine the HFEP spending in relation to population size	Yearly	DOH Accounting/ HFEP data
HFEP disbursement rates	HFEP expenditure/ HFEP allocation	Province/ HUC/ ICC	To determine the absorp-tive capacity of HFEP	Yearly	DOH Accounting/ HFEP data
HFEP spending on primary care	HFEP spending on BHS and RHU/ Health Cen-ter/Total HFEP spending	Province/ HUC/ ICC, disaggregat-ed by type	To determine if primary care network facilities are prioritized	Yearly	DOH Accounting/ HFEP data
HFEP Spending on Special Facilities	HFEP spending on Special Facilities/ Total HFEP spending	By category of Special Facilities, disaggregated by subtype/s	To determine how much of the HFEP resources are allocated for Special Facil-ities	Yearly	DOH Accounting/ HFEP data

HFEP Spending relat-ed to resilience in facilities	HFEP spending on resil-ience/Total HFEP spend-ing	Province/ HUC/ ICC	To determine how much of the HFEP resources are allocated to resilience projects	Yearly	DOH HFEP data
HFEP projects with complete staff work	HFEP project with com-plete staff work/Total HFEP projects	Province/ HUC/ ICC	To determine the readi-ness and level of planning of local governments	Yearly	DOH HFEP data
HFEP spending in accordance with prioritization matrix	HFEP expenditure that followed prioritization matrix	Region/ Prov-ince/ HUC/ ICC	To determine effective equitable allocation of financing	Yearly	DOH HFEP data
Local government spending on health infrastructure (hospi-tal), per capita	Health infrastructure (hospital) project/Total population	Province/HUC/ ICC by category, disaggregated by type	To determine the level of local government spend-ing on health infrastruc-ture (hospital)	Yearly	Accounting office of LGUs
Local government spending on health equipment (hospital), per capita	Health equipment (hos-pital) project/Total population	Province, HUC/ ICC by category, disaggregated by type	To determine the level of local government spend-ing on health equipment (hospital)	Yearly	Accounting office of LGUs
Local government spending on primary care facilities and BHS (infrastructure)	Health infrastructure spending on primary care/Total Health infra-structure	Province, HUC/ ICC by category, disag-gregated by type	To determine the level of prioritization of provinces towards primary care infrastructure	Yearly	Accounting office of LGUs
Number of LGUs received training on resource mobilization, strategic planning, and contracting, and PPP	Number of technical assistances provided to LGUs by DOH, disaggregated by type of assistance<br><br>Client satisfaction rating for technical assistance provided	Province, HUC/ ICC, by category	To determine the per-formance of DOH in providing effective tech-nical assistance to LGUs	Yearly	Data from DOH HFDB, BLHSD, PPP Office and other relevant offices
Number of facilities assessed using Hospi-tal Safety and Resili-ence Framework/ Index	Number of facilities assessed/ Total number of facilities (by type of facility)	Province/ HUC/ ICC	To determine the rate of completion of assessing health facilities for safety and climate/ disaster resilience	Yearly	DOH HFDB Data

<u>Table 50. Output Indicators</u>

Outputs	Formula	Disaggregation	Purpose	Duration	Data source
BHS to barangay ratio	Number of BHS/ Number of barangays	Province/HUC/ ICC	To determine wheth-er the target number of BHS was met per province/ HUC/ ICC	Yearly	DOH HFDB data, KMITS National Health Facility Registry (NHFR)
Number of provinces/ cities with PCF within 30 minutes	Number of provinces/ cities with PCF accessible within 30 minutes by 80% of the population/ Total number of provinces	Province/HUC/ ICC	To determine wheth-er the target for ac-cess to PCF was met	Yearly	DOH Geospatial analysis

Percent PCF established	Number of PCF established/ Number of total gaps	Province/HUC/ ICC	To determine the performance in meeting gaps in PCF	Yearly	DOH HFDB, DOH HFSRB data, NHFR
Bed to population ratio	(Number of bed /Total pop-ulation) x 1000	Province/HUC/ ICC	To determine whether the target number for hospital beds was met	Yearly	DOH Accounting/ HFEP data
Number of provinces/ cities with hospitals accessible within 1 hour	Number of provinces/ cities with hospitals accessible within 1 hour by 80% of the population/ Total number of provinces	Province/HUC/ ICC	To determine wheth-er the target for access to hospitals was met	Yearly	DOH Geospatial analysis
Percent of L1 hospital beds established	Number of L1 hospital beds established/ Number of total gaps	Province/HUC/ ICC	To determine the performance in meeting gaps in hospital beds, per level, and compare with targets	Yearly	DOH HFDB, DOH HFSRB data, NHFR
Percent of L2 hospital beds established	Number of L2 hospi-tal beds established/ Number of total gaps	Province/HUC/ ICC	To determine the performance in meeting gaps in hospital beds, per level, and compare with targets	Yearly	DOH HFDB, DOH HFSRB data, NHFR
Percent of L3 hospital beds established	Number of L3 hospi-tal beds established/ Number of total gaps	Province/HUC/ ICC	To determine the performance in meeting gaps in hospital beds, per level, and compare with targets	Yearly	DOH HFDB, DOH HFSRB data, NHFR
Percent of Specialty Centers established	Number of Specialty Cen-ters established/ Total num-ber of targeted Specialty Centers	National	To determine the peformance in meeting gaps in specialty care	Yearly	DOH HFDB
Percent of Special-ized Laboratories estab-lished	Number of Specialized La-boratories established/Total number of targeted	National	To determine the performance in meeting gaps	Yearly	DOH HFDB
Percent of Blood Service Facilities established	Number of Blood Service Facilities established/Total number of targeted	National	To determine the performance in meeting gaps	Yearly	DOH HFDB
Percent of Drug Abuse Treatment and Rehabilitation Facili-ties estab-lished	Number of Drug Abuse Treatment and Rehabilita-tion Facilities estab-lished/ Total number of targeted	National	To determine the performance in meeting gaps	Yearly	DOH HFDB
MRI per million popu-lation	(Number of MRI/Total Popu-lation) *1 million, distrib-uted by region	Region, by hospital ownership	To determine the availability and distribution of MRI in the country	Yearly	DOH HFDB, FDA data

CT scan per million population	(Number of MRI/Total Popu-lation) *1 million, distrib-uted by region	Region, by hospital ownership	To determine the availability and distribution of CT scan in the country	Yearly	DOH HFDB, FDA data
LINAC per million population	(Number of LINAC/ Total Population) *1 million, distributed by region	Region, by hospital ownership	To determine the availability and distribution of LINAC in the country	Yearly	DOH HFDB, PNRI data
Share of resilient health facilities	Number of resilient health facilities/Total health Facilities	Province, by priority province, by type of health facility	To determine the share of health facilities considered 'resilient' (based on standard).	Yearly	DOH HFDB data

Table 51. Intermediate Outcome Indicators

Outputs	Formula	Disaggregation	Purpose	Duration	Data source
Healthcare utili-za-tion rate	(Number of users of any facility/ (Total Population*illness factor)	Province (if posible) or regional, by type/ level of facility	To determine whether healthcare used (conditional to illness) has increased	Yearly	Household survey
Average time to access health facility	Average time (in minutes) to travel the nearest facility depending on the type	Province or region, by socio-economic sta-tus, type of residence	To determine whether the population has access to PCF (30 minutes) and hospitals (1 hour)	Yearly	Household survey
Utilization of primary care	Number of users of primary care network facility /Total population*illness factor)<br>Qualitative: Analysis of health seeking behavior	Province or region, by socio-economic status, type of residence	To determine whether primary care used (conditional to illness) has increased<br>To determine access of population to primary care as entry point into health system	Yearly	Household survey
Proportion of patients referred to hospital from PCF or health stations	Share of inpatient who visited health stations or PCF	Province, by priority province, by health facility type.	To determine how much is allocated of the HFEP resources are allocated to resilience.	Yearly	Household survey

APPENDIX

Annex A: Local Government Unit (LGU) Profiles

Annex B: Access Maps

Annex C: Resource-Stratified Frameworks

Annex D: Designated National Specialty Centers, Advanced Comprehensive Centers, and Basic Comprehensive Centers, Target Year of Establishment, and Estimate Costs for Each Specialty

Annex E: Resource Stratified Framework for Blood Service Facilities (BSF)

Annex F: Development Plan for Blood Centers

Annex G: Resource Stratified Framework for Drug Abuse Treatment and Rehabilitation Facilities

Annex H: Contributors and Members of Technical Working Groups

Blood Service Facilities: Dr. Criselda Abesamis, Dr. Andres Bonifacio, Ms. Marites Estrella, Dr. Christie Monina Nalupta, Mr. Renato Hapan, Mr. Salvador Aydante Jr., Ms. Sheryl Tumamao, Ms. Diosa Caringal, Ms. Aiza Marie Advincula

Drug Abuse Treatment and Rehabilitation Facilities: Mr. Jose Bienvenido Leabres, Mr. Reymundo De Guzman, Jr, Ms. Dianne Mae Rivera

Military Facilities: Col. Fatima Navarro

Specialty Centers Technical Working Groups:

- **Lung Care:** Dr. Vincent M. Balanag, Jr. (Chairperson), Ms. Faye Diana C. Chua, Dr. Ma. Fatima De Guzman, Dr. Sullian S. Naval, Engr. Aries Ocomen
- **Renal Care:** Dr. Rose Marie O. Liquete (Chairperson), Mr. Learsi Ray Afable, Dr. Gabrielle Ann Dela Paz, Dr. Luis Limchiu, Jr., Dr. Rosalie Paje, Dr. Francisco Sarmiento
- **Cardiovascular Care:** Dr. Gerardo S. Manzo (Chairperson), Dr. Joel Abanilla, Mr. Learsi Ray Afable, Dr. Juliet Balderas, Ms. Glorilyn Joy Carolino, Dr. Carmela Granada, Ms. Zenaida Villaluna
- **IDTM:** Dr. Rontgene Solante (Chairperson), Dr. Mari Rose Delos Reyes (Co-chair), Dr. Celia Carlos, Ms. Catherine Dimaano, Dr. Rosanna Ditangco, Dr. Roberto Guanzon, Dr. Edmundo Lopez, Ms. Joy Padrigano, Dr. Mario Panaligan, Dr. Beatriz Quiambao, Dr. Charissa Tabora, Dr. Amado Tandoc
- **Orthopedic Care:** Dr. Paul San Pedro (Chairperson), Dr. David Alagar, Dr. Terence John Antonio, Ms. Phoebe C. Cabbab, Dr. Arturo Cañete, Dr. Miles Dela Rosa, Dr. Frederic Diyco, Dr. Manolito Flavier, Dr. William Lavadia, Engr. Aries Ocomen, Ms. Maria Christina Raymundo
- **Physical Rehabilitation Medicine:** Dr. Eulalia Beredo (Chairperson), Dr. Terence John Antonio, Ms. Phoebe C. Cabbab, Dr. Raul Michael Cembrano, Dr. Maria Regina Espinosa, Engr. Aries Ocomen, Ms. Maria Christina Raymundo
- **Toxicology:** Dr. John Paul Ner (Chairperson), Dr. Visitacion Antonio, Dr. Tisha Ysabel Briola, Ms. Catherine Dimaano, Dr. Leslie Garcia, Ms. Angelita Jimenez, Dr. Lyn Crisanta Panganiban, Dr. Ella Joy Perez, Dr. Cherrie Grace Quingking, Dr. Rhodora Reyes, Michael Anthony Rioflorido, Engr. Jocelyn Soria
- **Burn and Trauma:** Dr. Joel U. Macalino (Chairperson), Dr. Ramon Anatalio III, Dr. Terence John Antonio, Dr. Trishalyn Mae Correa, Dr. Lora Mae de Guzman, Dr. Glenn Angelo Genuino, Dr. Aireen Patricia Madrid, Mr. Joseph John Madrilejos, Dr. Napoleon Obaña, Dr. Orlando Ocampo, Dr. Raymundo Resurrecion, Ms. Liane May Rivas, Dr. Ronnie Torres, Dr. Maria Rosario Sylvia Uy
- **Mental Health:** Dr. Beverly Azucena (Chairperson), Ms. Ivy Grace Agus, Dr. Rodney Boncajes, Dr. Agnes Casino, Ms. Frances Prescilla L. Cuevas, Dr. Gabrielle Ann Dela Paz
- **Cancer Care:** Dr. Corazon Ngelangel (Chairperson), Ms, Mary Grace Fermo, Ms. Danielle Imperio, Dr. Rosalie Paje, Dr. Caroline Mae Ramirez
- **Brain:** Dr. Maria Victoria Manuel (Chairperson), Dr. Michael Sabalza (Co-chair), Dr. Gabrielle Ann Dela Paz, Dr. Dianne Kay Ferrer, Dr. Carmela Granada, Ms. Liane May Rivas, Dr. Ignacio Rivera, Dr. Jo Ann Soliven
- **Geriatric Care:** Wenceslao Llauderes (Chairperson), Edgardo Sarmiento (Co-chair), Ms. Dianne Melody De Roxas, Dr. Shelley Dela Vega, Dr. Doris Mariebel Camagay, Dr. Maria Isabelita Estrella, Dr. Edwin Fortuno, Dr. Lydia Manahan, Mr. Roderick Napulan, Dr. Eduardo Poblete, Dr. Miguel Ramos, Engr. June Philip Ruiz, Dr. Minerva Vinluan
- **Dermatology:** Dr. Ma. Angela Lavadia (Chairperson), Dr. Deejay Arcega, Dr. Ma. Flordeliz Casintahan, Dr. Ma. Teresita Gabriel, Dr. May Gonzales, Ms. Danielle Imperio, Ms. Joy Padrigano, Dr. Roberto Pascual, Dr. Liza Maria Paz-Tan, Dr. Vilma Pelino, Dr. Francisco Rivera IV, Dr. Julie Mart Rubite
- **Eye:** Dr. Ma. Victoria Rondaris (Chairperson), Dr. Rainier Covar, Ms. Danielle Imperio, Dr. Roberto Julian, Dr. Shelley Ann Mangahas, Dr. Andrea Pajarillo, Ms. Clara Francesca Roa, Dr. Reynaldo Santos, Dr. Edgardo Sarmiento, Dr. Prospero Ma. Tuano, Dr. Amelia Reyes-Vera Cruz, Dr. Maria Rosario Sylvia Uy
- **Neonatology:** Dr. Julius Lecciones (Chairperson), Ms. Ivy Grace Agus, Dr. Anthony Calibo, Mr. Erickson Feliciano, Dr. Sonia Gonzales, Dr. Sheila Ann Masangkay, Dr. Michael Resurreccion
- **Administrative Support to all TWGs:** Ms. Joan Guevara, Ms. Sheila Mae Labongray

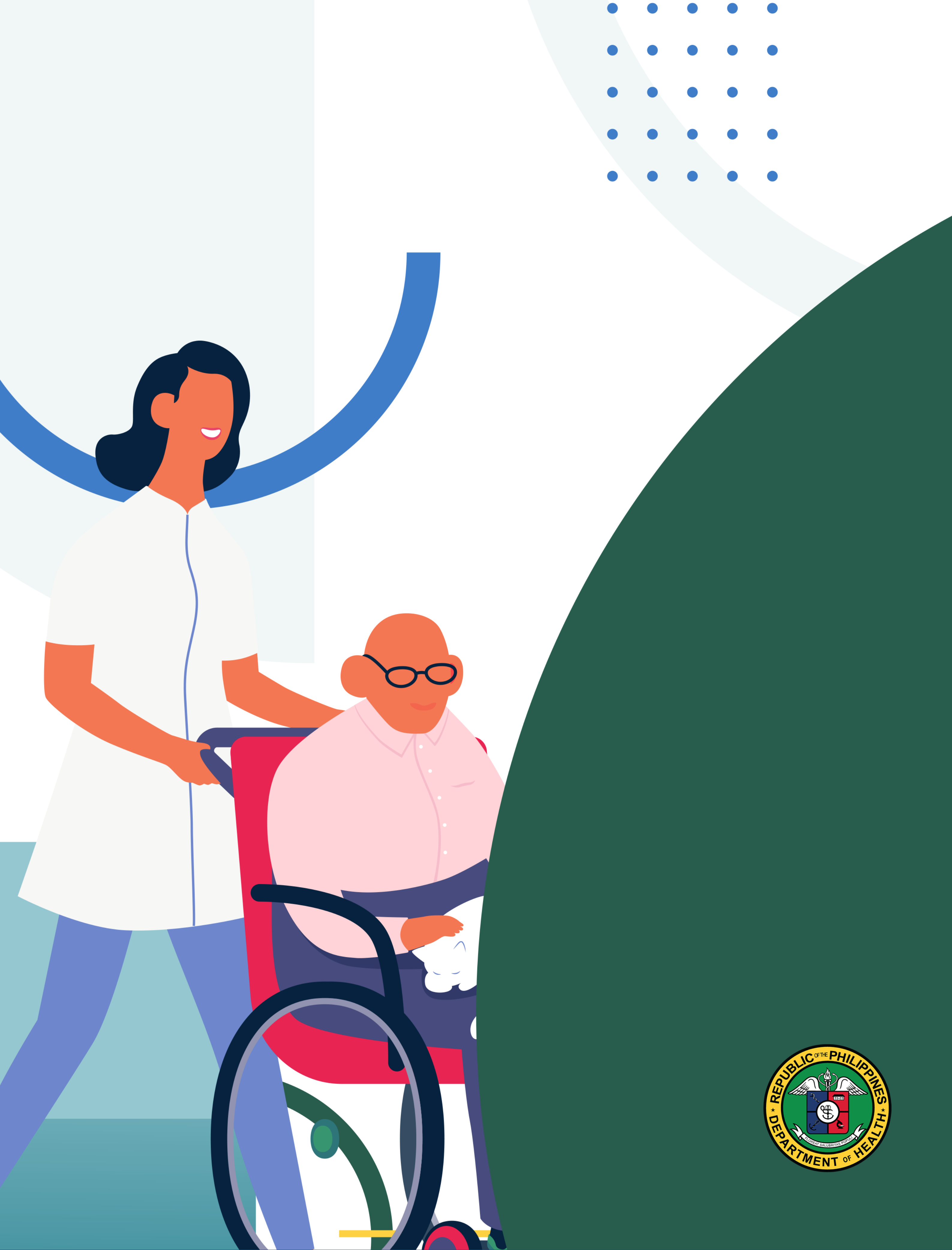

REPUBLIC OF THE PHILIPPINES
DEPARTMENT OF HEALTH
